# Multiubiquitination of TRPV4 reduces channel activity independent of surface localization

**DOI:** 10.1016/j.jbc.2022.101826

**Published:** 2022-03-14

**Authors:** William H. Aisenberg, Brett A. McCray, Jeremy M. Sullivan, Erika Diehl, Lauren R. DeVine, Jonathan Alevy, Anna M. Bagnell, Patrice Carr, Jack K. Donohue, Benedikt Goretzki, Robert N. Cole, Ute A. Hellmich, Charlotte J. Sumner

**Affiliations:** 1 Department of Neurology, Johns Hopkins University School of Medicine, Baltimore, Maryland, USA; 2 Department of Chemistry, Biochemistry Section, Johannes Gutenberg-Universität Mainz, Mainz, Germany; 3 Department of Biological Chemistry, Johns Hopkins University School of Medicine, Baltimore, Maryland, USA; 4 Institute of Organic Chemistry and Macromolecular Chemistry, Cluster of Excellence 'Balance of the Microverse', Friedrich-Schiller-Universität, Jena, Germany; 5 Center for Biomolecular Magnetic Resonance (BMRZ), Goethe-Universität, Frankfurt am Main, Germany; 6 The Solomon H. Snyder Department of Neuroscience, Johns Hopkins University School of Medicine, Baltimore, Maryland, USA

**Keywords:** TRPV4, ion channel, ubiquitination, plasma membrane, Charcot–Marie–Tooth disease, channel activation, NEDD4, AGC, automatic gain control, ARD, ankyrin repeat domain, BSA, bovine serum albumin, CTD, C-terminal IDR, DUB, deubiquitinating enzyme, EGFP, enhanced GFP, FDR, false discovery rate, GSK101, GSK1016790A, HA, hemagglutinin, HA-Ub, HA-tagged Ub, HC067, TRPV4-specific antagonist HC067047, HEK293T, human embryonic kidney 293T cell line, HRP, horseradish peroxidase, HTS, hypotonic saline, ICC, immunocytochemistry, IDR, intrinsically disordered region, IP, immunoprecipitation, MDCK, Madin–Darby canine kidney, MN-1, motor neuron–like 1, MS, mass spectrometry, PACSIN, protein kinase C and casein kinase substrate in neuron, PBD, PIP2-binding domain, PRR, proline-rich region, PTM, post-translational modification, RIPA, radioimmunoprecipitation assay, TBST, Tris-buffered saline containing 0.1% Tween-20, TEV, tobacco etch virus, TIRF, total internal reflection fluorescence, TMT, tandem mass tag, TRPV4, transient receptor potential vanilloid 4, TRPV4-FLAG, FLAG-tagged TRPV4, Ub, ubiquitin, WB, Western blot

## Abstract

Ubiquitin (Ub)-mediated regulation of plasmalemmal ion channel activity canonically occurs *via* stimulation of endocytosis. Whether ubiquitination can modulate channel activity by alternative mechanisms remains unknown. Here, we show that the transient receptor potential vanilloid 4 (TRPV4) cation channel is multiubiquitinated within its cytosolic N-terminal and C-terminal intrinsically disordered regions (IDRs). Mutagenizing select lysine residues to block ubiquitination of the N-terminal but not C-terminal IDR resulted in a marked elevation of TRPV4-mediated intracellular calcium influx, without increasing cell surface expression levels. Conversely, enhancing TRPV4 ubiquitination *via* expression of an E3 Ub ligase reduced TRPV4 channel activity but did not decrease plasma membrane abundance. These results demonstrate Ub-dependent regulation of TRPV4 channel function independent of effects on plasma membrane localization. Consistent with ubiquitination playing a key negative modulatory role of the channel, gain-of-function neuropathy-causing mutations in the *TRPV4* gene led to reduced channel ubiquitination in both cellular and *Drosophila* models of TRPV4 neuropathy, whereas increasing mutant TRPV4 ubiquitination partially suppressed channel overactivity. Together, these data reveal a novel mechanism *via* which ubiquitination of an intracellular flexible IDR domain modulates ion channel function independently of endocytic trafficking and identify a contributory role for this pathway in the dysregulation of TRPV4 channel activity by neuropathy-causing mutations.

Plasma membrane–localized ion channels enable cells to rapidly respond to intracellular and extracellular stimuli but require tight regulatory control to maintain cellular homeostasis. Indeed, multiple disorders including neurological diseases are caused by genetic mutations that alter ion channel activity and cellular ion influx (1, 2, 3, 4). Dynamic regulatory control of plasmalemmal ion channels is ensured by modulation of channel gating as well as by changes in cell surface expression.

Ubiquitination is a rapidly reversible post-translational modification (PTM) shown to regulate the activity of multiple plasma membrane–localized ion channels (4). The covalent attachment of an 8 kDa ubiquitin (Ub) peptide to a lysine residue is catalyzed by the sequential action of a Ub-activating enzyme (E1), a Ub-conjugating enzyme (E2), and a Ub ligase (E3), the last of which provides specificity for the substrate protein. For a given substrate, Ub may be conjugated to a single lysine (monoubiquitination), multiple lysines (multiubiquitination), or be ubiquitinated itself to form Ub chains (polyubiquitination). It is well established that monoubiquitination/multiubiquitination of surface-expressed ion channels can stimulate removal of a channel from the plasma membrane, thereby negatively regulating channel activity (5, 6, 7, 8). Conversely, disrupting ubiquitination of such ion channels typically results in the accumulation of the channel at the plasma membrane, leading to a gain-of-channel function (9, 10, 11, 12, 13). In cases in which ion channels are internalized after ubiquitination, they may then be shuttled to proteolytic pathways (7, 14) or trafficked to recycling compartments that are available for membrane reinsertion (15, 16, 17). In addition to regulating membrane protein trafficking and degradation, monoubiquitination or multiubiquitination events are increasingly appreciated to regulate protein function in other ways, including by altering protein–protein and protein–lipid interactions (18, 19, 20). Individual proteins may also undergo multiple distinct Ub modifications that together provide a diverse Ub architecture enabling complex and dynamic regulation of cellular processes (21, 22, 23). NEDD4 and ITCH, members of the NEDD4 family of E3 Ub ligases, acting alone or with adaptor/accessory proteins, have been shown to ubiquitinate several plasmalemmal proteins, including ion channels, resulting in their internalization (24). It has not been determined whether monoubiquitination/multiubiquitination of an ion channel can affect channel activity independent of internalization.

Transient receptor potential vanilloid 4 (TRPV4), a member of the vanilloid subfamily of TRP channels, is a broadly expressed calcium-permeable ion channel with diverse functions (25). TRPV4 functions as a homotetramer at the plasma membrane where it can be activated by mechanical stimulation, hypo-osmotic stress, arachidonic acid, and synthetic compounds (25). The channel is composed of six transmembrane domains flanked by intracellular N-terminal and C-terminal domains, the structures of which have been partly characterized by cryogenic electron microscopy (26), including the large cytosolic N-terminal ankyrin repeat domain (ARD). Dominant gain-of-function mutations of exposed arginine residues on one face of the ARD cause forms of peripheral neuropathy, including Charcot–Marie–Tooth disease type 2C and distal spinal muscular atrophy (27, 28, 29, 30). Though our understanding of how these missense mutations cause a gain of function is incomplete, they are not thought to increase channel surface expression (29, 31) but may rather disrupt specific regulatory protein–protein and/or protein–lipid interactions (32). In contrast to the ARD, the intrinsically disordered regions (IDRs) of the N termi– and C termini have not been amenable to structural analysis (33). The function of IDRs, which have no stable ordered structure, is not fully understood, although they are increasingly recognized as important components of the cell signaling machinery as they contain multiple interaction motifs, dynamically bind diverse targets, and act as central hubs in signaling networks (34). The TRPV4 N-terminal IDR is located upstream of the ARD and contains a PI(4,5)P_2_- (hereafter PIP_2_) binding domain (PBD) and a proline-rich region (PRR) (33, 35, 36). Intermolecular interactions at these domains modulate TRPV4 channel activity without altering TRPV4 surface density (36, 37). IDRs are frequently post-translationally modified (33), but whether ubiquitination within the IDR affects TRPV4 function is unknown.

Here, we show that TRPV4 is multiubiquitinated within the intracellular flexible N-terminal IDR and that blocking ubiquitination at key residues within this domain results in increased channel activity without increasing TRPV4 surface abundance. Conversely, enhancing ubiquitination at these residues reduces channel activity without reducing plasma membrane abundance. We further demonstrate that neuropathy-causing mutations in TRPV4 reduce channel ubiquitination correlating with increased channel activity, but enhancing ubiquitination of neuropathy mutants dampens their responses to agonist stimulation. These data reveal a novel mechanism by which multiubiquitination of a disordered intracellular protein domain can regulate ion channel activity without altering plasma membrane abundance and provide insight into mechanisms contributing to TRPV4 gain-of-function mutations.

## Results

### TRPV4 is monoubiquitinated/multiubiquitinated in multiple cell types

**Figure 1:**
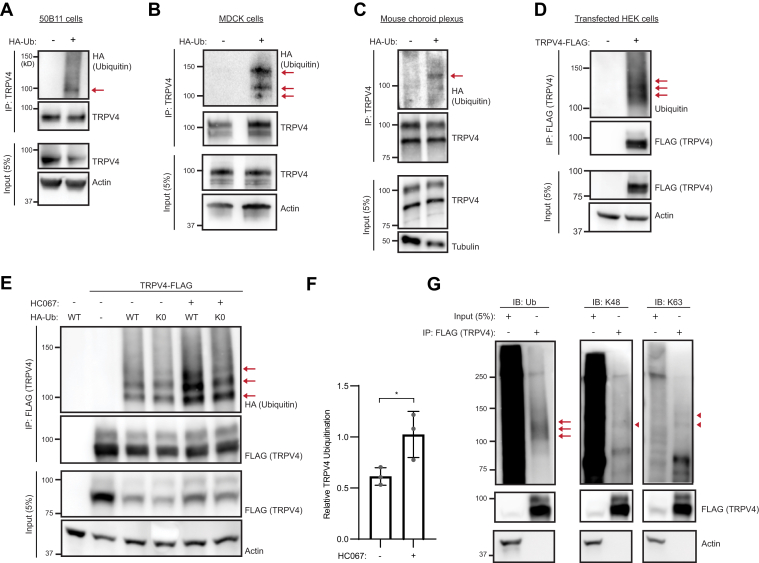
**TRPV4 is monoubiquitinated/multiubiquitinated.***A* and *B*, ubiquitination of endogenous TRPV4 in HA-ubiquitin (Ub)-expressing 50B11 (*A*) and MDCK (*B*) cells, as detected by immunoprecipitation (IP) with anti-TRPV4 antibody and Western blot analysis with anti-HA antibody. *C*, Western blot analysis using anti-HA antibody of endogenous TRPV4 immunoprecipitated from the choroid plexus of HA-Ub mice; parallel IP from WT mice demonstrates the specificity of the anti-HA antibody. *D*, cell-based ubiquitination assay performed using HEK293T cells transiently transfected with TRPV4-FLAG. TRPV4 was immunoprecipitated from whole cell lysates with anti-FLAG antibody and Western blot analysis performed with anti-Ub antibody. *E*, cell-based ubiquitination assay utilizing HEK293T cells cotransfected with TRPV4-FLAG and either WT-HA-Ub or K0-HA-Ub, in the presence or the absence of the TRPV4-specific antagonist HC067 (0.5 μM). TRPV4 was immunoprecipitated from whole cell lysates with anti-FLAG antibody, and Western blot analysis was performed with anti-HA antibody. *F*, quantification of relative TRPV4 ubiquitination in the presence or the absence of HC067 from the experiment shown in *E*. n = 3 independent transfections. Data are presented as means ± SD. ∗*p* = 0.0422 (unpaired *t* test). *G*, cell-based ubiquitination assay performed using HEK293T cells transiently transfected with TRPV4-FLAG. TRPV4 was immunoprecipitated from whole cell lysates with anti-FLAG antibody, and Western blot analysis was performed with anti-Ub, anti-K48-linkage-specific polyubiquitin, or anti-K63-linkage-specific polyubiquitin antibodies. *Arrows* indicate ubiquitinated TRPV4 and *arrowheads* indicate polyubiquitinated TRPV4. HA, hemagglutinin; HC067, TRPV4-specific antagonist HC067047; HEK293T, human embryonic kidney 293T cell line; MDCK, Madin–Darby canine kidney; TRPV4, transient receptor potential vanilloid 4; TRPV4-FLAG, FLAG-tagged TRPV4.

To determine whether endogenous TRPV4 protein is ubiquitinated in cells and whole tissue, we assessed an immortalized rat sensory neuron cell line (50B11 cells), the Madin–Darby canine kidney (MDCK) epithelial cell line, and mouse choroid plexus tissue; all of which express robust levels of TRPV4 (32, 38, 39). To immunopurify endogenous TRPV4, we used an anti-TRPV4 antibody that detects TRPV4 protein in cultured primary choroid plexus epithelial cells (40), kidney, and choroid plexus derived from WT, but not *Trpv4* null mice (40, 41, 42) (Fig. S1, *A* and *B*). Using this antibody, we achieved robust TRPV4 immunoprecipitation (IP) with little background compared with whole cell lysate inputs (Fig. S1*C*). TRPV4 banding patterns can vary across tissues and cell lines likely because of variations in PTM including glycosylation (43). Our data showed a single TRPV4 band in 50B11 cells (Fig. 1*A*) and a doublet in MDCK cells and choroid plexus tissue (Fig. 1, *B* and *C*) consistent with previous studies (39, 44, 45, 46, 47).

To facilitate detection of ubiquitinated TRPV4, hemagglutinin (HA)-tagged Ub (HA-Ub) was expressed in 50B11 cells using lentiviral transduction, MDCK cells *via* transient transfection, and in C57BL/6J mice *via* gene targeting (48). Western blot (WB) analyses of TRPV4 immunoprecipitated from the two cell lines showed a single mass-shifted Ub band in 50B11 cells (Fig. 1*A*, *arrow*) and three mass-shifted bands in MDCK cells (Fig. 1*B*, *arrows*) following probing with anti-HA antibody. WB analyses of TRPV4 immunoprecipitated from choroid plexus tissues of HA-Ub-expressing mice, but not WT mice, demonstrated a mass-shifted Ub band (Fig. 1*C*). Together, these data indicate that endogenous TRPV4 protein is ubiquitinated in multiple cell types, both *in vitro* and *in vivo*.

To explore TRPV4 ubiquitination in depth, we established a system that allowed us to readily scale up cell-based Ub detection assays for downstream proteomics analyses. C-terminally FLAG-tagged TRPV4 (TRPV4-FLAG) was immunoprecipitated from transfected human embryonic kidney 293T (HEK293T) cells, separated on SDS-PAGE gels, and blotted for endogenous Ub. We detected multiple discrete Ub bands in the 100 to 140 kDa range, consistent with either polyubiquitination or multiubiquitination of TRPV4 (Fig. 1*D*, *arrows*). To assess the type of Ub linkage conjugated to TRPV4, we cotransfected cells with TRPV4-FLAG and either HA-Ub or a form of HA-Ub that cannot form poly-Ub chains (K0-HA-Ub) (49) (Fig. 1*E*). Comparable banding patterns of ubiquitinated TRPV4 were observed in cells expressing either HA-Ub or K0-HA-Ub (Fig. 1*E*, *arrows*), indicating that TRPV4 is predominantly multiubiquitinated under these conditions, consistent with the findings of a previous study (50). To determine whether the pattern of ubiquitination of TRPV4 is influenced by channel activity, transfected cells were cultured in the presence of the TRPV4-specific antagonist HC067047 (hereafter HC067) (51). The TRPV4 Ub banding pattern was not altered by the presence of HC067, indicating that basal TRPV4 channel activity does not play a significant role in the type of channel ubiquitination. However, the amount of ubiquitinated TRPV4 increased in the presence of antagonist suggesting a link between TRPV4 channel activity and its level of ubiquitination (Fig. 1*F*). To further confirm that TRPV4 was predominantly multiubiquitinated, WB analyses were performed on immunoprecipitated TRPV4 using antibodies specific for either K48-linked or K63-linked poly-Ub chains. Although diffuse poly-Ub signals were observed in the unprecipitated lysates (inputs), minimal signal was seen in immunoprecipitated TRPV4 eluates (Fig. 1*G*). Together, these data suggest that TRPV4 is predominantly multiubiquitinated.

### TRPV4 is ubiquitinated at lysine residues in its cytoplasmic intrinsically disordered regions

**Figure 2:**
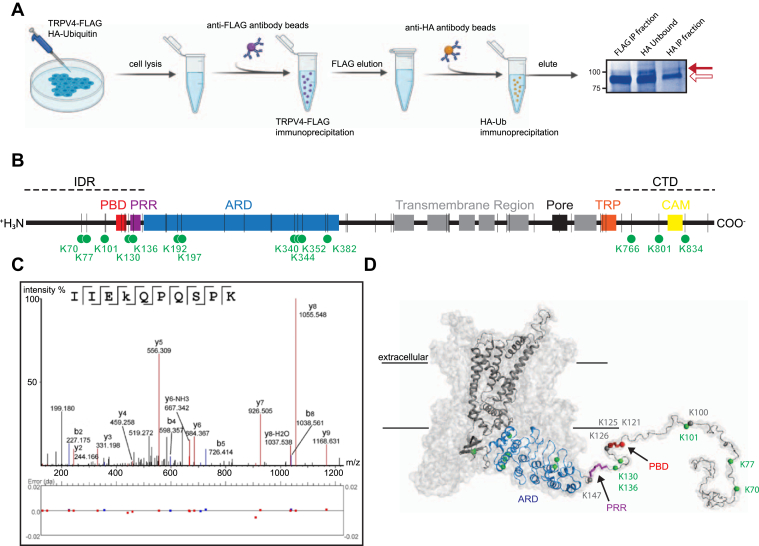
**TRPV4 is ubiquitinated within its N-terminal intrinsically disordered region.***A*, schematic of the double IP workflow utilized to purify ubiquitinated TRPV4 from HEK293T cells cotransfected with TRPV4-FLAG and HA-ubiquitin. TRPV4 was initially immunoprecipitated using anti-FLAG antibody (FLAG IP fraction), and ubiquitinated TRPV4 was then specifically isolated by IP with anti-HA antibody (HA IP fraction). The image at *far right* shows the two immunoprecipitated fractions as well as TRPV4 that did not immunoprecipitate with anti-HA antibody (HA unbound), on a Coomassie blue–stained gel; *open arrow* indicates unmodified TRPV4, *solid arrow* indicates ubiquitinated TRPV4. *B*, linear schematic of a TRPV4 monomer with functional domains highlighted: the N-terminal IDR (IDR) includes the PBD and PRR, which precedes the ARD. The CTD follows the TRP and contains the CAM. The *vertical black lines* show the positions of all 45 lysine residues in the TRPV4 protein sequence; *green circles* indicate ubiquitinated residues identified by mass spectrometry. *C*, representative mass spectrum demonstrating TRPV4 ubiquitination at K130. *D*, model of a TRPV4 tetramer, with a single monomer highlighted (Protein Data Bank ID: 6BBJ). Lysines within the N-terminal IDR are identified as *spheres*, with ubiquitinated residues indicated in *green* nonubiquitinated residues in *gray*. The PBD is shown in red, and the PRR is shown in *purple*. ARD, ankyrin repeat domain; CAM, calmodulin-binding domain; CTD, C-terminal IDR; HA-ubiquitin, hemagglutinin-tagged ubiquitin; HEK293T, human embryonic kidney 293T cell line; IDR, intrinsically disordered region; IP, immunoprecipitation; PBD, PIP_2_-binding domain; PRR, proline-rich region; TRP, TRP box domain; TRPV4, transient receptor potential vanilloid 4; TRPV4-FLAG, FLAG-tagged TRPV4.

Mass spectrometry (MS) analysis of immunoprecipitated TRPV4 protein was performed to identify ubiquitinated lysine residues. We enriched for ubiquitinated TRPV4 by using a double IP approach (Fig. 2*A*) utilizing HEK293T cells transfected with both TRPV4-FLAG and HA-Ub and sequential IP with anti-FLAG and anti-HA antibodies. Each TRPV4 monomer contains 45 lysines, most of which reside in the cytosolic intracellular domains: 10 in the N-terminal IDR, 13 in the ARD, and 5 in the C-terminal IDR (hereafter CTD) (Fig. 2*B*, *black vertical lines*). Seventeen lysines are distributed throughout the transmembrane regions and the TRP box (Fig. 2*B*, *black vertical lines*). MS analyses of ubiquitinated TRPV4 derived from eight distinct transfections identified 15 ubiquitinated lysine residues (Table 1 and Fig. 2*B*, *green circles*): five localized to the N-terminal IDR (Lys 70, 77, 101, 130, and 136), six to the ARD (Lys 192, 197, 340, 344, 352, and 382), and three to the CTD (Lys 766, 801, and 834). A representative mass spectrum for lysine 130 is shown (Fig. 2*C*) with all mass spectra and ion tables available in the Appendix. No ubiquitinated lysines were identified in the transmembrane region or extracellular domains. Interestingly, Lys 192 and 197 form part of an ATP-binding pocket within the TRPV4 ARD (52, 53) (Fig. S1*D*). Ubiquitinated lysine residues were more frequently and consistently observed in the unstructured N-terminal and C-terminal IDRs than in the ARD (Table 1 and Table S1). The ubiquitinated lysine residues in the N-terminal IDR are distributed throughout the domain including sites within or near the two known functional domains, the PBD and PRR (Fig. 2*D*).

**Table 1:** Ubiquitinated lysine residues (in *bold*) in TRPV4 protein

TRPV4 functional domain	Lysine	Peptide	Percent of runs modified K was identified
IDR	70	[R].M**K**FQGAFR.[K]	37.5% (3/8)
	77	[R].**K**GVPNPIDLLESTLYESSVVPGPK.[K]	37.5% (3/8)
	101	[K].**K**APMDSLFDYGTYR.[H]	75% (6/8)
	130	[K].IIE**K**QPQSPK.[A]	87.5% (7/8)
	136	[K].QPQSP**K**APAPQPPPILK.[V]	62.5% (5/8)
ARD	192^a^	[R].LTDEEFREPSTG**K**TCLPK.[A]	25% (2/8)
	197^a^	[K].TCLP**K**ALLNLSNGR.[N]	25% (2/8)
	340	[R].ENT**K**FVTK.[M ]	25% (2/8)
	344^a^	[K].FVT**K**MYDLLLLK.[C]	25% (2/8)
	352^a^	[K].MYDLLLL**K**CAR.[L]	37.5% (3/8)
	382	[K].TG**K**IGIFQHIIR.[R]	12.5% (1/8)
	407	[K].F**K**DWAYGPVYSSLYDLSSLDTCGEEASVLEILVYNSK.[I]	12.5% (1/8)
CTD	766^a^	[R].SGEMVTVG**K**SSDGTPDR.[R]	100% (8/8)
		[R].SGEMVTVG**K**SSDGTPDRR.[W]	
	801	[R].VDEVNWSHWNQNLGIINEDPG**K**.[N]	25% (2/8)
		[R].VDEVNWSHWNQNLGIINEDPG**K**NETYQYYGFSHTVGR.[L]	
	834	[R].VVELN**K**NSNPDEVVVPLDSMGNPR.[C]	87.5% (7/8)
		[R].VVELN**K**.[N]	

a Also identified in a screen of the Ub proteome of mouse kidney (40).

### Blocking ubiquitination in the N-terminal IDR increases TRPV4 channel activity

To determine the functional effects of ubiquitination of specific TRPV4 protein domains, we genetically blocked ubiquitination by mutating select lysine residues to charge-preserving arginines (K > R) by site-directed mutagenesis. We generated four TRPV4 expression constructs: (i) All-K-IDR, in which all 10 lysine residues in the N-terminal IDR were mutated to arginine (listed in legend to Fig. S2*A*); (ii) 5K-IDR, in which five lysine residues in the N-terminal IDR selected based on their identification by MS analyses and proximity to the PBD and PRR were mutated (Lys 77, 101, 130, 136, and 147); (iii) a more limited K130, 136R mutant; and (iv) 5K-CTD, in which all five lysine residues within the CTD were targeted for mutagenesis (listed in the legend to Fig. S2*A*). Following expression in HEK293T cells, ubiquitination was diminished in the 5K-IDR and All-K-IDR mutants relative to the WT channel, although as expected, ubiquitination was still observed at the remaining lysine residues (Fig. S2*A* and Table 2). Culturing cells expressing the TRPV4 lysine mutants in the presence of the HC067 antagonist restored a normal level of ubiquitination of the 5K-IDR mutant but not of the All-K-IDR mutant (Fig. S2*B*).

**Table 2:** Ubiquitinated lysine residues (in *bold*) identified in TRPV4 K>R mutants

TRPV4 functional domain	Lysine	Peptide	Number of peptide-spectral matches (PSMs) in each run				
		TRPV4 K>R mutant →	WT	K130, 136R	5K-IDR	All-K IDR	5K-CTD
IDR	70	[R].M**K**FQGAFR.[K]	2				
	77	[R].**K**GVPNPIDLLESTLYESSVVPGPK.[K]	2	3			3
	100	[K].GVPNPIDLLESTLYESSVVPGP**K**K.[A]		2			1
	101	[K].**K**APMDSLFDYGTYR.[H]	10	5	1		4
		[K].GVPNPIDLLESTLYESSVVPGPK**K**.[A]	2				
	130	[K].IIE**K**QPQSPK.[A]	3				5
	136	[K].QPQSP**K**APAPQPPPILK.[V]	11				2
ARD	177	[R].GSTADLDGLLPFLLTH**K**K.[R]	5	2		2	2
	344	[K].FVT**K**MYDLLLLK.[C]	6	3	2		3
	379	[R].LFPDSNLEAVLNNDGLSPLMMAA**K**TGK.[I]					1
	382	[K].TG**K**IGIFQHIIR.[R]	2				
CTD	766	[R].SGEMVTVG**K**SSDGTPDR.[R]	5	2		1	
		[R].SGEMVTVG**K**SSDGTPDRR.[W]	9		1		
	801	[R].VDEVNWSHWNQNLGIINEDPG**K**NETYQYYGFSHTVGR.[L]	1	1		1	
	834	[R].VVELN**K**NSNPDEVVVPLDSMGNPR.[C]	8	3	2	1	

**Figure 3:**
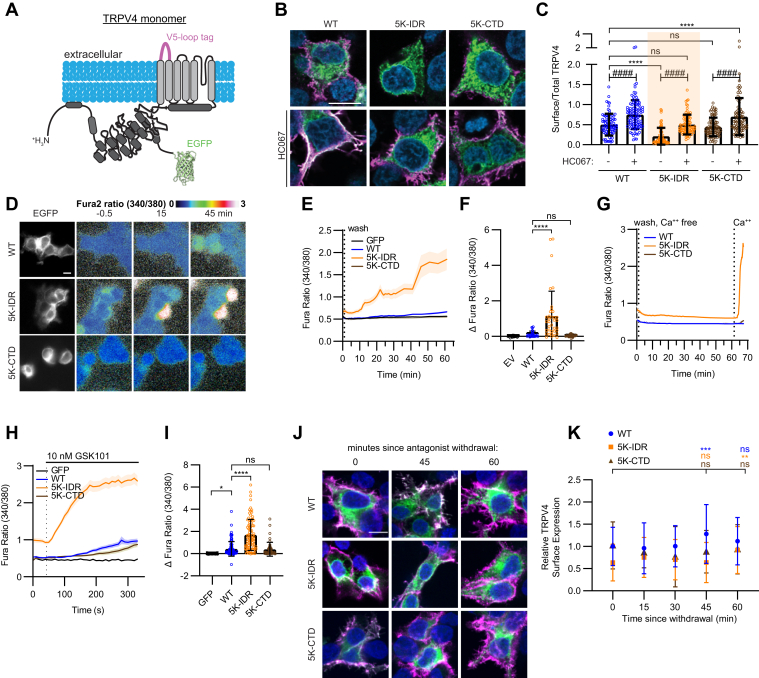
**Genetically blocking ubiquitination of the N-terminal IDR results in increased TRPV4 channel activity.***A*, schematic of a TRPV4 monomer indicating the location of the V5 tag in the first extracellular loop (*magenta*) and C-terminal EGFP tag (*green*) of the TRPV4-V5-EGFP construct. *B* and *C*, representative confocal images (*B*) and quantification (*C*) of unpermeabilized HEK293T cells transfected with TRPV4-V5-EGFP (WT, 5K-IDR, and 5K-CTD), showing cell surface (*magenta*; anti-V5 antibody) and total (*green*; EGFP tag) TRPV4 as well as nuclei (*blue*, DAPI) in the absence or the presence of HC067 (0.5 μM). The scale bar represents 10 μm. n = 69 to 90 cells/condition representing three coverslips from three independent transfections. Data are presented as means ± SD. ∗∗∗∗*p* < 0.0001 (one-way ANOVA with Dunnett’s post hoc test), ####*p* < 0.0001 (two-way ANOVA). *D*, Fura-2 ratiometric calcium imaging of HEK293T cells transfected with TRPV4-V5-EGFP constructs and cultured in HC067 (0.5 μM) for 24 h prior to antagonist washout at time 0. EGFP-positive cells for each condition are shown at *left*, and Fura-2 ratio images (warmer colors represent more intracellular calcium) prior to and following antagonist withdrawal are shown at *right*. The scale bar represents 10 μm. *E*, change in Fura-2 ratio (340/380) over the time course of antagonist withdrawal. The *vertical dotted line* indicates the time point at which HC067 was removed. n = 24–36 cells/condition from three independent coverslips. *Solid lines* represent means, and shading SEM. *F*, quantification of the maximum change in intracellular calcium from baseline to 60 min after HC067 washout for the experiments shown in *E*. n = 24 to 36 cells/condition from three independent transfections. Data are presented as means ± SD. ∗∗∗∗*p* < 0.0001 (one-way ANOVA with Dunnett’s post hoc test). *G*, calcium imaging of transfected HEK293T cells cultured in HC067 (0.5 μM) for 24 h prior to antagonist washout at time 0 and immediate switching to calcium-free buffer (*vertical dotted line* at *left*). Calcium was then reintroduced to the imaging buffer after 1 h (*vertical dotted line* at *right*). n = 31–38 cells/condition from two independent coverslips. Data are presented as means ± SEM. *H*, calcium imaging of transfected HEK293T cells cultured in HC067 (0.5 μM) for 24 h prior to antagonist washout (*vertical dotted line*) and application of the TRPV4-specific agonist GSK101 (10 nM; *horizontal black line*). *Solid lines* represent means, and *shading* represents SEM. *I*, quantification of the maximum change in intracellular calcium following GSK101 application for the experiments shown in *H*. n = 50 to 103 cells/condition from three independent coverslips. Data are presented as means ± SD. ∗*p* = 0.0455, ∗∗∗∗*p* < 0.0001 (one-way ANOVA with Dunnett’s post hoc test). *J* and *K*, representative confocal images (*J*) and quantification (*K*) of TRPV4 localization prior to and following HC067 removal. Cell surface and total TRPV4 were detected as in *B*. n = 29 to 135 cells/condition from two to three independent coverslips. Data are presented as means ± SD. ∗∗∗*p* = 0.0004, ∗∗*p* = 0.0023 (two-way ANOVA with Dunnett’s post hoc test). The scale bar in *J* represents 10 μm. CTD, C-terminal IDR; DAPI, 4′,6-diamidino-2-phenylindole; EGFP, enhanced GFP; GSK101, GSK1016790A; HC067, TRPV4-specific antagonist HC067047; HEK293T, human embryonic kidney 293T cell line; IDR, intrinsically disordered region; TRPV4, transient receptor potential vanilloid 4.

To assess the subcellular distribution and channel activity of the TRPV4 K>R mutants, we generated constructs containing a V5 tag in the first extracellular loop and an enhanced GFP (EGFP) tag at the C terminus (TRPV4-V5-EGFP) (Fig. 3*A*). Surface TRPV4 was visualized by incubating nonpermeabilized cells with an anti-V5 antibody, and total TRPV4 was visualized using the EGFP tag. Fluorescence intensity was measured using confocal microscopy, and the ratio of surface to total TRPV4 was determined. Experiments were performed in the presence or the absence of the antagonist HC067, both to assess effects of channel activity on cell surface expression of the mutants and to determine whether observed changes in intracellular calcium resulted from TRPV4 activation. In the absence of HC067, the 5K-IDR mutant showed reduced surface expression compared with the WT channel (Fig. 3, *B* and *C*). Strikingly, however, 5K-IDR-expressing cells displayed elevated intracellular calcium levels, as measured by Fura-2 ratiometric calcium imaging (Fig. S2*C*), suggesting that this mutant exhibits increased channel activity. Culturing of 5K-IDR-transfected cells in the presence of HC067 rescued surface localization of the mutant channel and reduced intracellular calcium levels (Figs. 3*C* and S2*C*). Together, these data suggest that the 5K-IDR mutant exhibits increased channel activity at the cell surface. The more limited IDR mutant targeting lysines 130 and 136 (K130, 136R) behaved similarly to WT in its surface localization and channel activity in the absence or the presence of HC067 (Fig. S2, *C*–*E*). 5K-CTD-expressing cells also exhibited levels of TRPV4 surface expression and intracellular calcium comparable to cells expressing the WT channel (Figs. 3*C* and S2*C*).

To further explore the activity of the 5K-IDR TRPV4 mutant, we used an antagonist withdrawal paradigm in which transfected HEK293T cells were cultured in HC067 for 24 h, and calcium imaging was performed after removing the antagonist. While cells expressing WT, 5K-CTD, or K130, 136R TRPV4 proteins showed minimal changes in intracellular calcium levels over 60 min after HC067 removal (Figs. 3, *D* and *E* and S2*F*), cells transfected with 5K-IDR TRPV4 showed marked increases in intracellular calcium levels over time (Fig. 3, *E* and *F*). Performing the antagonist withdrawal experiment in calcium-free media did not result in increased intracellular calcium levels of 5K-IDR TRPV4-expressing cells until extracellular calcium was provided (Fig. 3*G*). These data suggest that calcium flux through the TRPV4 channel at the cell surface is required for intracellular calcium accumulation. We also assessed the activity of the 5K-IDR TRPV4 mutant in cells treated with the TRPV4-specific agonist GSK1016790A (GSK101) following HC067 washout (Fig. 3*H*). 5K-IDR-expressing cells showed a 300% greater response to 10 nM GSK101 compared with WT-CTD-expressing cells or 5K-CTD-expressing cells (Fig. 3*I*). Examination of the surface abundance of the 5K-IDR mutant during the period of increased calcium influx following antagonist withdrawal (Fig. 3*E*) revealed little change in 5K-IDR TRPV4 surface abundance and certainly not an accumulation of the 5K-IDR mutant at the cell surface relative to WT (Fig. 3, *J* and *K*). Together, these data demonstrate that blocking ubiquitination at specific lysines in the N-terminal IDR, but not the CTD, results in increased TRPV4 channel activity with little change in surface abundance.

### The TRPV4 IDR can be ubiquitinated by the NEDD4 family of E3 Ub ligases

**Figure 4:**
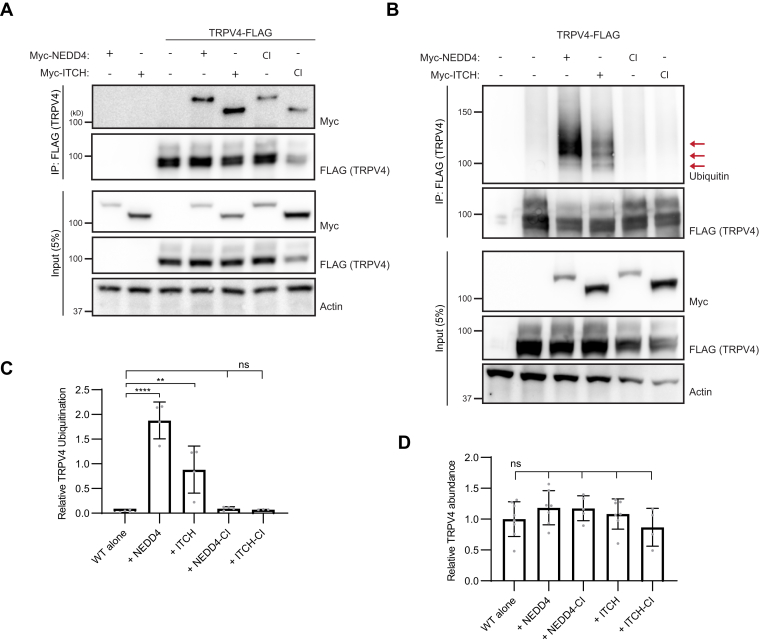
**TRPV4 is ubiquitinated by NEDD4 family E3 ubiquitin ligases.***A*, coimmunoprecipitation of TRPV4-FLAG with Myc-NEDD4 and Myc-ITCH as well as their catalytically inactive (CI) mutants, in transiently transfected HEK293T cells. *B*, TRPV4 ubiquitination (*red arrows*) in transfected HEK293T cells with coexpression of NEDD4 or ITCH or their CI mutants. *C*, densitometry-based quantification of *B*. n = four transfections/condition. Data are presented as means ± SD. ∗∗∗∗*p* < 0.0001, ∗∗*p* = 0.003 (one-way ANOVA with Dunnett’s post hoc test). *D*, quantification of TRPV4 abundance in the presence or the absence of E3 ligase coexpression. n = 4 to 8 independent experiments. Data are presented as means ± SD. ns (one-way ANOVA Dunnett’s multiple comparison test). HEK293T, human embryonic kidney 293T cell line; ns, not significant; TRPV4, transient receptor potential vanilloid 4; TRPV4-FLAG, FLAG-tagged TRPV4.

Given our results demonstrating that blocking ubiquitination of the TRPV4 N-terminal IDR increased channel activity, we next sought to determine the functional consequences of enhancing ubiquitination. The NEDD4 family of E3 Ub ligases is well studied in the context of ion channel ubiquitination (24), and NEDD4 in particular is known to function at the plasma membrane because of its C2 lipid-binding domain (54, 55). In HEK293T cells, we observed that TRPV4 coimmunoprecipitated with two NEDD4 family members, NEDD4 and ITCH, as well as their catalytically inactive mutants (NEDD4-CI and ITCH-CI) (56) (Fig. 4*A*). These data confirm a previous report demonstrating that ITCH interacts with TRPV4 (50). These interactions were specific for TRPV4, as neither NEDD4 nor ITCH modify the ubiquitination of TRPV1 (Fig. S3*A*). As expected, only coexpression of catalytically active NEDD4 or ITCH resulted in increased ubiquitination of TRPV4, with a 110% increase in TRPV4 ubiquitination observed with NEDD4 compared with ITCH (Fig. 4, *B* and *C*). Neither NEDD4 nor ITCH coexpression reduced total TRPV4 levels at 24 h post-transfection, suggesting that ubiquitination is not a degradative signal for TRPV4 (Fig. 4*D*).

To determine which specific TRPV4 lysine residues become ubiquitinated in the presence of NEDD4, Myc-tagged NEDD4 was coexpressed with TRPV4-FLAG and HA-Ub in HEK293T cells, followed by double IP and MS analysis as outlined in Figure 2*A*. In the presence of only endogenous Ub ligases, five lysine residues were observed to be ubiquitinated in the N-terminal IDR and three in the CTD. In the presence of NEDD4, four of the five lysine residues in the N-terminal IDR and two of the three lysine residues in the CTD were ubiquitinated (Tables 1 and 3). However, one additional lysine residue (Lys 147) was found to be ubiquitinated in the N-terminal IDR solely in the presence of NEDD4 (Table 3).

**Table 3:** Ubiquitinated lysine residues (in *bold*) identified in TRPV4 without or with NEDD4 coexpression

TRPV4 functional domain	Lysine	Peptide		
		Experimental conditions →	WT alone	+ NEDD4
IDR	70	[R].M**K**FQGAFR.[K]	100% (3/3)	0% (0/2)
	77	[R].**K**GVPNPIDLLESTLYESSVVPGPK.[K]	100% (3/3)	100% (2/2)
	101	[K].**K**APMDSLFDYGTYR.[H]	100% (3/3)	100% (2/2)
		[R].KGVPNPIDLLESTLYESSVVPGPK**K**.[A]		
	130	[K].IIE**K**QPQSPK.[A]	100% (3/3)	50% (1/2)
	136	[K].QPQSP**K**APAPQPPPILK.[V]	100% (3/3)	100% (2/2)
	147	[KR].APAPQPPPIL**K**VFNRPILFDIVSR.[G]	0% (0/3)	100% (2/2)
ARD	178	[R].GSTADLDGLLPFLLTHK**K**.[R]	33% (1/3)	100% (2/2)
	344	[K].FVT**K**MYDLLLLK.[C]	100% (3/3)	0% (0/2)
	379	[R].LFPDSNLEAVLNNDGLSPLMMAA**K**TGK.[I]	66% (2/3)	0% (0/2)
CTD	766	[R].SGEMVTVG**K**SSDGTPDR.[R]	100% (3/3)	0% (0/2)
		[R].SGEMVTVG**K**SSDGTPDRR.[W]		
	801	[R].VDEVNWSHWNQNLGIINEDPG**K**NETYQYYGFSHTVGR.[L]	0% (0/3)	100% (2/2)
	834	[R].VVELN**K**NSNPDEVVVPLDSMGNPR.[C]	100% (3/3)	100% (2/2)

NEDD4 and ITCH sometimes act directly on protein targets but in other cases require coadaptors to ubiquitinate a substrate (57, 58, 59, 60). To test whether their ubiquitination of TRPV4 is direct, we performed cell-free Ub assays combining recombinant NEDD4 or ITCH with either recombinant TRPV4 full N terminus (IDR + ARD) or ARD fragments (Fig. S3*B*). Autoubiquitination of each E3 ligase provided evidence of enzymatic activity (Fig. S3, *C*–*E*, *red arrowheads*). NEDD4 did not induce TRPV4 ubiquitination in this system, suggesting requirement of a coadaptor protein (Fig. S3*C*), whereas ITCH induced ubiquitination of the full TRPV4 N terminus but not of the ARD fragment or of two additional N-terminal domain fragments: IDR and ARD + PRR (Fig. S3, *D* and *E*). MS analysis indicated that four of the IDR residues identified from the whole cell analysis (Fig. 2) can be ubiquitinated by ITCH: Lys 77, 101, 130, and 136. The additional IDR lysine residue identified as a site of ubiquitination in cells expressing NEDD4 (Lys 147) was also ubiquitinated in the presence of ITCH in this cell-free assay (Fig. S3*B*, *green circles*, Table S2). Together, these data demonstrate that NEDD4 and ITCH primarily ubiquitinate TRPV4 at the same N-terminal lysine residues, and that these residues exhibit extensive overlap with those identified in cell-based assays performed in the absence of E3 ligase overexpression.

### Enhancing TRPV4 IDR ubiquitination dampens stimulated channel responses

**Figure 5:**
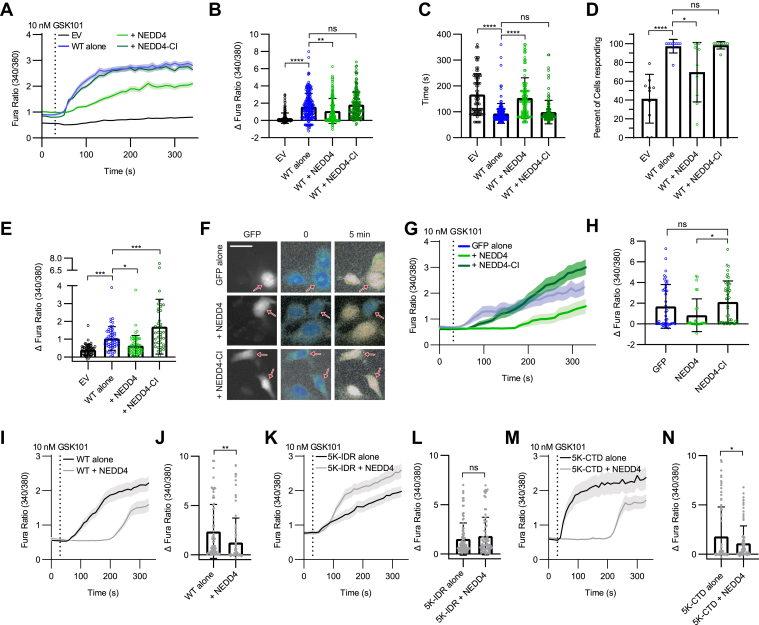
**Enhancing N-terminal IDR ubiquitination with NEDD4 overexpression reduces stimulated TRPV4 channel activity.***A*, calcium imaging of HEK293T cells expressing either empty vector (EV), WT TRPV4 alone, or with either NEDD4 or NEDD4-CI, showing responses to stimulation with GSK101 (10 nM; applied at the *vertical dotted line*). n = 153–189 cells/condition representing 9–10 individual coverslips. *Solid lines* represent means, and shading represents SEM. *B*, quantification of the maximum change in intracellular calcium with GSK101 application for the experiments shown in *A*. n = 153 to 189 cells/condition. Data are presented as means ± SD. ∗∗∗∗*p* < 0.0001, ∗∗*p* = 0.0022 (one-way ANOVA with Dunnett’s post hoc test). *C*, latency of the calcium response to GSK101, as defined by the first time point at which Fura-2 ratio is greater than 5 SD above baseline levels. n = 76 to 186 cells/condition. Data are presented as means ± SD. ∗∗∗∗*p* < 0.0001 (one-way ANOVA with Dunnett’s post hoc test). *D*, percent of cells responding to GSK101 application with an increase in Fura-2 ratio greater than 5 SD above baseline levels. n = 9 to 10 coverslips/condition. Data are presented as means ± SD. ∗∗∗∗*p* < 0.0001, ∗*p* = 0.0161 (one-way ANOVA). *E*, quantification of the maximum change in intracellular calcium levels following stimulation with hypotonic saline. Data are presented as means ± SD. n = 42–56 cells/condition from three independent coverslips. ∗∗∗*p* = 0.0004, ∗*p* = 0.0315, ∗∗∗*p* = 0.0006. *F*, calcium imaging of 50B11 cells cotransfected with GFP and either NEDD4 or NEDD4-CI. GFP-positive cells for each condition are shown at *left* and indicated by *red arrows*. Fura-2 ratio images at time 0 and 5 min after GSK101 (10 nM) stimulation are shown at *right*. The scale bar represents 10 μm. *G*, time course of 50B11 calcium responses following GSK101 application (10 nM; applied at *vertical dotted line*). *Solid lines* represent means, and shading represents SEM. *H*, quantification of the change in intracellular calcium following GSK101 application for the experiments shown in *G*. n = 28 to 46 cells/condition from four coverslips representing four independent transfections. Data are presented as means ± SD. ∗*p* = 0.0137 (one-way ANOVA with Dunnett’s post hoc test). *I*–*N*, WT, 5K-IDR, or 5K-CTD TRPV4 was transfected into HEK293T cells without or with NEDD4 coexpression. Cells were cultured in HC067 (0.5 μM) for 24 h prior to antagonist washout and immediate switching to media containing GSK101 (10 nM; *vertical dotted line*). *I* and *J*, show transfection of WT TRPV4. n = 72 to 92 cells/condition, ∗∗*p* = 0.0035 (two-tailed unpaired *t* test). *K* and *L*, show transfection of 5K-IDR TRPV4. n = 74 to 101 cells/condition, ns (two-tailed unpaired *t* test). *M* and *N*, show 5K-CTD TRPV4-transfected cells. n = 84 to 85 cells/condition, ∗*p* = 0.0311 (two-tailed, unpaired *t* test). All histograms are presented as means ± SD. CI, catalytically inactive; CTD, C-terminal IDR; GSK101, GSK1016790A; HC067, TRPV4-specific antagonist HC067047; HEK293T, human embryonic kidney 293T cell line; IDR, intrinsically disordered region; ns, not significant; TRPV4, transient receptor potential vanilloid 4.

We next sought to determine the functional consequences of increased TRPV4 ubiquitination by NEDD4. HEK293T cells expressing WT TRPV4 alone, WT TRPV4 together with NEDD4, or WT TRPV4 together with NEDD4-CI for 24 h were treated with the TRPV4 agonist GSK101 (10 nM). Some cells expressing TRPV4 and NEDD4 showed an elevated baseline calcium prior to GSK101 application (Fig. S4*A*, *left of dotted line*). To ensure assessment of a healthy population of cells, we analyzed only those cells that did not exceed two standard deviations above WT TRPV4 baseline intracellular calcium levels. This analysis resulted in the exclusion of 11 (5.5%) cells from WT TRPV4 alone, 24 (13.6%) cells from WT TRPV4 + NEDD4, and 12 (6.2%) cells from WT TRPV4 + NEDD4-CI conditions (Fig. 5*A*). Coexpression of TRPV4 with NEDD4, but not NEDD4-CI, reduced the maximal intracellular calcium level observed following GSK101 application (Fig. 5*B*), delayed the time to reach this maximal response (Fig. 5*C*), and reduced the percent of cells responding to the TRPV4 agonist (Fig. 5*D*). NEDD4 coexpression also dampened and delayed calcium responses of cells treated with hypotonic saline (HTS, 37.5 mM NaCl), a well-characterized nonspecific TRPV4 activator (Figs. 5*E*, S4, *B* and *C*) (61). Increased calcium influx after HTS observed in cells expressing NEDD4-CI might result from NEDD4-CI binding and stabilizing other plasmalemma ion channels that respond to hypotonic solutions. Furthermore, when NEDD4 was overexpressed in 50B11 cells expressing endogenous TRPV4, we observed that NEDD4 but not NEDD4-CI reduced maximal intracellular calcium levels in response to GSK101 treatment (Fig. 5, *F*–*H*).

We next assessed whether the ability of NEDD4 to dampen GSK101-induced responses in HEK293T cells required the presence of specific lysine residues in the N-terminal IDR or CTD. We confirmed that the 5K-IDR and 5K-CTD lysine mutations did not interrupt binding of NEDD4 to TRPV4 (Fig. S4*D*) and then examined calcium responses of WT, 5K-IDR, and 5K-CTD TRPV4 to 10 nM GSK101 after antagonist washout. As expected, NEDD4 reduced WT TRPV4 responses to agonist stimulation (Fig. 5, *I* and *J* and S4*E*). Strikingly, NEDD4 did not suppress the response of 5K-IDR TRPV4 (Fig. 5, *K* and *L* and S4*F*) but did reduce 5K-CTD TRPV4 responses (Fig. 5, *M* and *N* and S4*G*) in a similar fashion to WT TRPV4. These findings indicate that NEDD4 suppressed WT TRPV4 channel activity through ubiquitination of the TRPV4 N-terminal IDR.

### Increased TRPV4 IDR ubiquitination does not reduce TRPV4 cell surface abundance

**Figure 6:**
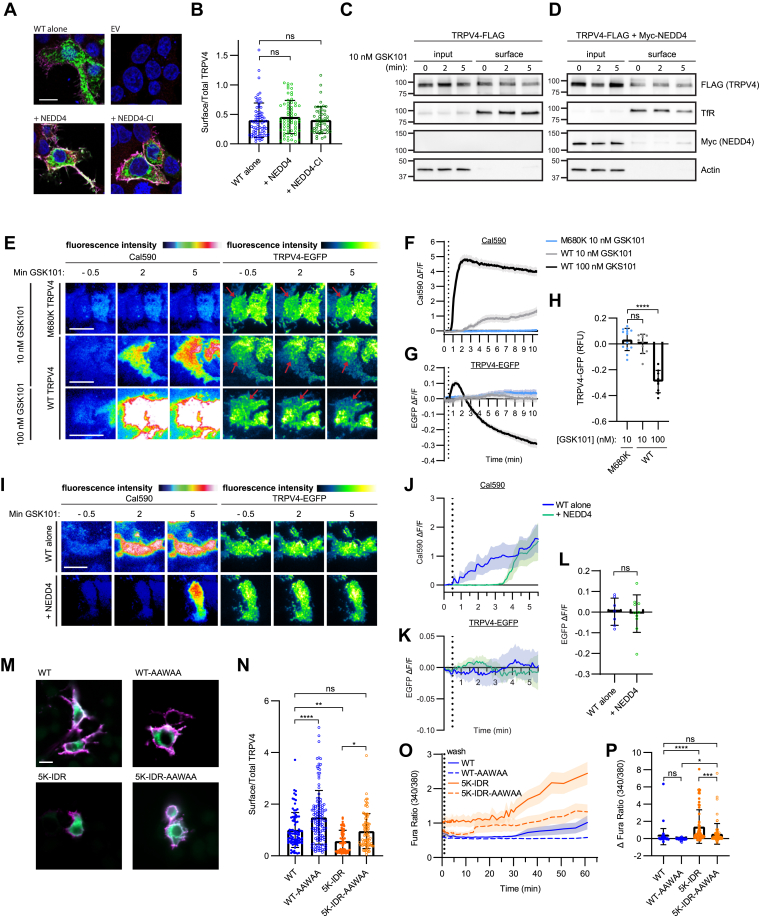
**NEDD4 overexpression does not reduce TRPV4 surface localization.***A*, representative confocal images of HEK293T cells cotransfected with TRPV4-V5-EGFP and either Myc-NEDD4 or Myc-NEDD4-CI. Unpermeabilized cells were first stained with V5 antibody to identify surface-localized TRPV4 (*magenta*) and then fixed and permeabilized to stain for intracellular Myc-tagged NEDD4 (*red*). The scale bar represents 10 μm. *B*, quantification of relative surface fluorescence of TRPV4 from experiments shown in *A*. n = 51 to 86 cells/condition representing three independent experiments. Data are presented as means ± SD (one-way ANOVA with Dunnett’s post hoc test). *C* and *D*, Western blot analysis of TRPV4 cell surface biotinylation assays in transfected HEK293T cells showing changes in TRPV4 surface localization after GSK101 (10 nM) stimulation in the absence (*C*) or presence (*D*) of NEDD4 coexpression. *E*–*H*, TIRF imaging of HEK293T cells transfected with either WT or M680K (pore-inactive) TRPV4-EGFP and stimulated by GSK101 (10–100 nM) application. Cells were loaded with the calcium indicator Cal590 prior to imaging to allow for simultaneous assessments of TRPV4 surface localization and intracellular calcium levels. Representative TIRF images prior to and following GSK101 application are shown in *E*; warmer colors represent increased levels of intracellular calcium (Cal590) or increased TRPV4 localization within the TIRF evanescent (TRPV4-EGFP). The scale bar represents 10 μm. Quantification of the fold changes (ΔF/F) in intracellular calcium and TRPV4 surface localization following GSK101 application are shown in *F* and *G*, respectively. *Solid lines* represent means, and shading represent SEM. n = 9–13 cells/condition from four independent experiments. *H*, quantification of surface TRPV4 fluorescence in the TIRF evanescent field 10 min after treatment with GSK101. Data are represented as means ± SD. n = 9 to 13 cells/condition from four independent experiments. ∗∗∗∗*p* < 0.0001 (one-way ANOVA with Dunnett’s multiple comparison test). *I*–*L*, TIRF imaging of HEK293T cells transfected with WT TRPV4-EGFP alone or with NEDD4 and stimulated by GSK101 (10 nM) application. Representative TIRF images prior to and following GSK101 application are shown in *I*. The scale bar represents 10 μm. Quantification of the fold changes (ΔF/F) in intracellular calcium and TRPV4 surface localization following GSK101 treatment are shown in *J* and *K*, respectively. *Solid lines* represent means, and shading represent SEM. n = 7 cells/condition from four independent experiments. *L*, quantification of the changes in TRPV4-EGFP TIRF signal for the experiments shown in *K*, examining the effects of NEDD4 coexpression on WT TRPV4 surface localization in response to 10 nM GSK101 treatment. Data are presented as means ± SD. n = 7–8 cells/condition from four independent experiments. Unpaired two-tailed *t* testK-. *M*, representative images of HEK293T cells expressing either WT or 5IDR TRPV4 and their AAWAA counterparts. Unpermeabilized cells were stained with V5 antibody to identify surface-localized TRPV4 (*magenta*), and total TRPV4 was visualized by the EGFP tag (*green*). The scale bar represents 10 μm. *N*, quantification of relative surface fluorescence of TRPV4 from the experiments shown in *M*. n = 51 to 76 cells/condition representing two independent experiments. Data are presented as means ± SD. ∗*p* = 0.0106, ∗∗*p* = 0.0032, ∗∗∗∗*p* < 0.0001 (one-way ANOVA with Sidak’s multiple comparison test). *O*, change in Fura-2 ratio (340/380) over the time course of antagonist withdrawal. The *vertical dotted line* indicates the time point at which HC067 was removed. n = 51–76 cells/condition from three independent coverslips. *Solid lines* represent means, and shading represents SEM. *P*, quantification of the maximum change in intracellular calcium from baseline to 60 min after HC067 removal for the experiments shown in *O*. n = 51 to 76 cells/condition from four independent transfections. Data are presented as means ± SD. ∗∗∗∗*p* < 0.0001, ∗∗∗*p* = 0.0006, ∗*p* = 0.0483 (one-way ANOVA with Sidak’s post hoc test). EGFP, enhanced GFP; GSK101, GSK1016790A; HC067, TRPV4-specific antagonist HC067047; HEK293T, human embryonic kidney 293T cell line; TIRF, total internal reflection fluorescence; TRPV4, transient receptor potential vanilloid 4.

Canonically, ubiquitination of plasmalemmal ion channels reduces channel activity by stimulating channel endocytosis and removal from the plasma membrane (62). Conversely, blocking ubiquitination results in channel accumulation at the plasma membrane. However, our data from the 5K-IDR mutant suggest a gain-of-channel function without a change in cell surface expression (Fig. 3). We thus employed multiple methods to assess the effects of NEDD4 expression on TRPV4 cell surface localization. First, surface to total TRPV4 ratios were determined in HEK293T cells transfected with TRPV4-V5-EGFP alone or together with either NEDD4 or NEDD4-CI. Despite exhibiting increased ubiquitination with expression of NEDD4 (Fig. 4*B*), TRPV4 surface levels were not reduced by coexpression of NEDD4 or NEDD4-CI (Fig. 6, *A* and *B*). Comparable results were obtained using flow cytometry to assess TRPV4 surface localization in motor neuron–like 1 (MN-1) cells expressing TRPV4-V5-FLAG alone or with NEDD4 (Fig. S5*A*). We next used cell surface biotinylation of transfected HEK293T cells to assess TRPV4 expression at the plasma membrane at baseline and following stimulation with GSK101. In this assay, coexpression of NEDD4 did not alter surface TRPV4 abundance at baseline or in response to TRPV4 channel stimulation (Figs. 6, *C* and *D*, S5, *B*–*E*). Together, these approaches suggest that NEDD4-mediated ubiquitination of TRPV4 does not significantly alter TRPV4 surface expression.

To assess cell surface localization in living cells we utilized total internal reflection fluorescence (TIRF) microscopy to measure TRPV4 surface fluorescence and intracellular calcium levels simultaneously using EGFP-tagged TRPV4 and the calcium indicator Cal590. Using TIRF microscopy, it has been previously reported that supramaximal doses of GSK101 (100 nM) cause rapid TRPV4 internalization from the plasma membrane in stably transfected HEK293 cells (63). We therefore first assessed TRPV4 dynamics and calcium levels in cells expressing WT TRPV4 treated with GSK101 at a dose of 10 nM (used throughout this study) or 100 nM. TRPV4 containing the M680K mutation that blocks the channel pore (64) was used as a negative control. Application of 10 nM GSK101 resulted in increased intracellular calcium concentrations but no loss of TRPV4 fluorescence from the TIRF evanescent field (Fig. 6, *E*–*H*, *gray lines*). In contrast, treatment with 100 nM GSK101 caused a large increase in intracellular calcium and a rapid loss of TRPV4 fluorescence from the TIRF evanescent field (Fig. 6, *E*–*H*, *black lines*). These data indicate that channel activation is not associated with internalization at low levels of stimulation, whereas supramaximal doses of GSK101 lead to rapid and robust TRPV4 internalization. We next assessed the effect of NEDD4 coexpression on TRPV4 TIRF fluorescence after treatment with 10 nM GSK101 (Fig. 6, *I*–*L*). NEDD4 delayed the onset of increased calcium levels following agonist application (Figs. 6, *I* and *J*, S5*F*), without an associated reduction in TRPV4 fluorescence from the TIRF evanescent field, indicating that the reduced calcium influx was not caused by decreased TRPV4 surface localization (Fig. 6, *K* and *L*).

Given that ubiquitination of TRPV4 did not alter cell surface expression, we investigated whether known N-terminal protein–protein and protein–lipid interactions were affected by blocking ubiquitination. Interactions of the TRPV4 N-terminal domain with intracellular proteins such as RhoA and protein kinase C and casein kinase substrate in neurons 1–3 (PACSIN1–3) can regulate TRPV4 channel activity (Fig. S5*G*) without altering channel surface expression (29, 32, 35, 65). Interactions with PIP_2_ influence TRPV4 channel activity as well (36, 65, 66). Co-IP studies of PACSIN1 and TRPV4 showed reduced binding of PACSIN1 to 5K-IDR but not 5K-CTD TRPV4 (Fig. S5, *H* and *I*). However, mutation of TRPV4 residues K130 and K136 alone, which are part of the PACSIN SH3 domain–binding site, also reduced PACSIN1 binding (Fig. S5, *H* and *I*). As the K130, 136R mutations do not result in increased TRPV4 channel activity (Fig. S2*F*), these results indicate that the gain of function of the 5K-IDR mutant is not solely explained by disrupted PACSIN1 interaction. In addition, co-IP studies showed no differences in RhoA interaction between WT, 5K-IDR, and 5K-CTD TRPV4 (Fig. S5*J*). To assess whether altered lipid binding might contribute to increased channel activity of 5K-IDR TRPV4, we introduced neutralizing mutations within the positively charged residues of the PBD (^121^KRWKR^125^ to ^121^AAWAA^125^) in WT and 5K-IDR TRPV4 to disrupt interaction between the TRPV4 IDR and PIP_2_ (36, 65). The 5K-IDR-AAWAA TRPV4 mutant was expressed at the cell surface at similar levels to WT TRPV4 (Fig. 6, *M* and *N*) but partially rescued the gain of function of 5K-IDR TRPV4 in the antagonist withdrawal paradigm (Fig. 6, *O* and *P*). These data indicate that blocking PIP_2_ binding can partially rescue the increased channel activity associated with decreased N-terminal ubiquitination and suggest that ubiquitination may regulate channel activity in part through modulation of lipid binding.

### Neuropathy-causing mutations exhibit reduced ubiquitination correlating with increased channel activity

**Figure 7:**
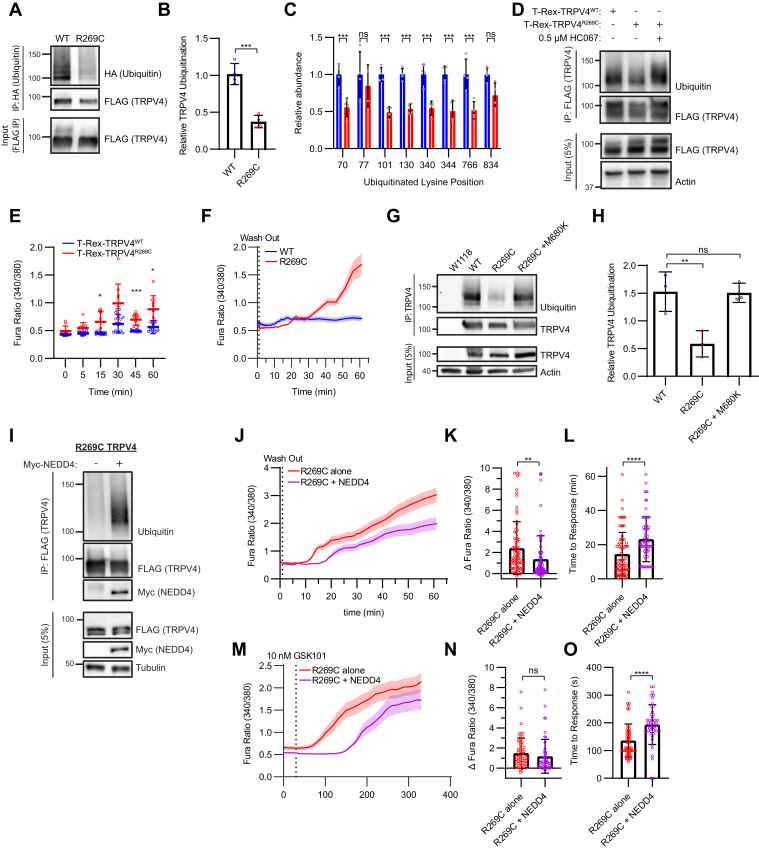
**Neuropathy-causing TRPV4 mutants exhibit reduced ubiquitination and a gain-of-channel function, which can both be partially rescued by NEDD4 overexpression.***A* and *B*, representative Western blot analysis (*A*) and quantification (*B*) demonstrating reduced ubiquitination of R269C TRPV4 relative to WT channel in transfected HEK293T cells. Ubiquitinated TRPV4 was isolated as outlined in Figure 2*A*. n = 4 experiments/condition. Data are presented as means ± SD. ∗∗*p* = 0.0003 (unpaired two-tailed *t* test). *C*, tandem mass tags (TMTs) were used to quantify relative ubiquitination of specific lysine residues on either WT or R269C TRPV4 in transfected HEK293T cells. n = 4 biological replicates/condition. Data are represented as means ± SD. ∗∗∗*p* < 0.0009 (two-way ANOVA with Šídák's multiple comparisons test). *D*, cell-based ubiquitination assay utilizing T-Rex-TRPV4 cells in which TRPV4 expression was induced over 18 h, demonstrating increased ubiquitination of the R269C mutant in the presence of HC067 (0.5 μM). *E* and *F*, intracellular calcium levels of induced T-Rex-TRPV4 cells cultured in HC067 (0.5 μM) for 18 h prior to antagonist washout at time 0 (*vertical dotted line* in *F*). n = 10 independent coverslips per time point. Data are presented as means ± SD. ∗*p* < 0.05, ∗∗∗*p* = 0.0007 (two-way ANOVA with Šídák's multiple comparisons test). *G*, *In vivo* ubiquitination assay demonstrating reduced ubiquitination of R269C TRPV4 in transgenic *Drosophila.* Lysates from nontransgenic *Drosophila* (*W1118*) were processed in parallel as controls. *H*, densitometry-based quantification of *G*. n = 3 independent experiments. Data are presented as means ± SD. ∗∗*p* = 0.0089 (one-way ANOVA with Dunnett’s post hoc test). *I*, cell-based ubiquitination assay performed using transfected HEK293T cells, demonstrating that NEDD4 coexpression increases ubiquitination of R269C TRPV4. *J*, intracellular calcium levels measured for 1 h following withdrawal of HC067 (0.5 μM; *vertical dotted line*) from R269C TRPV4-expressing cells transfected with or without NEDD4. n = 76 cells/condition representing three independent experiments. *Solid lines* represent means, and shading represents SEM. *K*, quantification of the increase in intracellular calcium from baseline to 60 min after HC067 removal for the experiments shown in *J*. Data are presented as means ± SD. ∗∗*p* = 0.0069 (unpaired two-tailed *t* test). *L*, time to response quantified from *J*. n = 71 to 74 cells/condition. ∗∗∗∗*p* < 0.0001 (unpaired two-tailed *t* test). *M*, calcium imaging of HEK293T cells expressing R269C TRPV4 without or with NEDD4 coexpression, showing responses to application of GSK101 (10 nM) immediately following HC067 removal (*vertical dotted line*). n = 51–57 cells/condition from three independent transfections. *Solid lines* represent means, and shading represents SEM. *N* and *O*, quantification of the increase in intracellular calcium from baseline to 5 min after GSK101 treatment (*N*) and time to response (*O*) for the experiment shown in *M*. n = 46 to 52 cells/condition. Data are presented as means ± SD. ∗∗∗∗*p* < 0.0001 (unpaired two-tailed *t* test). GSK101, GSK1016790A; HC067, TRPV4-specific antagonist HC067047; HEK293T, human embryonic kidney 293T cell line; TRPV4, transient receptor potential vanilloid 4.

We and others have previously shown that neuropathy-causing mutations in TRPV4 result in increased basal and stimulated channel activity (Fig. S6*A*) (29, 30, 31). While neuropathy-causing mutations are predominantly localized to one face of the ARD, interplay between the N-terminal IDR and the ARD has been proposed as a potential mechanism of TRPV4 channel modulation (31, 33). In fact, it is still unknown how neuropathy-causing mutations result in increased channel activity. To determine whether neuropathy-causing mutations might impact ubiquitination of the TRPV4 N-terminal IDR, we performed cell-based Ub assays examining transiently transfected HEK293T cells, as well as HEK293T cell lines that inducibly express either WT (T-Rex-TRPV4^WT^) or R269C neuropathy mutant (T-Rex-TRPV4^R269C^) FLAG-tagged TRPV4 (32). Utilizing both approaches, we observed reduced ubiquitination of R269C TRPV4 compared with WT (Figs. 7, *A* and *B*, S6*B*). The relative abundance of ubiquitinated peptides generated from WT and R269C TRPV4 was assayed by MS using isobaric labeling with tandem mass tags (TMTs) (Fig. 7*C*). Ubiquitination of R269C TRPV4 was reduced by approximately half at several lysine residues located in the N-terminal IDR, ARD, and CTD domains, indicating that R269C TRPV4 exhibits a broad reduction in ubiquitination, rather than a loss at specific sites (Fig. 7*C*).

To address whether neuropathy-causing TRPV4 mutants exhibit an intrinsic deficit in their capacity to be ubiquitinated, or whether reduced ubiquitination is a downstream consequence of elevations in intracellular calcium resulting from the gain-of-channel function, we assessed the effects of channel activity on TRPV4 ubiquitination. Culturing of T-Rex-TRPV4 cells in HC067 rescued T-Rex-TRPV4^R269C^ ubiquitination to T-Rex-TRPV4^WT^ ubiquitination levels (Fig. 7*D*) and equalized baseline intracellular calcium levels (Fig. 7*E*). Withdrawing the antagonist resulted in increased intracellular calcium levels in T-Rex-TRPV4^R269C^ cells compared with T-Rex-TRPV4^WT^ cells (Fig. 7*E*). Similar results were observed in transiently transfected HEK293T cells (Fig. 7*F*). In a *Drosophila* model of TRPV4 neuropathy (67), we also observed reduced ubiquitination of R269C TRPV4 as well as rescue of TRPV4 Ub levels in flies expressing R269C TRPV4 along with the pore-inactivating M680K mutation (Fig. 7, *G* and *H*). These data suggest that the reduced ubiquitination of neuropathy-causing TRPV4 mutants is downstream of their increased channel activity and not because of a structural inability of the protein to be ubiquitinated.

### Enhancing TRPV4 ubiquitination can partially rescue the gain-of-channel function characteristic of neuropathy-causing mutants

Having observed that increasing WT TRPV4 ubiquitination reduces stimulated calcium responses, we postulated that increasing the ubiquitination of neuropathy mutant TRPV4 might ameliorate its gain-of-channel function. We found that NEDD4 coimmunoprecipitates with R269C TRPV4 (Fig. 7*I*), and that NEDD4 expression both enhanced R269C TRPV4 ubiquitination and inhibited mutant channel activity upon antagonist withdrawal (Fig. 7, *I*–*L*). Furthermore, NEDD4 expression delayed calcium influx in R269C TRPV4-expressing cells stimulated with 10 nM GSK101 (Fig. 7, *M*–*O*). We also confirmed NEDD4-mediated suppression of neuropathy mutant channel activity by examining a second neuropathy-causing mutant (R237L TRPV4) in MN-1 cells. In these cells, NEDD4 coexpression reduced GSK101 responses in both WT and R237L neuropathy mutant-expressing cells (Fig. S6*C*). Together, these data further highlight the importance of N-terminal IDR ubiquitination in modulating TRPV4 channel activity and identify alterations of this pathway resulting from neuropathy-causing mutations that can be partially rescued by enhancing TRPV4 ubiquitination.

## Discussion

As the plasmalemmal cation channel TRPV4 is increasingly implicated in both normal and pathologic states in numerous tissues (3, 51, 68, 69, 70, 71, 72, 73, 74, 75, 76) and is activated by multiple physiological stimuli, it likely requires diverse regulatory mechanisms to be utilized in distinct cellular contexts (77). However, the molecular mechanisms regulating the TRPV4 channel under homeostatic and pathologic states remain poorly understood. Here, we investigated the role of ubiquitination, as this PTM is implicated in the regulation of numerous cell surface–expressed ion channels by triggering endocytosis (4). Using comprehensive unbiased MS profiling, we identified multiple monoubiquitinated lysine residues that cluster specifically in the IDRs of the intracellular N-terminal and C-terminal domains of the TRPV4 protein. We found that disrupting or enhancing ubiquitination of the N-terminal IDR modulates TRPV4-mediated calcium influx in opposing directions without altering TRPV4 cell surface abundance, revealing a novel mechanism for Ub-mediated tuning of plasmalemmal ion channel activity.

Although ubiquitination has not previously been shown to modulate ion channel activity in the absence of altered trafficking, it has been suspected that this could occur (6, 78). Importantly, the Ub-like PTM SUMO has been reported to modulate activity of plasmalemmal potassium channels without altering surface localization (79, 80, 81), and there is at least one example of a membrane protein, Gap1, that remains monoubiquitinated at the cell surface and is not internalized unless it becomes polyubiquitinated (21, 82). Here, we employed several experimental methods including immunocytochemistry (ICC), cell surface biotinylation, and TIRF microscopy to demonstrate that little alteration of TRPV4 surface abundance was provoked by changes of TRPV4 N-terminal IDR ubiquitination.

Ion channel IDRs are important allosteric modulators of channel activity (83, 84, 85) as well as sites for scaffolding protein interactions (86, 87, 88). Furthermore, protein IDRs are often hot spots for PTMs (89), which can affect the target protein’s binding partners and structural conformation (90). Ubiquitination of protein IDRs is well studied, and evolutionarily conservation of Ub sites within IDRs is favored over ordered regions (91), although the effects of ion channel IDR ubiquitination on channel function are not well explored (33, 87). However, there are numerous examples of monoubiquitination regulating protein function through alteration of protein–protein and protein–lipid interactions in other contexts (18, 19, 20, 92, 93), suggesting that monoubiquitination of the TRPV4 N-terminal IDR may similarly influence channel activity through effects on protein or membrane lipid interactions without affecting surface localization.

The TRPV4 N-terminal IDR is an extended 149 amino acid peptide region that, like other IDRs, is flexible, can undergo conformational changes, and is highly accessible for protein–protein and protein–lipid interactions as well as PTMs (33). Previous studies have demonstrated multiple mechanisms by which TRPV4 IDRs can modulate channel activity: alteration of TRPV4 protein–protein interactions (37, 94), disruption of PIP_2_ interaction (31, 36, 66), and through phosphorylation of specific residues (95, 96, 97). We did not identify specific disruption of protein–protein interactions that accounted for the increased ion channel activity observed with disruption of N-terminal IDR ubiquitination. While changes in PACSIN1 interaction were observed with the 5K-IDR mutant that displays increased channel function, similar disruption of binding was seen with the more limited K130, 136R mutant which did not have increased channel function. These data indicate that the regulatory interplay between TRPV4 ubiquitination and channel activity is more complex than simply abrogating interactions with desensitizing binding partners. On the other hand, mutation of the PIP_2_-binding domain in the 5K-IDR mutant rescued the gain-of-ion channel function observed in the antagonist withdrawal paradigm, suggesting that ubiquitination of the N-terminal IDR may regulate channel activity at least in part through alterations in PIP_2_ interactions. Our data argue that multiubiquitination of the TRPV4 N-terminal IDR modulates channel activity in the absence of altering cell surface abundance, thereby allowing TRPV4 to preserve its membrane scaffolding functions and ability to recruit binding partners such as RhoA, among others (32, 35, 40, 59, 65, 98, 99). Together, these results add to the emerging evidence of important modulatory roles of IDRs and provide a novel link between ubiquitination and IDR function in the regulation of ion channel activity.

Our finding of decreased ubiquitination of TRPV4 neuropathy mutants provides further evidence of the importance of Ub in the regulation of ion channel activity. We and others have shown that neuropathogenic mutations cause a gain-of-ion channel function, both *in vitro* and *in vivo* (30, 32, 67). Here, we demonstrate that reduced ubiquitination of neuropathy-causing TRPV4 mutants is not a result of an intrinsic defect in their ability to be ubiquitinated, but is rather a consequence of the gain-of-ion channel function and resultant increased intracellular calcium levels. Restoring ubiquitination of neuropathy mutant TRPV4 through expression of NEDD4 was able to ameliorate increased channel activity. Given that the ubiquitination status of a protein is regulated by the balance of activity of both E3 ligases and deubiquitinating enzymes (DUBs) (100), one possibility is that excessive calcium influx through the mutant channels activates a DUB that deubiquitinates the mutant protein, further contributing to the pathological gain-of-channel function. Future studies will be needed to further define the ubiquitinating enzymes and DUBs that regulate TRPV4 in different cell types and the circumstances in which they are activated. This has particular relevance for emerging therapeutic strategies that aim to specifically modulate ubiquitination, such as designer DUBs and small-molecule inhibitors (101, 102).

Increasingly, protein ubiquitination is appreciated as a complex code with a multiplicity of functional effects. While our studies focus on the functional consequences of monoubiquitination of specific lysines in the TRPV4 N-terminal IDR, there are likely multiple different types of ubiquitination events involving TRPV4 that contribute to its regulation. Specific ubiquitinated lysine residues were also very consistently observed in the C-terminal IDR of TRPV4, although their functional relevance is currently unclear. With the broad physiologic role of TRPV4 in different tissues and pathological states, and an increasing appreciation of the diverse functions of IDRs in regulating channel activity, understanding how TRPV4 function is modulated by its IDRs could serve as a gateway to studying the role of IDR regulation of other ion channels. Future studies dissecting what may be a complex Ub code targeting ion channel IDRs under different conditions and in different cell types will have important implications for understanding ion channel function in health and disease.

## Experimental procedures

### Mice

All animal procedures followed the National Institutes of Health guidelines and were approved by the Johns Hopkins University Animal Care and Use Committee. Conditions in the mouse facility were maintained at 42% humidity, 22.2 °C, and a 14:10 h light–dark cycle. TRPV4-null mice (Fig. S1*A*) (41) were kindly provided by Michael Caterina, and HA-Ub mice (48) were a generous gift from Hidde Ploegh. A second TRPV4 KO mouse line (Fig. S1*B*) was generated by the Jackson Laboratory and previously characterized by our laboratory (42).

### Drosophila

Flies were raised on standard cornmeal-molasses food. Experiments were performed at 25 °C with a 12/12 h day/night cycle. Transgenic UAS-TRPV4 R269C, UAS-TRPV4 WT, and C155-GAL4 pan-neuronal driver flies were previously described (67).

### Mammalian expression plasmids

TRPV4-FLAG in pcDNA3.1 was previously described (103). TRPV4-V5-FLAG was generated using Gibson Assembly Master Mix (New England Biolabs), by inserting the V5 tag sequence into the middle position of the first TRPV4 extracellular loop. TRPV4-V5-EGFP was generated by replacing the FLAG tag of the TRPV4-V5-FLAG construct with EGFP cloned from pEGFP-C1 (Clontech), as previously described (32). TRPV4 K>R mutants (All-K-IDR; 5K-IDR; 5K-CTD; K130, 136R), N-terminally Myc-tagged ITCH (NP_001311127.1), and ITCH C830A (ITCH-CI) were synthesized by GenScript in the pCI-Neo vector. Myc-NEDD4 and Myc-NEDD4-CI were generated by replacing the N-terminal HA tag in pCI-HA-NEDD4-CI (Addgene; plasmid nos.: 27002 and 26999) with a Myc tag using Gibson Assembly Master Mix (New England Biolabs). The expression plasmids for PACSIN1-Myc, PACSIN2-Myc, and PACSIN3-Myc were generous gifts from Dr Markus Plomann (University of Cologne).

The following plasmids were obtained from Addgene: pRK5-HA-Ub-K0 (17603), pRK5-HA-Ubiquitin-WT (catalog no.: 17608), pLenti-puro-HA-Ubiquitin (catalog no.: 74218-LV), pCI-HA-NEDD4 (catalog no.: 27002), pCI-HA-NEDD4-DD (catalog no.: 26999), HA-Ubiquitin (catalog no.: 18712), and RhoA-Myc (catalog no.: 12962).

### Transient transfection

HEK293T, MN-1, and MDCK cells were cultured in Dulbecco’s modified Eagle’s medium supplemented with 10% (v/v) fetal bovine serum and penicillin/streptomycin at 37 °C with 5% CO_2_. For Ub assays, calcium imaging, and TIRF microscopy, HEK293T cells were plated at 4.5 × 10^5^ cells/ml in 6-well plates 24 h prior to transfection. Cells were transfected using Lipofectamine LTX (Thermo Fisher Scientific) as per the manufacturer’s instructions with a total of 2.5 μg DNA/well, comprising 0.5 μg of TRPV4 plasmid, 0.7 μg of E3 ligase plasmid (where required), and 1.3 to 2.0 μg of empty pcDNA3.1 vector. For calcium imaging and ICC experiments, cells were trypsinized at 4 h post-transfection and replated at a density of 1.86 × 10^5^ cells/ml on poly-l-ornithine-coated coverglass in 12-well plates. For experiments requiring culturing of transfected cells in TRPV4-specific antagonists, HC067047 (0.5 μM; MilliporeSigma) was applied at 4 h post-transfection.

MDCK cells were transfected with GenJet II transfection reagent (SignaGen) as per the manufacturer’s instructions. Positively transfected cells were selected with 1000 μg/ml G418 sulfate (Thermo Fisher Scientific) 6 h after transfection and allowed to grow to confluency up to 72 h post-transfection.

50B11 cells were cultured in Neurobasal medium supplemented with 5% fetal bovine serum, 2% B-27 supplement, 1% GlutaMAX, and 0.2% glucose at 37 °C with 5% CO_2_. For calcium imaging experiments, 50B11 cells were transfected with Fugene 6 (Promega) as per the manufacturer’s instructions. Cells were plated at a density of 2 × 10^5^ cells/ml in 6-well plates and transfected at 24 h postplating with a total of 2 μg DNA/well, comprising 0.7 μg of GFP plasmid (to identify transfected cells), 0.7 μg of E3 ligase plasmid, and 0.6 μg of empty pcDNA3.1 vector. All cells were assayed 24 h after transfection. For cell-based Ub assays, pLenti Puro HA-Ubiquitin lentivirus was added to 50B11 cells at 0.5 multiplicity of infection for 48 h. Transduced cells were selected with 2 μg/ml puromycin in media changed every 2 days until cells reached 80% confluency.

### IP

At 24 h post-transfection, HEK293T, 50B11, and MDCK cells were washed three times in PBS containing 0.9 mM CaCl_2_ and 0.5 mM MgCl_2_ (PBS++) and then lysed in radioimmunoprecipitation assay (RIPA) buffer (MilliporeSigma) supplemented with Halt Protease and Phosphatase Inhibitor Cocktail (Thermo Fisher Scientific) and the DUB inhibitor PR-619 (50 μM; MilliporeSigma). Lysates were centrifuged at 17,000*g* for 15 min, and supernatants were then incubated for 1 h at 4 °C with rotation in 25 μl of Dynabeads Protein G (Thermo Fisher Scientific) preconjugated with 2 μg of anti-FLAG M2 antibody. Beads were then washed three times with 0.2% Tween-20 in PBS and transferred to new tubes. Immunoprecipitated protein was eluted from the beads in sample buffer (RIPA buffer containing 1× Laemmli sample buffer and 10% 2-mercaptoethanol) at 70 °C for 10 min. For IP of TRPV4 from mouse choroid plexus, mice were deeply anesthetized with isoflurane and euthanized by cervical dislocation. Choroid plexus tissues were dissected from the lateral and fourth ventricles, lysed in 50 μl of ice-cold RIPA buffer supplemented with protease inhibitor cocktail, and homogenized with a polypropylene pestle (MilliporeSigma). Samples were then processed as described previously, with incubation in Dynabeads Protein G preconjugated with anti-TRPV4 antibody (Abcam; catalog no.: ab39260). For *Drosophila*, 30 heads were collected from 3-day-old adult flies expressing TRPV4 variants selectively in neurons (C155-GAL4 pan-neuronal driver). Lysis and IP protocols were identical to those described previously for mouse choroid plexus tissue.

### WB

Whole cell lysates (inputs) were reduced in Laemmli sample buffer with 10% 2-mercaptoethanol for 10 min at 70 °C. Lysates and eluates were resolved on 4 to 15% or 7.5% TGX gels (Bio-Rad) and then transferred to polyvinylidene difluoride membranes (Thermo Fisher Scientific). Membranes were blocked with 5% bovine serum albumin (BSA) in Tris-buffered saline containing 0.1% Tween-20 (TBST) for 1 h at room temperature with rocking. For experiments requiring the Ub-horseradish peroxidase (HRP) primary antibody, blocking was performed in 1% BSA in TBST. Primary antibodies were applied overnight at 4 °C in TBST containing 2.5% BSA, and secondary antibodies were applied for 1 h at room temperature in TBST. Membranes were developed with SuperSignal West Pico Substrate (Thermo Fisher Scientific) and imaged using an ImageQuant LAS 4000 system (GE Healthcare). Densitometric analyses were performed using open source FIJI ImageJ software.

### ICC

At 24 h post-transfection, cells were washed briefly with PBS++ on ice and then blocked in PBS++ containing 0.1% BSA for 30 min at 4 °C. Cells were then incubated in blocking solution containing mouse anti-V5 antibody (1:5000 dilution) for 1 h at 4 °C, washed three times for 5 min in PBS++, and fixed in 2% paraformaldehyde for 30 min at room temperature. For experiments requiring immunolabeling of intracellular proteins (as in the experiments with NEDD4 coexpression, Fig. 6), cells were permeabilized with 0.3% Triton X-100 for 15 min, blocked in TBS containing 1% BSA and 0.2% milk for 1 h at room temperature, and then incubated in TBS blocking solution containing the required primary antibody for 1 h at room temperature. All cells were then washed and stained with secondary antibodies for 1 h at room temperature in TBS blocking solution. Coverslips were mounted on slides with Prolong Diamond Antifade Medium with 4′,6-diamidino-2-phenylindole (Thermo Fisher Scientific) and imaged using a Zeiss LSM 800 Airyscan confocal microscope. Images were quantified in FIJI by selecting individual cells and measuring the mean gray areas for both the surface stain (V5) and total TRPV4 (EGFP). Surface/total ratio was then calculated and reported.

### Flow cytometry

Transfected MN-1 cells were gently harvested on ice using a cell scraper, pelleted, and washed three times with ice-cold PBS++. Immunocytochemical staining of cell surface TRPV4 was performed as described previously using a mouse DyLight 488-conjugated mouse anti-V5 antibody, with washing steps performed by pelleting cells at 156*g* for 5 min at 4 °C. Immunolabeled cells were then analyzed using a FACS Lyric flow cytometer (BD Biosciences).

### Cell surface biotinylation

HEK293T cells were cultured in 10 cm dishes and transfected at 80% confluency using polyethylenimine (Polysciences, Inc). Cell surface biotinylation was performed at 24 h post-transfection with the Pierce Cell Surface Protein Isolation Kit (Thermo Fisher Scientific). Briefly, cells were rinsed twice with PBS++ on ice and then incubated with 0.25 mg/ml EZ-Link Sulfo-NHS-SS-Biotin with gentle rotation (30 rpm) for 30 min at 4 °C. Excess biotin was quenched with three successive washes of 50 mM glycine in PBS. Cells were subsequently lysed in RIPA buffer supplemented with protease inhibitor cocktail (MilliporeSigma) for 30 min on ice. Lysates were transferred to microcentrifuge tubes, sonicated at low power, and cleared by centrifugation at 17,000*g* in a refrigerated bench-top microcentrifuge. About 10% of the lysate volume was reserved as input, whereas the remainder was incubated with NeutrAvidin agarose slurry with end-over-end rotation for 2 h at 4 °C. The slurry was then washed three times each by centrifugation with wash buffer and high-salt (0.5 M NaCl) wash buffer, respectively. Biotinylated proteins were eluted in Laemmli sample buffer supplemented with 50 mM DTT. Lysates were heated at 70 °C for 10 min prior to WB analysis, as described previously. Surface/total TRPV4 ratios were quantified by densitometry using FIJI software. Normalized values for surface and total TRPV4 were calculated by dividing TRPV4 band intensity by the corresponding transferrin receptor band intensity. The ratio of surface to total TRPV4 was then calculated by dividing the normalized TRPV4 surface value by the normalized total value (as reported in Fig. S5, *B* and *C*).

### Calcium imaging

Calcium imaging was performed using a Zeiss Axio Observer.Z1 inverted microscope equipped with a Lambda DG-4 wavelength switcher (Sutter Instrument Company). Cells were loaded with Fura-2 AM (8 μM; Thermo Fisher Scientific) for 60 min at 37 °C in culture medium. For antagonist withdrawal assays, 0.5 μM HC067 was also included during Fura-2 loading. Coverslips were then transferred to the microscope and placed into calcium imaging buffer (150 mM NaCl, 5 mM KCl, 1 mM MgCl_2_, 2 mM CaCl_2_, 10 mM glucose, 10 mM Hepes, and pH 7.4). For antagonist withdrawal experiments, a gravity flow system was used to wash out HC067-containing calcium imaging buffer from the coverslip chamber continuously over the course of an hour. Excess buffer was pumped out with a peristaltic pump (New Era Pump Systems). For calcium-free experiments, CaCl_2_ was omitted from the buffer. Cells were imaged every 10 s for the first 6 min, with 1 min of baseline and 5 min following washout of antagonist. The imaging rate was then slowed to once per minute for the following 25 min and finally once every 5 min for the final 30 min of imaging. Calcium levels at each time point were computed by determining the ratio of Fura-2 AM emission at 340 nM divided by the emission at 380 nM. Data were expressed as either raw Fura ratio or change in final Fur-2 ratio from initial Fura-2 ratio. Images were processed using AxioVision 4 (Zeiss).

For HTS treatment, three volumes of NaCl-free calcium imaging buffer were added to one volume of standard calcium imaging buffer for a final NaCl concentration of 37.5 mM. For GSK101 treatment, GSK101-containing buffer (MilliporeSigma) was added 1:1 (v:v) to the imaging chamber to achieve the appropriate final concentration. Cells were imaged every 10 s, first taking baseline measurements for 30 s, then imaging for 5 min after treatment.

### Antibodies and reagents

Primary antibodies utilized include polyclonal rabbit anti-FLAG (WB: 1:1000 dilution; Cell Signaling Technology; catalog no.: 2368), monoclonal mouse anti-FLAG (ICC: 1:1500 dilution; Cell Signaling Technology; catalog no.: 8146), monoclonal mouse anti-Myc (WB: 1:1000 dilution; Cell Signaling Technology; catalog no.: 2276), polyclonal rabbit anti-Myc (ICC: 1:1000 dilution; Cell Signaling Technology; catalog no.: 2272), monoclonal rat anti-HA (WB: 1:1000 dilution; IP 5 μg/ml; Roche; catalog no.: 11867423001), monoclonal mouse anti-V5 (WB and ICC: 1:5000; Thermo Fisher Scientific; catalog no.: R960-25), monoclonal DyLight 488-conjugated anti-V5 (flow cytometry; 1:100 dilution; Thermo Fisher Scientific; catalog no.: MA5-15253-D488), polyclonal rabbit anti-β-actin (WB: 1:1000 dilution; Cell Signaling Technology; catalog no.: 4967), monoclonal rabbit antitubulin (WB: 1:1000 dilution; Cell Signaling Technology; catalog no.: 2144S), monoclonal mouse anti-FLAG M2 (IP: 5 μg/ml; MilliporeSigma; catalog no.: F1804), polyclonal rabbit anti-TRPV4 (WB: 1:1000 dilution, IP: 5 μg/ml; Abcam; catalog no.: ab39260), monoclonal mouse anti-Ub (P4D1)-HRP conjugate (WB: 1:1000; Enzo Life Sciences; catalog no.: BML-PW0935-0025). Secondary antibodies used were HRP-conjugated monoclonal mouse anti-rabbit immunoglobulin G, light-chain specific (WB: 1:75,000 dilution; Jackson ImmunoResearch; catalog no.: 211-032-171), HRP-conjugated goat antimouse immunoglobulin G, light-chain specific (WB: 1:75,000 dilution; Jackson ImmunoResearch; catalog no.: 211-032-174), Alexa Fluor 568 goat anti-rabbit (ICC: 1:1000 dilution; Thermo Fisher Scientific; catalog no.: A-11011), and Alexa Fluor 647 donkey antimouse (ICC: 1:1000 dilution; Thermo Fisher Scientific; catalog no.: A31571). TRPV4-specific reagents include HC067047 (MilliporeSigma; catalog no.: SML0143) and GSK1016790A (MilliporeSigma; catalog no.: G0798).

### TIRF imaging

Cells were plated on 35 mm glass bottom dishes (#1.5 coverglass; MatTek; catalog no.: P35G-1.5-14-C) and transfected as described previously. One hour before imaging, cells were loaded with Cal590 AM (1 μg/μl; AAT Bioquest) and subsequently imaged in calcium imaging buffer 24 h after transfection. The evanescent wave for TIRF was established using micromirrors positioned below a high numerical aperture apochromat objective (100×, 1.5 numerical aperture; Olympus) mounted on a Nikon Eclipse microscope running NIS-Elements software (Nikon). TRPV4-V5-EGFP was excited by a 488 nm Argon laser line, and Cal590 was excited by a 561 nm 0.5 W fiber laser. Emissions were collected through a GFP/red fluorescent protein/GFP–red fluorescent protein FRET filter set using an Evolve 512 EMCCD camera (Photometrics). Images were captured with a 50 ms exposure every 5 s. The surface expression level of TRPV4 was determined for one to three cells from a random field of view per dish. Mean fluorescence intensity was analyzed using FIJI. GSK101 treatment was performed as in the calcium imaging experiments described previously.

### Cloning, expression, and purification of recombinant proteins

#### Full TRPV4 N terminus and TRPV4 ARD

Recombinant full TRPV4 N terminus (residues 2–397) and ARD (residues 148–397) proteins were prepared as previously described (32). Briefly, constructs were expressed in *Escherichia coli* BL21-Gold(DE3) in terrific broth medium supplemented with 0.04% glucose and 100 μg/ml ampicillin. Cells were grown at 37 °C to an absorbance of 0.4 at 600 nm, moved to 20 °C, and grown to an absorbance of 0.8 at 600 nm at which point they were induced with 75 μM IPTG. Growth continued at 20 °C for 16 h. For purification, cells were suspended in lysis buffer (20 mM Tris [pH 8], 20 mM imidazole, 300 mM NaCl, 0.1% [v/v] Triton X-100, 1 mM DTT, 1 mM benzamidine, 1 mM PMSF, DNase, RNase, and SIGMAFast protease inhibitor cocktail) and sonicated on ice. The cell lysate was loaded onto a nickel–nitrilotriacetic acid gravity flow column (Qiagen), and protein was ultimately eluted with 500 mM imidazole and dialyzed (20 mM Tris [pH 8], 20 mM imidazole, 300 mM NaCl, 1 mM DTT, and 1 mM EDTA) overnight at 4 °C in the presence of Ulp-1 protease (molar ratio, 15:1). The tagHis6-SUMO tag was cleaved from the TRPV4 protein and further purified by size-exclusion chromatography (HiLoad prep grade 16/60 Superdex200; GE Healthcare) in 20 mM Tris (pH 7), 300 mM NaCl, 10% (v/v) glycerol, and 1 mM DTT. Protein purity was confirmed *via* SDS-PAGE and subsequent Coomassie blue staining.

#### ITCH

Full-length ITCH (residues 1–903) was synthesized by GenScript in a pET11a vector with an N-terminal His6-tobacco etch virus (TEV) cleavage site. Briefly, the construct was expressed in *E. coli* BL21-Gold(DE3) in LB medium supplemented with 100 μg/ml ampicillin. Cells were grown at 37 °C to an absorbance of 0.8 at 600 nm, moved to 18 °C, and were induced with 250 μM IPTG. Growth continued at 18 °C for 16 h. For purification, cells were suspended in lysis buffer (50 mM Tris [pH 8], 20 mM imidazole, 200 mM NaCl, 0.1% [v/v] Triton X-100, 5% [v/v] glycerol, 0.1% [v/v] dodecyl maltoside, 1 mM DTT, 1 mM benzamidine, 1 mM PMSF, DNase, RNase, and SIGMAFast protease inhibitor cocktail) and sonicated on ice. The cell lysate was loaded onto a nickel–nitrilotriacetic acid gravity flow column (Qiagen), and protein was ultimately eluted with 500 mM imidazole and dialyzed (50 mM Tris [pH 8], 20 mM imidazole, 200 mM NaCl, 1 mM DTT, and 1 mM EDTA) overnight at 4 °C in the presence of TEV protease (molar ratio of 30:1). The His6-TEV tag was cleaved from the TRPV4 protein and further purified by size-exclusion chromatography (HiLoad prep grade 16/60 Superdex200; GE Healthcare) in 50 mM Tris (pH 8), 200 mM NaCl, 1.5% (v/v) glycerol, and 1 mM DTT. Protein purity was confirmed *via* SDS-PAGE and subsequent Coomassie blue staining.

#### NEDD4 and Ub pathway components

Recombinant NEDD4, UBE1, UbcH5c, and Ub were generous gifts of the Cole Laboratory (Harvard Medical School) and generated as previously described (104).

### Cell-free Ub assays

Recombinant proteins were prepared as described previously and stored at −80 °C. Reactions were performed in microcentrifuge tubes in final volumes of 20 μl at 30 °C. Reactions contained 40 mM Tris–HCl (pH 7.5), 50 mM NaCl, 0.5 mM Tris(2-carboxyethyl)phosphine, 5 mM MgCl_2_, 5 mM ATP, 100 μM Ub, 50 nM E1 protein (UBE1), 1 μM E2 protein (UbcH5c), and 1 μM E3 protein (NEDD4 or ITCH). Reactions were prepared without E1 enzyme, and a time 0 sample was taken (5 μl). E1 enzyme was added to initiate the reaction, and various time points were taken and quenched by adding Laemmli sample buffer (Bio-Rad) with 10% 2-mercaptoethanol to the mixture and boiling for 5 min. Samples were then run on SDS-PAGE gels and stained with Coomassie SimplyBlue SafeStain (Thermo Fisher Scientific).

### MS sample preparation: in-gel digestion

Proteins in gel bands (approximately 1 μg) were destained and reduced in 100 μl of 50 mM DTT in 50% MeOH at 54 °C for 45 min while shaking. To confirm that the alkylating reagents did not affect the identification of modified lysines, proteins within gel bands were alkylated with various alkylating reagents independently, including 200 mM S-methyl thiomethanesulfonate, 200 mM iodoacetamide, and 200 mM chloroacetamide (Table S2) for 15 min in the dark. Gel pieces were processed as previously described (105) and digested with 40 μl trypsin/Lys-C (20 μg in 2 ml 10 mM triethylamine bicarbonate) overnight at 37 °C. Peptides were extracted with three additions of 50 μl of 60% acetonitrile with 0.1% TFA and then dried. Acidified peptides were desalted on a house-made C18 stage tip. Samples were reconstituted in 20 μl of 2% acetonitrile/0.1% formic acid.

Peptides were analyzed by reverse-phase chromatography at 300 nl/min using a 2 to 90% acetonitrile gradient over 90 min on a nano-Orbitrap Fusion Lumos-ETD in FTFT (Thermo Fisher Scientific) interfaced with a nano-LC 1100 system (Thermo Fisher Scientific). Peptides were desalted on a C18 trap (S-10 μM, 120 Å, 75 μm × 2 cm; YMC) and subsequently separated on an in-house packed PicoFrit column (75 μm × 200 mm, 15u, +/−1 μm tip; New Objective) with C18 phase (ReproSil-Pur C18-AQ, 3 μm) 75 μm × 150 mm ProntoSIL-120-5-C18 H column (3 μm, 120 Å; BISCHOFF). Eluting peptides were sprayed into the Lumos-ETD mass spectrometer at 2.7 kV. Survey scans (full MSs) were acquired on Orbitrap within 400 to 1800 Da *m/z* using a data-dependent 3 s cycle time method to monitor the top 15 *m/z* with dynamic exclusion of 10 s. Precursor ions were individually isolated with 0.7 Da (no offset) and fragmented using higher-energy collisional dissociation with an activation collision energy of 30%. Precursor and the fragment ions were analyzed at resolution of 120,000 and 60,000 and an automatic gain control (AGC) of 400 k and 50 k, respectively.

### MS data analysis

Mass spectra were processed by Proteome Discoverer, version 2.4 (Thermo Fisher Scientific) and searched against the RefSeq2021_204_Human protein database containing 71,622 sequences (including the human TRPV4 K>R constructs based on the National Center for Biotechnology Information accession number: NP_067638.3) using Mascot, version 2.7 (Matrix Science). The following were the search criteria: trypsin as enzyme, two missed cleavages, precursor mass tolerance of 5 ppm, and fragment mass of tolerance 0.01 Da. The following were the variable modifications: oxidation on M, Ub (GG) on K, deamidation on N and Q, and static carbamidomethyl on C. Low scoring spectra (less than 1% false discovery rate [FDR]) were filtered and redirected to BYONIC to search a custom database containing all human sequences plus the modified sequences for TRPV4 (*e.g.*, K>R mutants). Peptide identifications were evaluated using Target Decoy Validator based on peptide Q values and with a strict target FDR for peptide-spectral matches and peptides at 0.01. A 1% FDR was calculated using a concatenated decoy database.

### TMT 8-plex analysis: labeling

Peptide digests were reconstituted in 10 μl of 100 mM triethylamine bicarbonate and labeled using TMT 10-plex reagents (Thermo Fisher Scientific; lot no.: TJ68848) following the manufacturer’s instructions. Labeling was quenched with 0.8 μl hydroxylamine, and samples were then combined and dried using a SpeedVac.

### TMT 8-plex analysis: MS method

TMT-labeled peptides (25% of total) were injected and analyzed on a nano-Orbitrap Fusion Lumos-ETD as described previously with the following modifications: 120 min gradient, 360 to 1700 Da *m/z* survey scan, higher-energy collisional dissociation with an activation collision energy of 39%, precursor ions analyzed at resolution of 120,000, 50 ms injection time with an AGC target of 400,000 ions, fragment ions analyzed at 60,000 with a 118 ms injection time. Fragment ion scanning starting at 120 *m/z* with normalized AGC target set to 200% and 100,000 ions.

### TMT 8-plex analysis: MS data analysis

TMT 8-plex mass spectra were processed by Proteome Discoverer and Mascot software as described previously with the following modifications: variable modifications included TMT on K, oxidation on Met, phosphorylation STY, deamidation on N and Q, and GG on K; static modifications included carbamidomethyl on C and TMT on N terminus.

Reporter ions were quantified using S/N values for unique peptides at 5% FDR. Ratios were calculated using an ANOVA based on protein abundance, and either not normalized or normalized on the TRPV4 protein sequence.

### Statistics and reproducibility

Statistical analysis was performed using Prism 9.0.1 (GraphPad Software, Inc). Details of statistical methods and tests are provided in the figure legends. For experiments where no statistical test is provided, the number of times individual experiments were repeated are Figure 1: (*A*) two independent transductions and IPs; (*B*) three independent transfections and IPs; (*C*) choroid plexus lysates from two C57BL/6J and two HA-Ub mice; and (*G*) two independent experiments. Figure 4: (*A*) three independent transfections and IPs. Figure 6, *M* and *N*: data represent six coverslips per condition across two independent transfections; (*O*) and (*P*): data represent more than eight coverslips per condition across eight independent transfections. Figure 7: (*D*) two independent IPs, (*I*) two independent transfections and IPs. Fig. S1, *A* and *B*: choroid plexus lysates from two C57BL/6J and two TRPV4 KO mice. Fig. S2: (*A*) two independent transfections and IPs, (*B*) two independent transfections and IPs. Fig. S3*A*: two independent experiments. (*C*) and (*D*): five independent experiments; (*D*) two independent experiments; (*E*) two independent experiments. Fig. S4: (*D*) one transfection and IP. Fig. S5: (*A*) two independent transfections, staining procedures, and flow cytometry analyses. (*J*) two independent transfections and IPs. Fig. S6: (*A*) two independent transfections, staining procedures, and flow cytometry analyses. Fig. S6: (*B*) Four independent IPs. (*C*) Two independent transfections and IPs.

## Data availability

MS proteomics of ubiquitinated TRPV4 protein can be found in the Figshare database at the following accession number: 10.6084/m9.figshare.16990072.v1 or by following this link https://doi.org/10.6084/m9.figshare.16990072.v1.

## Supporting information

This article contains supporting information.

## Conflict of interest

The authors declare that they have no conflicts of interest with the contents of this article.

## Supporting information

Appendix1_mass spectra and ion tables

Supplemental Table S2

Peptide tables
