# Supplementary material for: Multiubiquitination of TRPV4 reduces channel activity independent of surface localization
Source: J Biol Chem. 2022 Mar 14;298(4):101826. doi: 10.1016/j.jbc.2022.101826 (PMC9010760; doi:10.1016/j.jbc.2022.101826)
Supplement: Appendix1_mass spectra and ion tables [file mmc1.docx]

**Lysine 70**

**
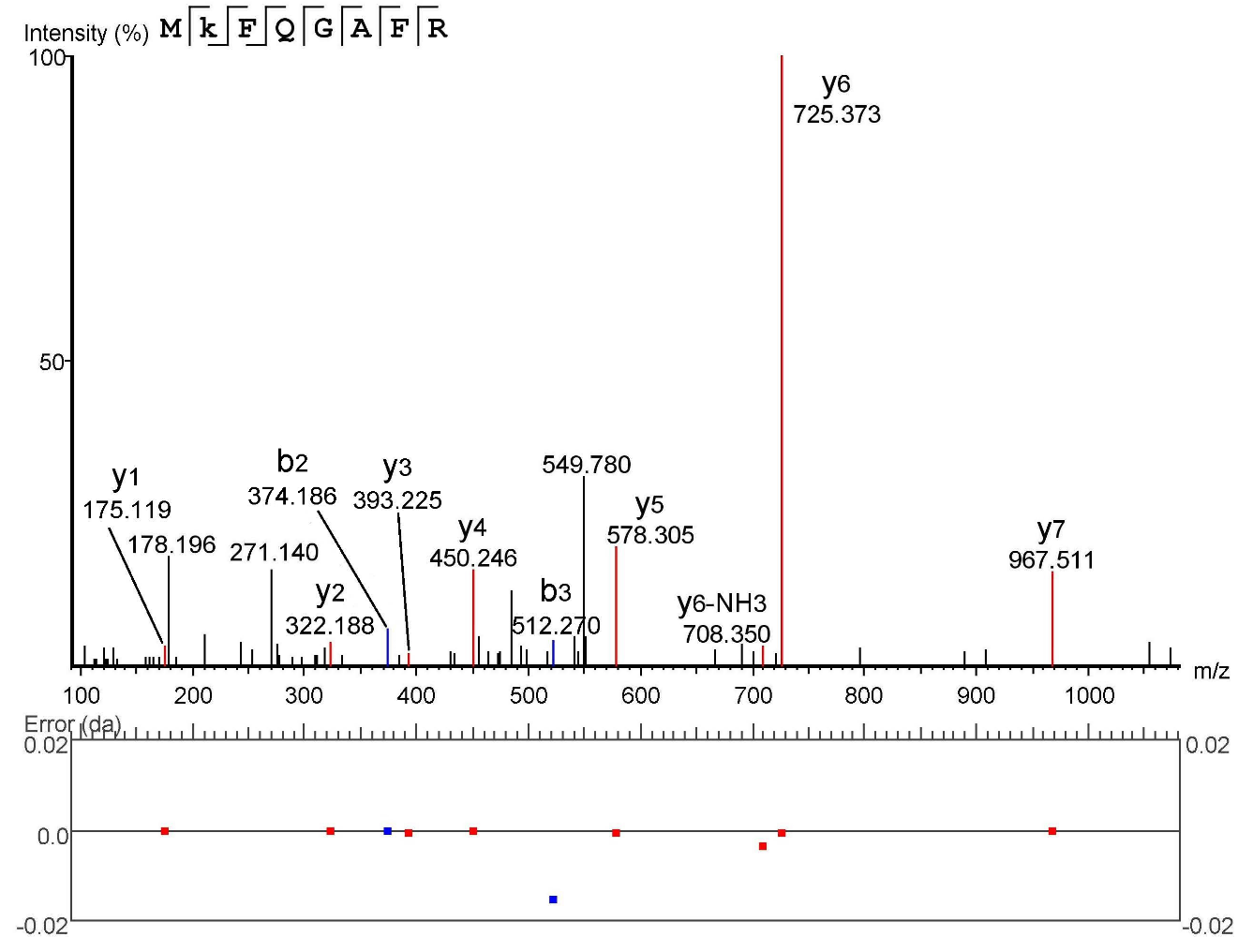
**

**
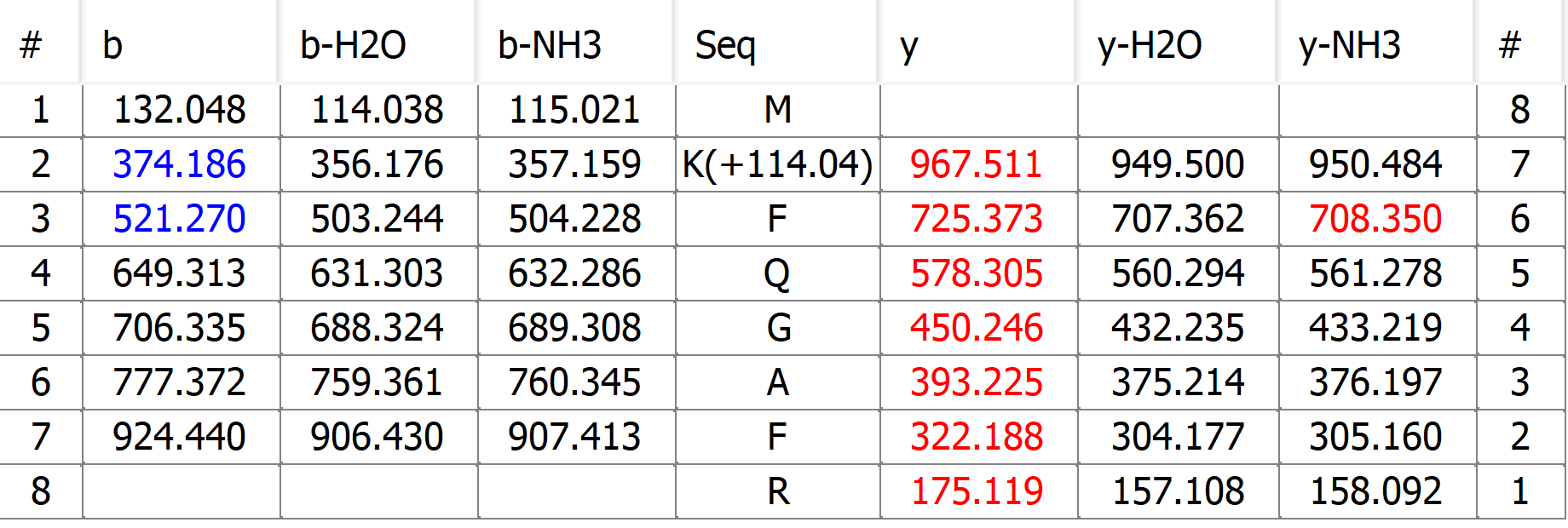
**

**Lysine 77**

**
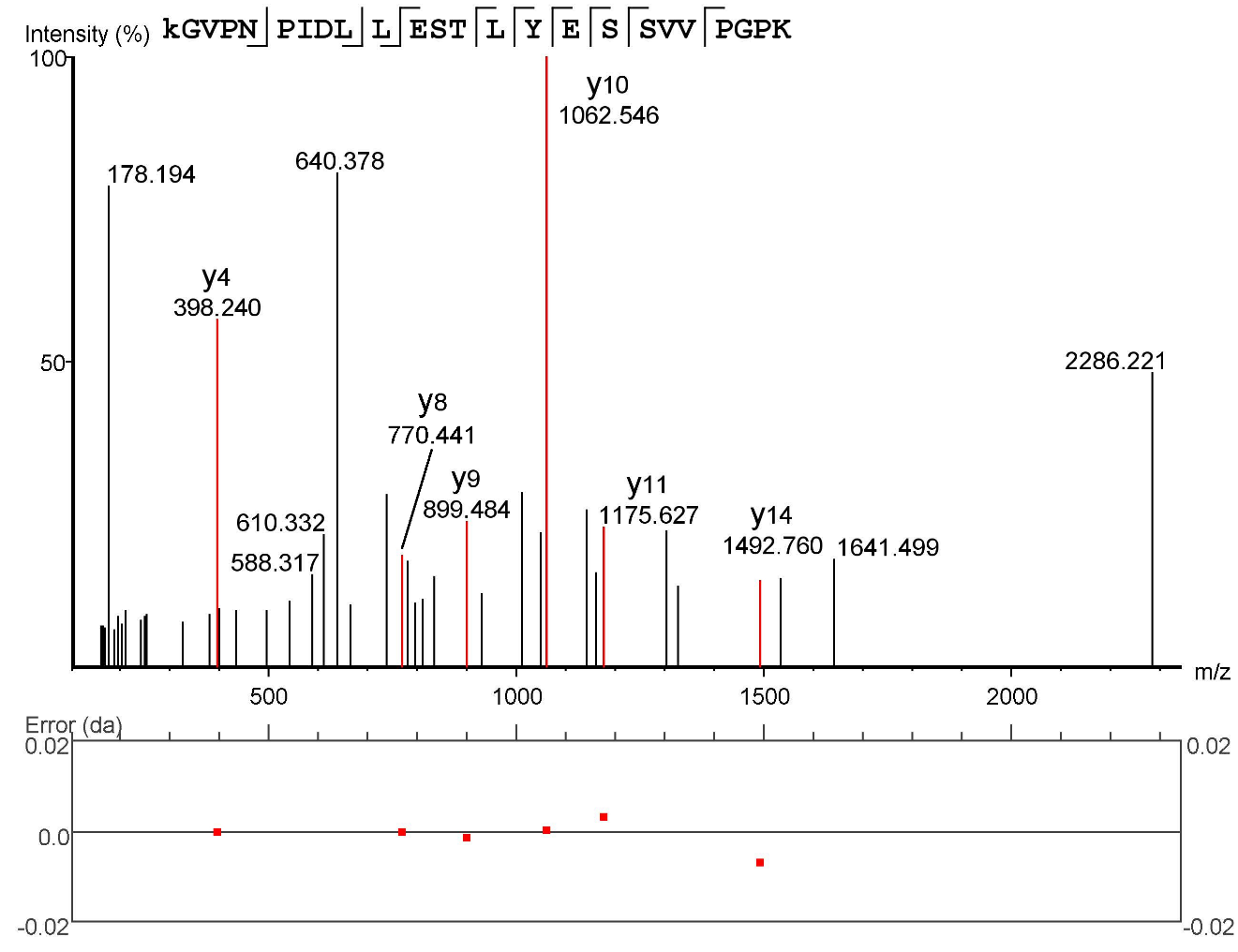
**

**
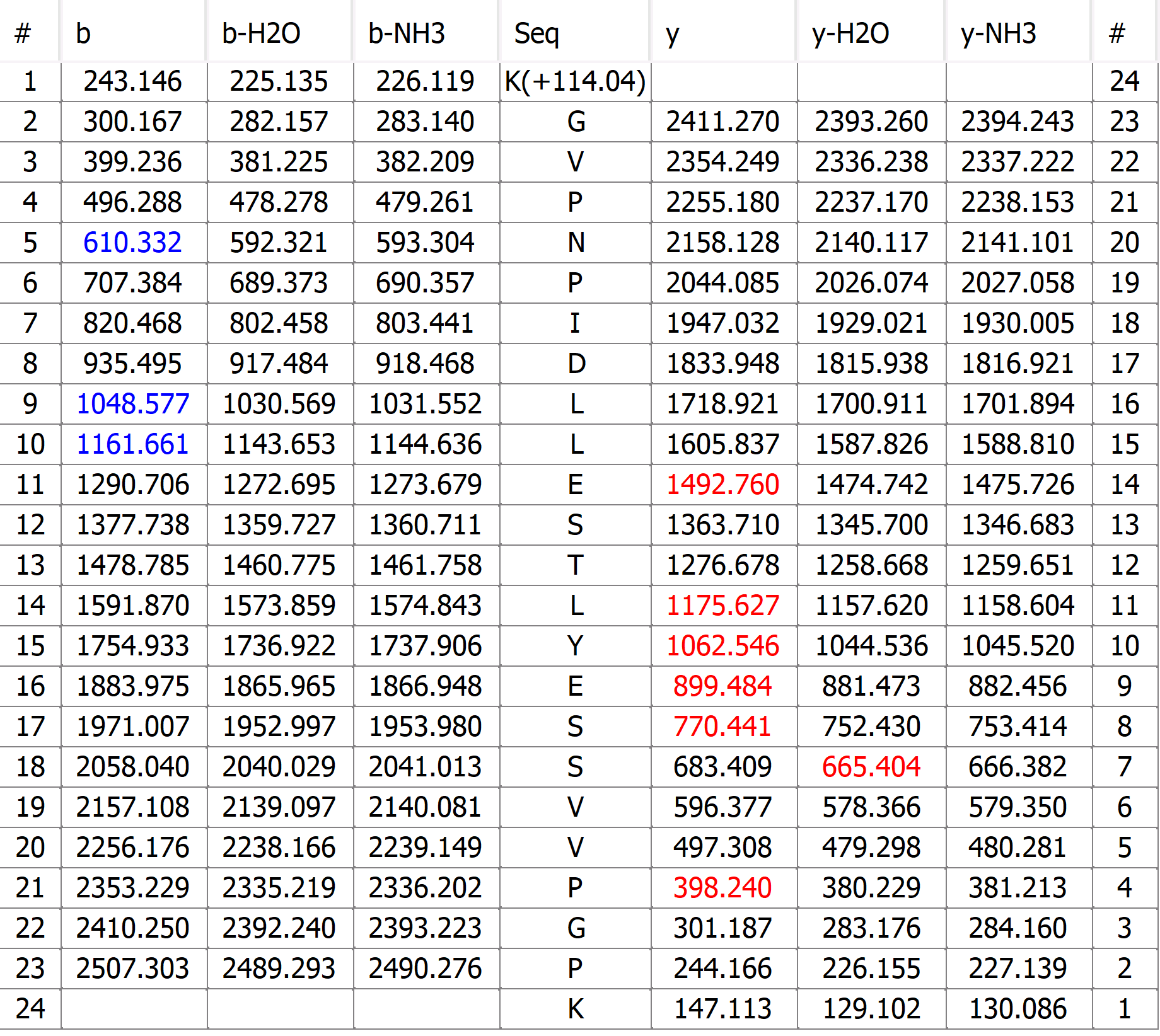
**

**Lysine 100**

**
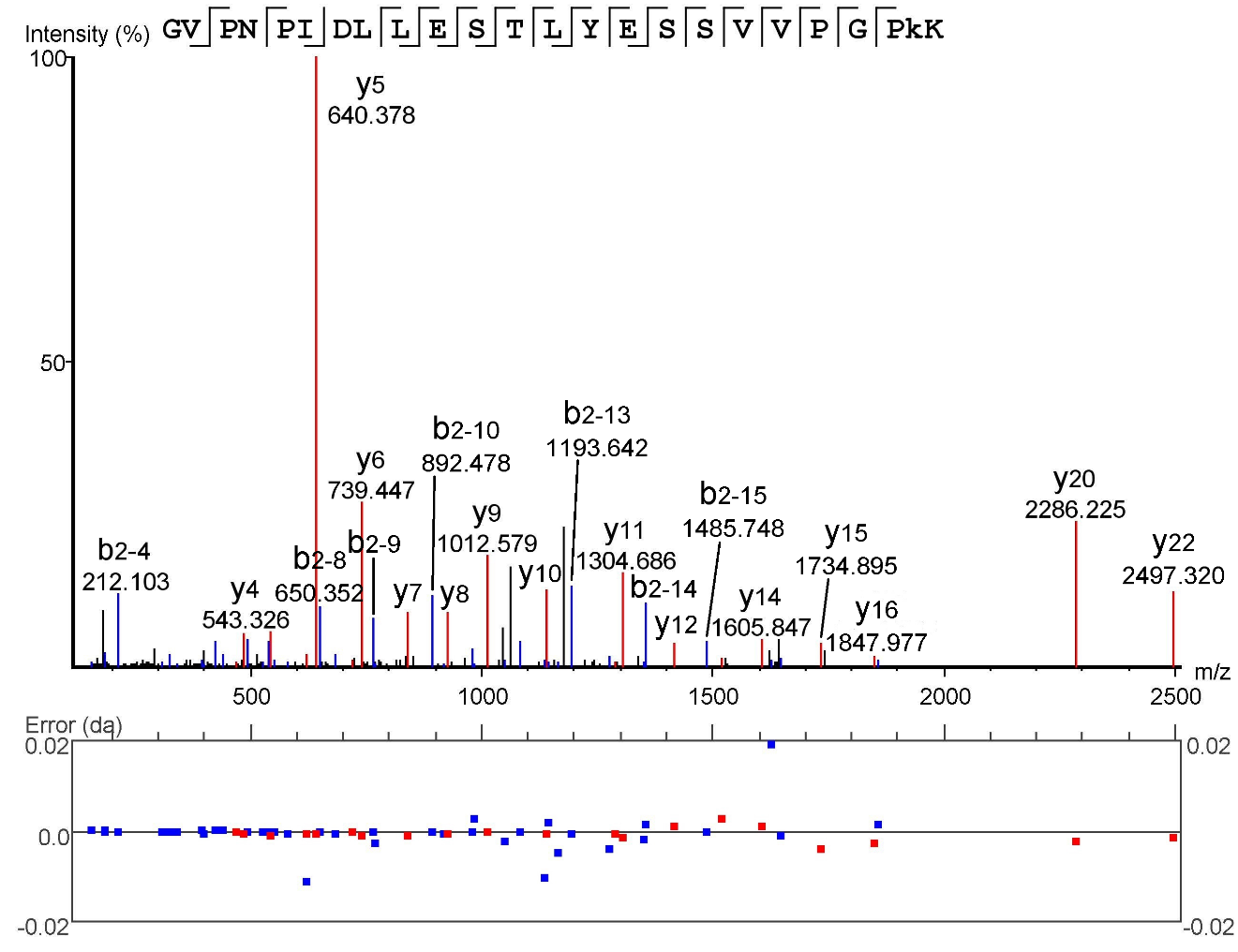
**

**
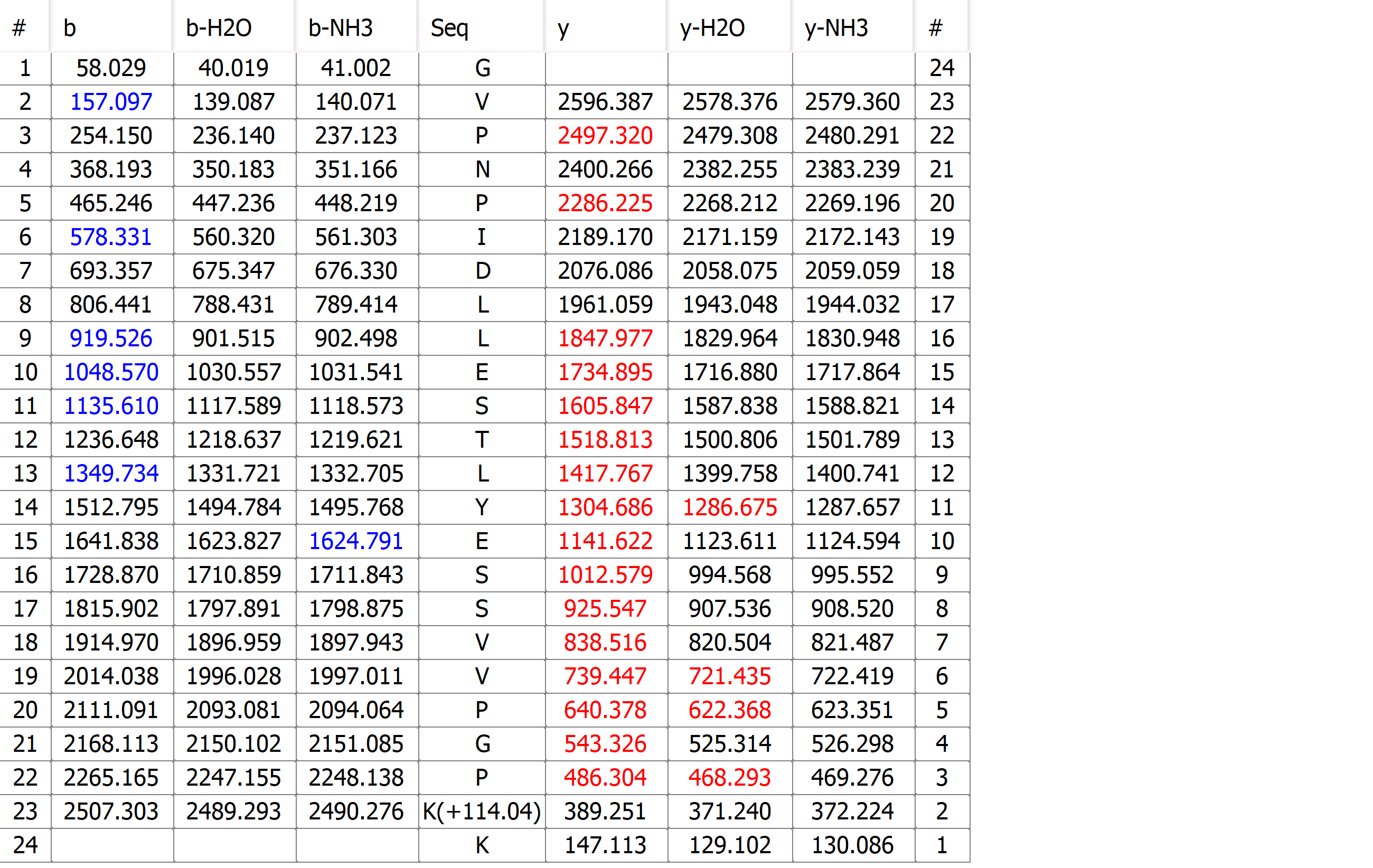
**

**Lysine 101**

**
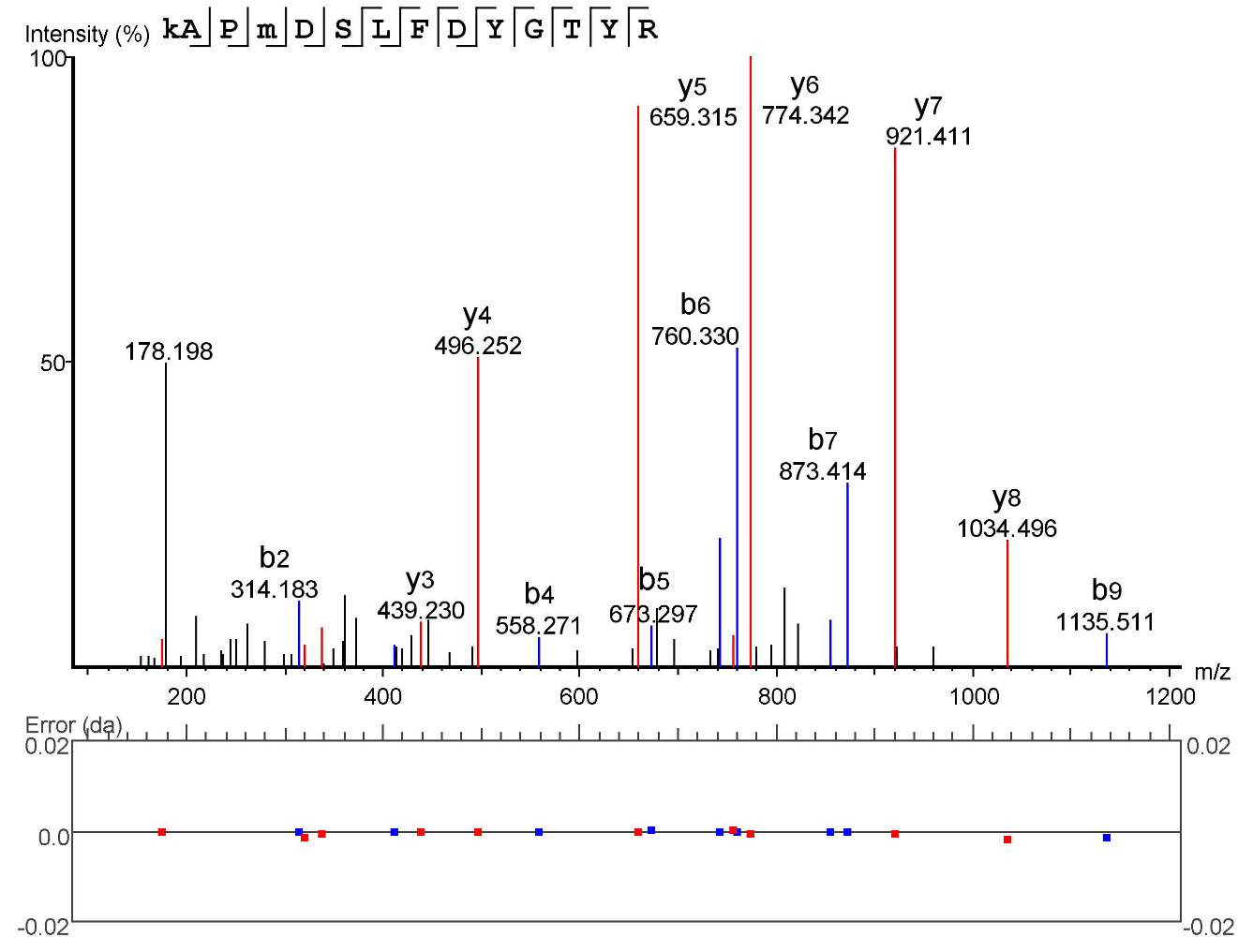
**

**
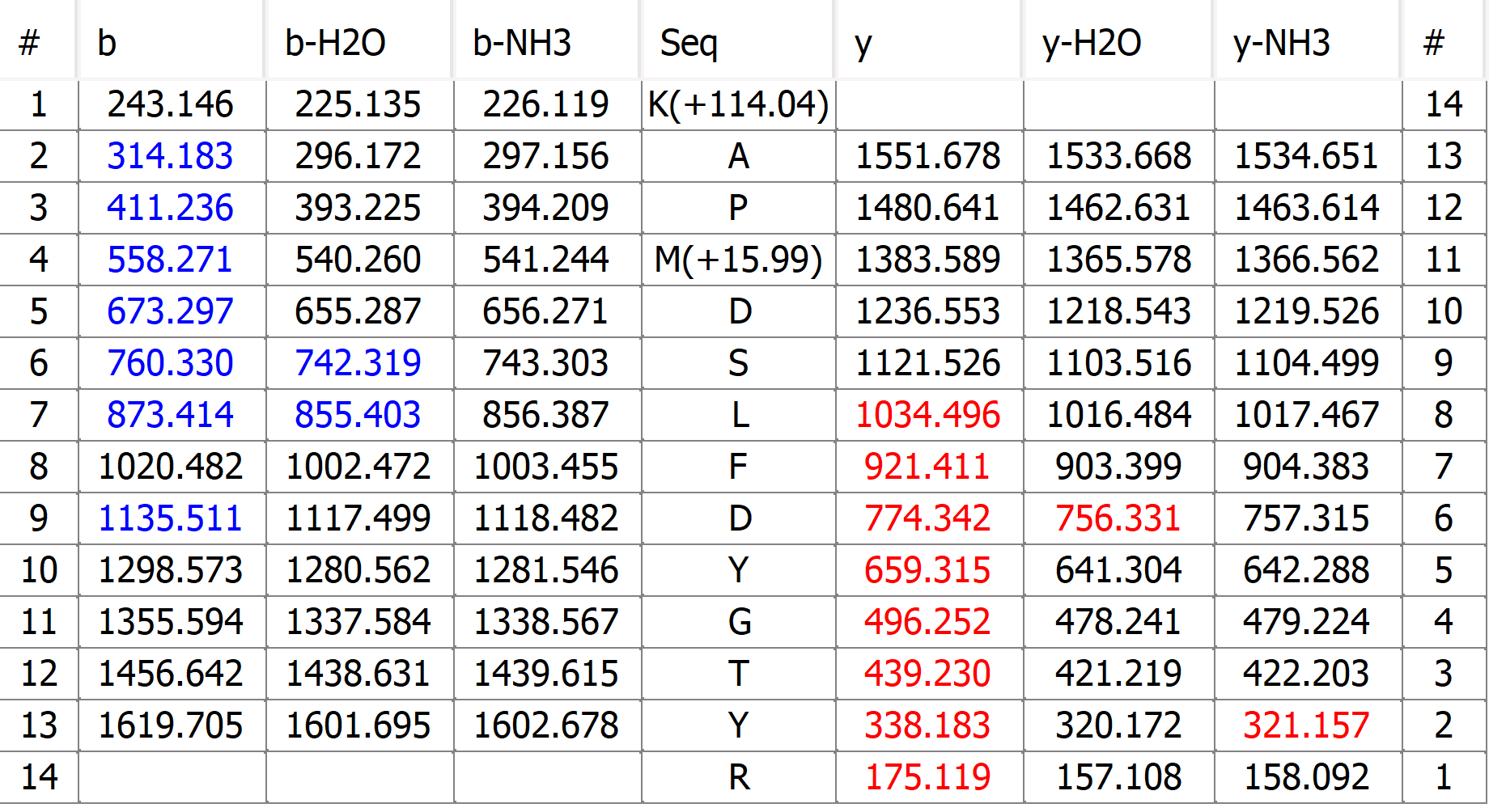
**

**Lysine 130**

**
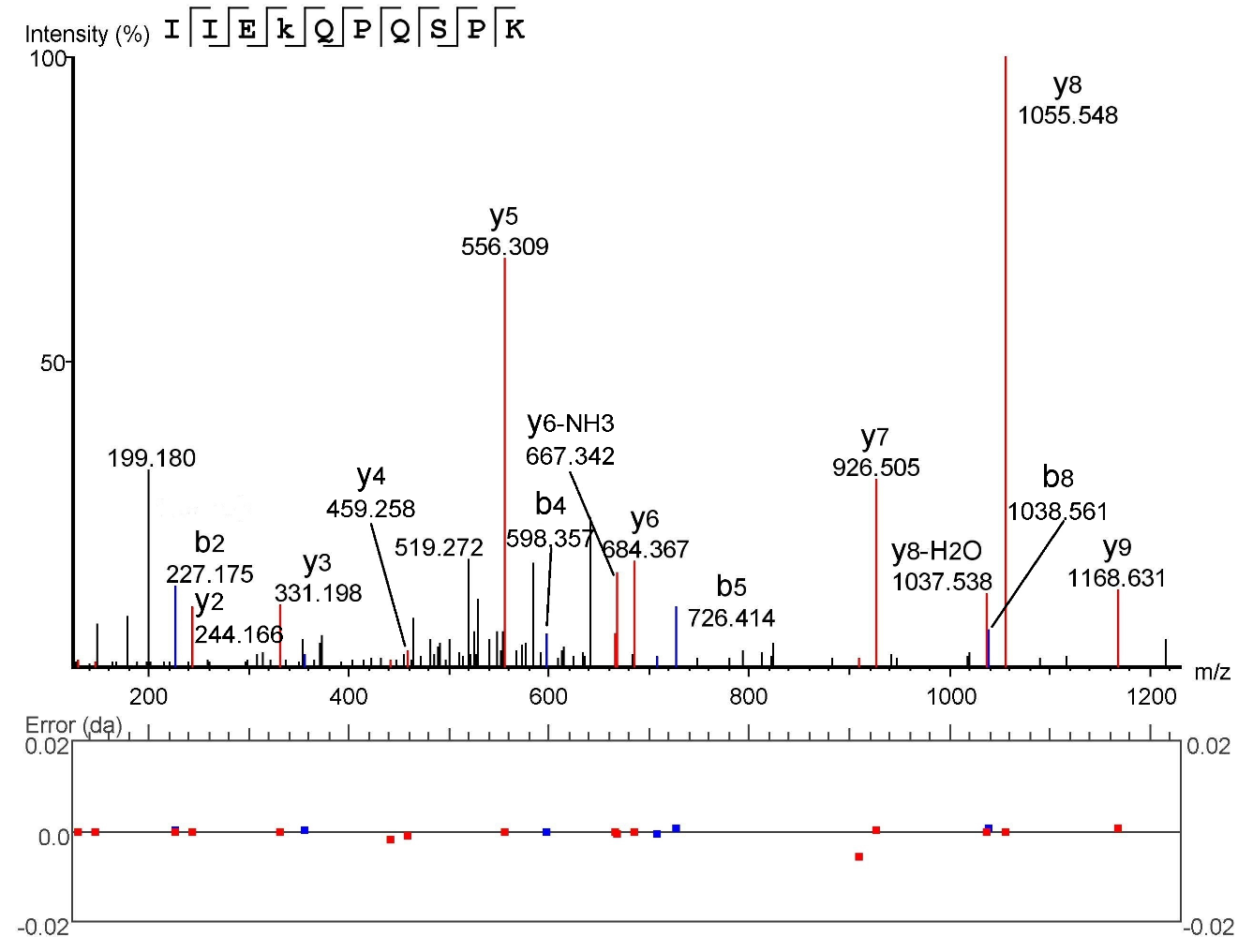
**

**
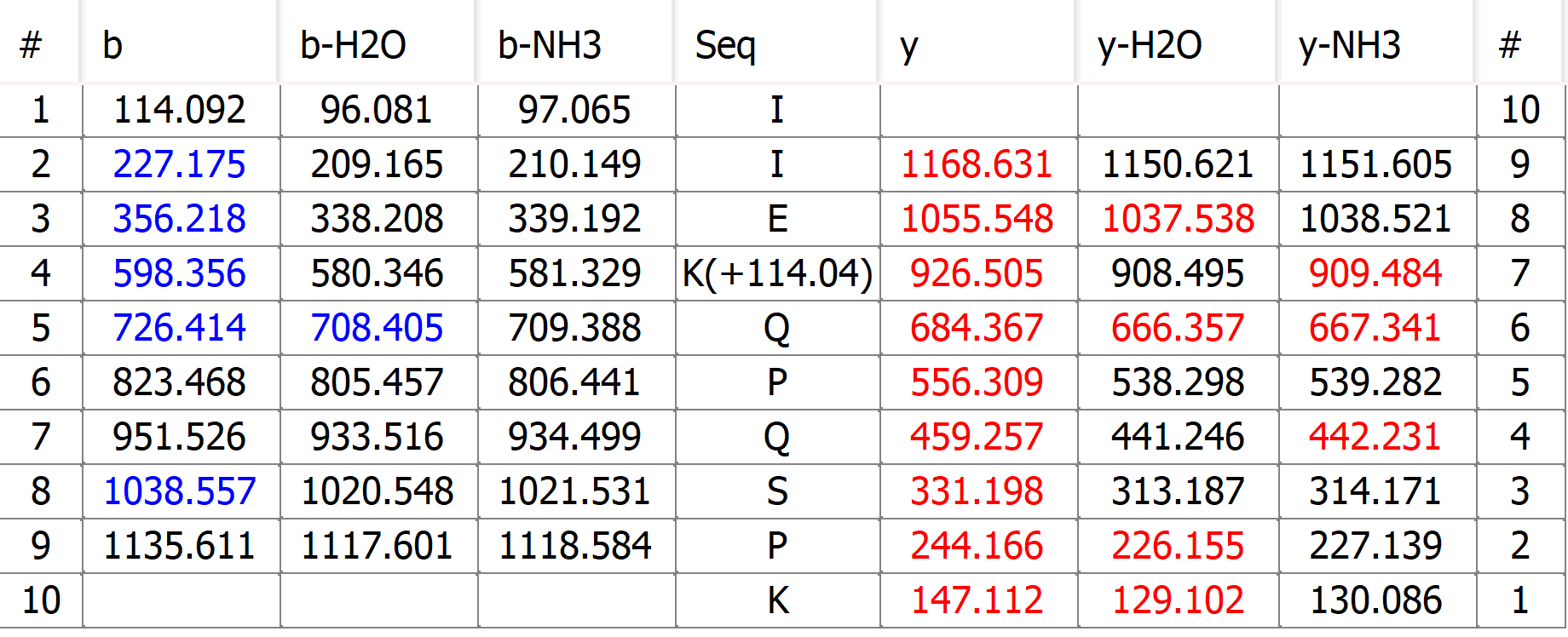
**

**Lysine 136**

**
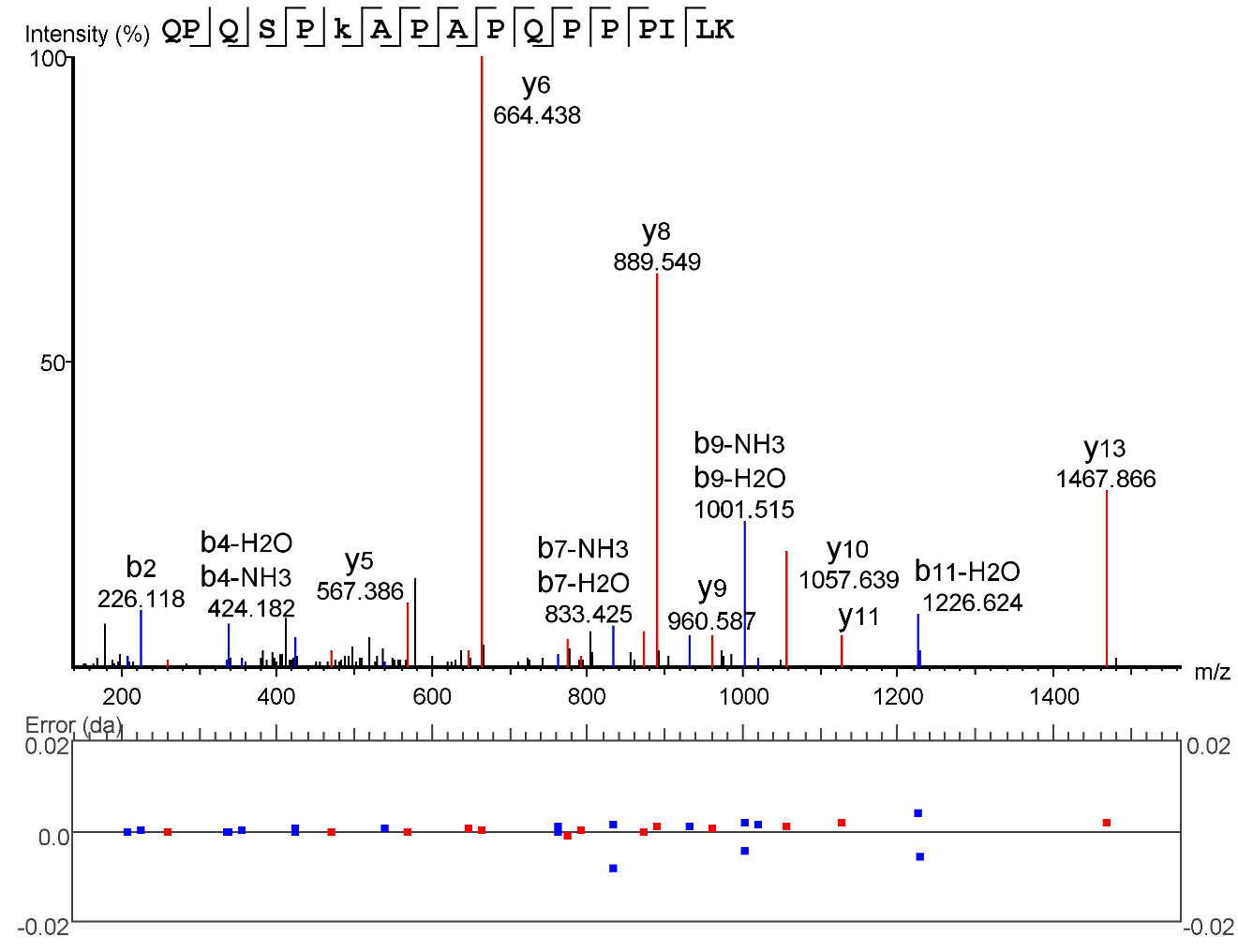
**

**
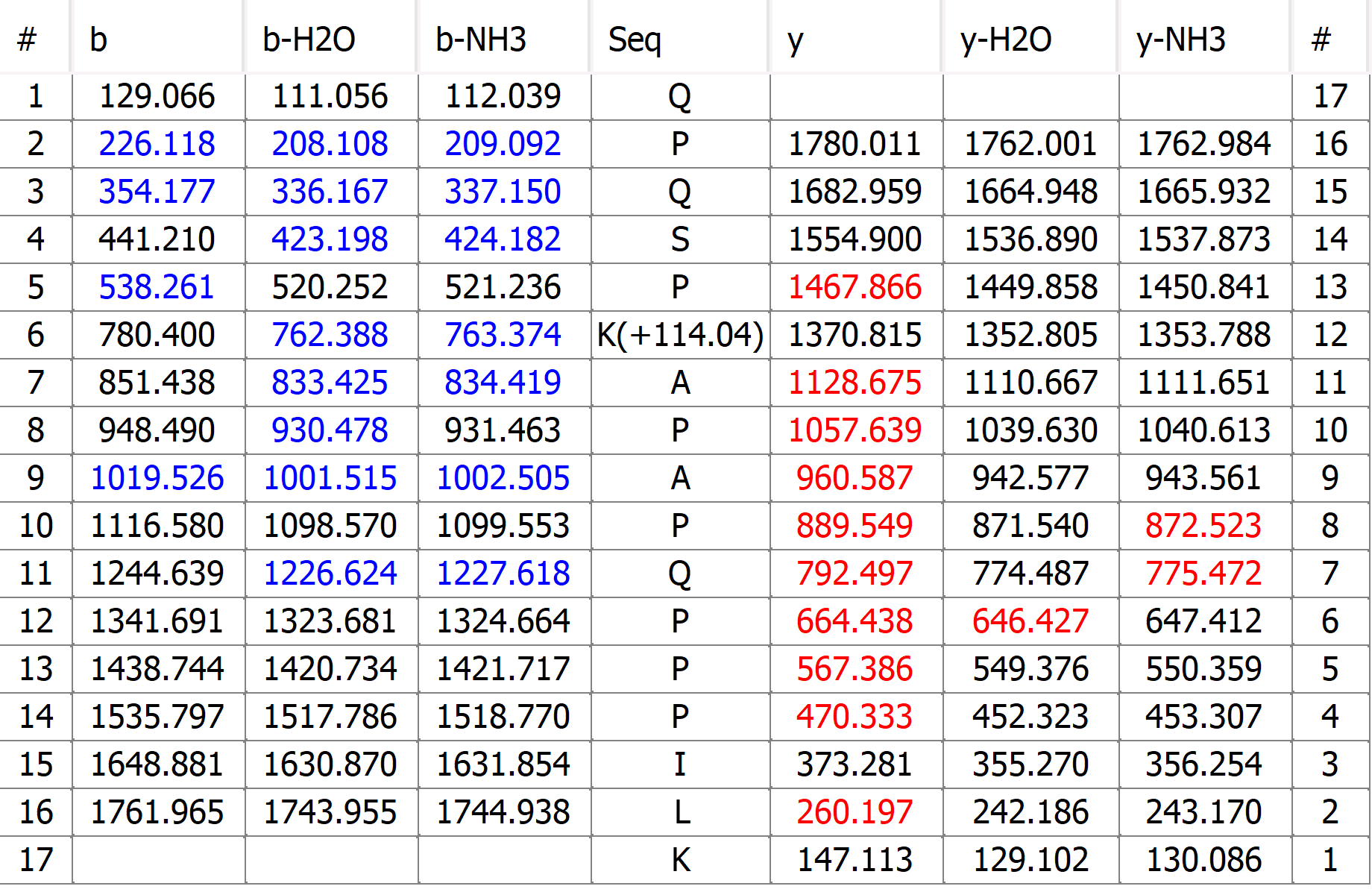
**

**Lysine 147**

**
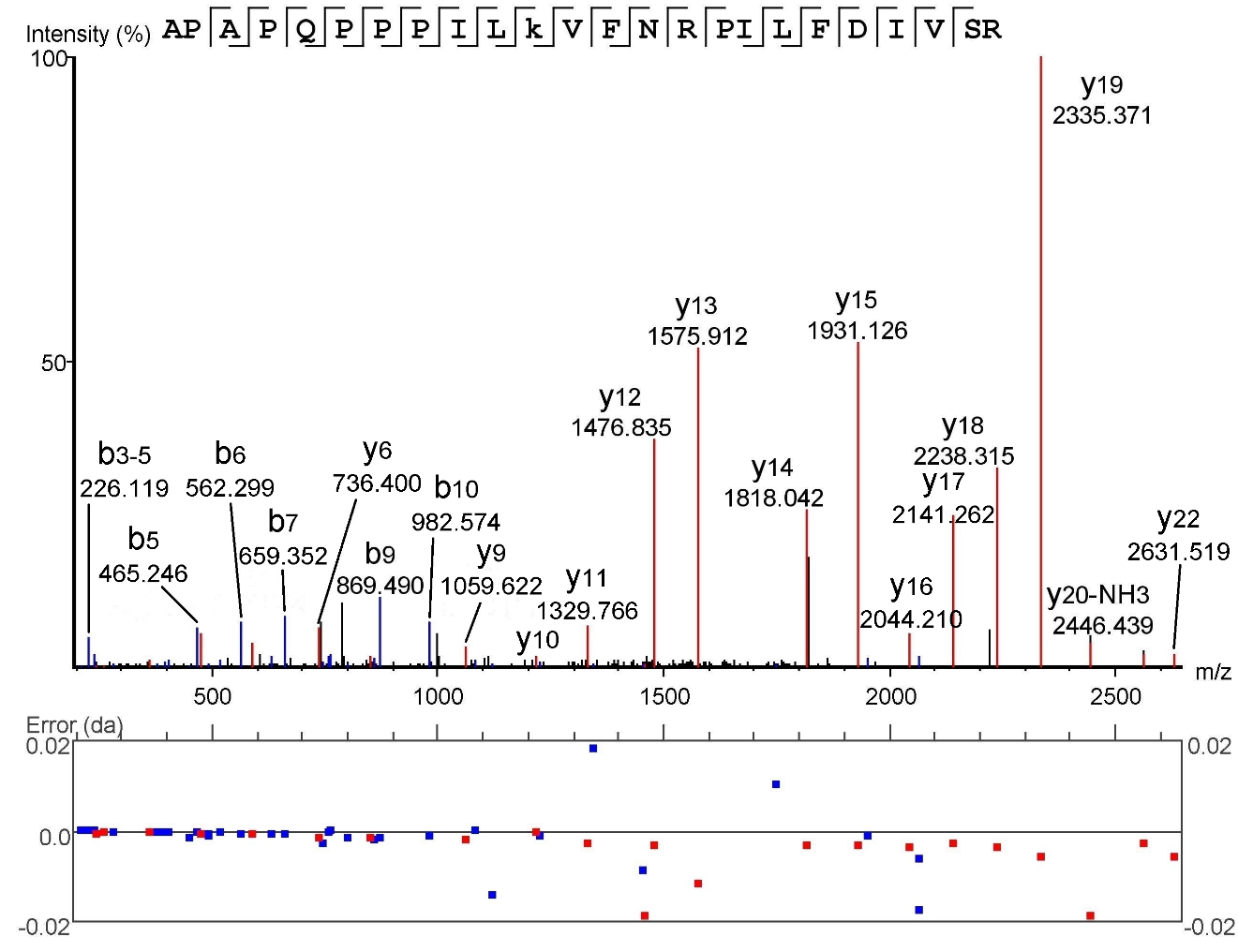
**

**
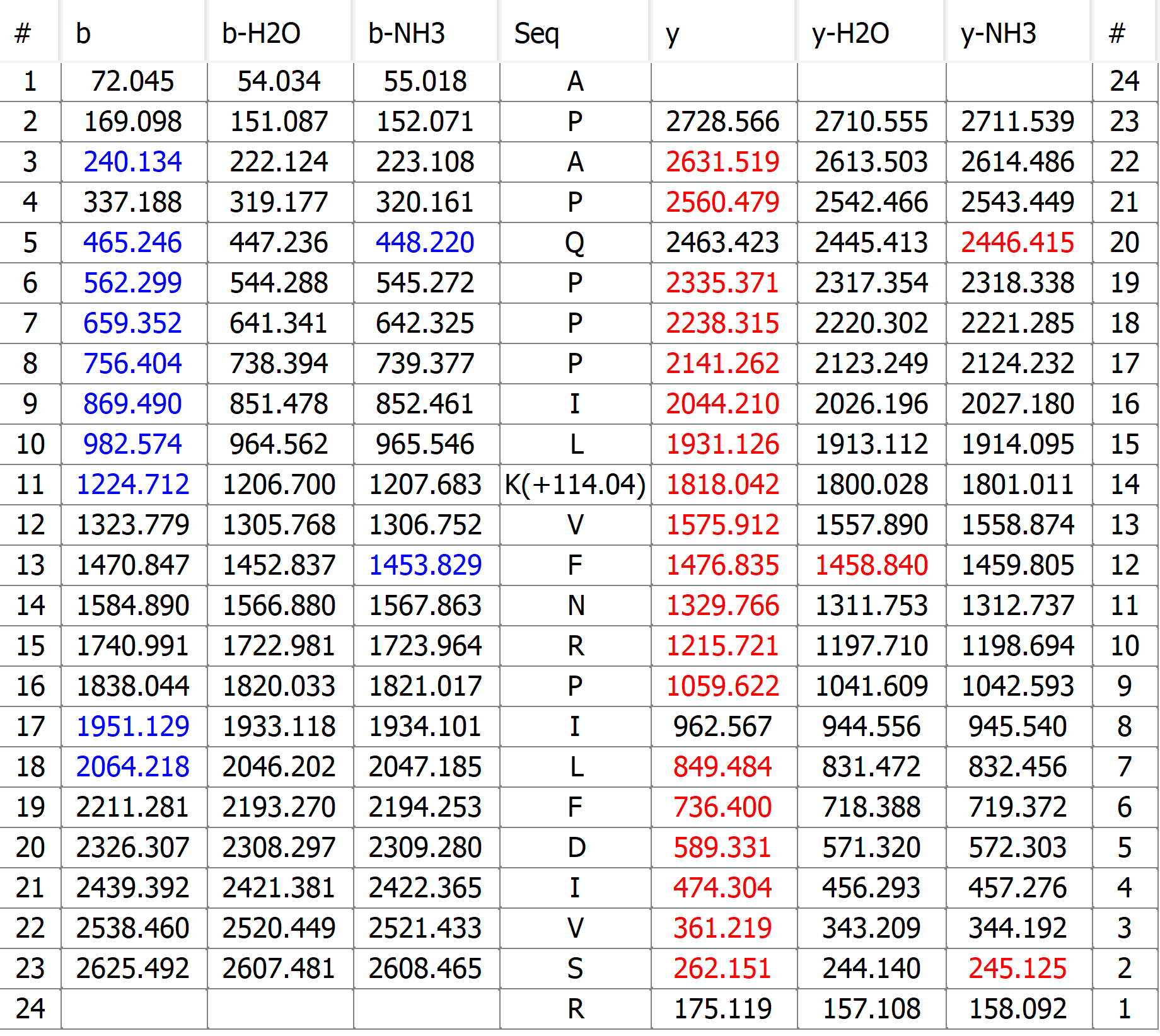
**

**Lysine 192**

**
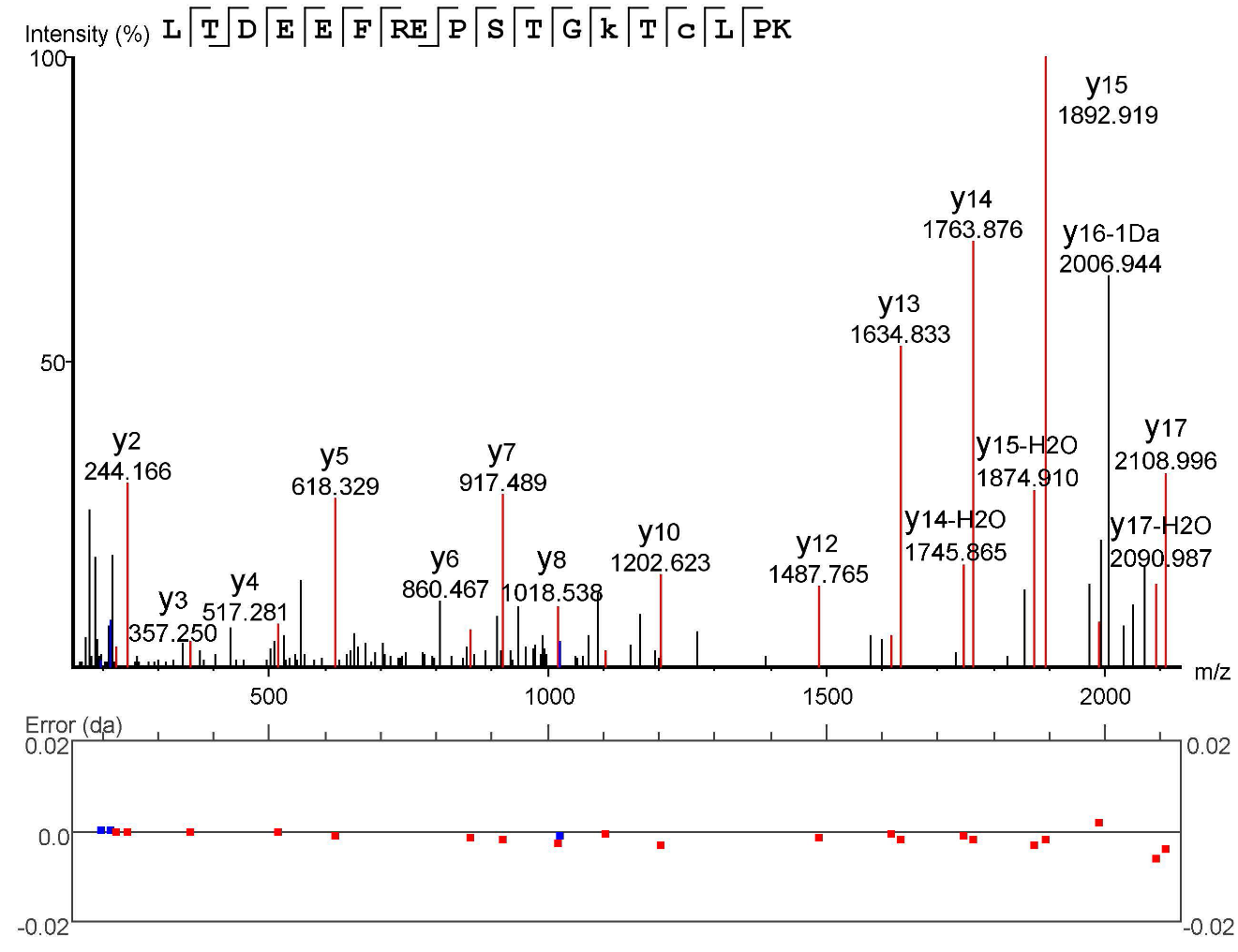
**

**
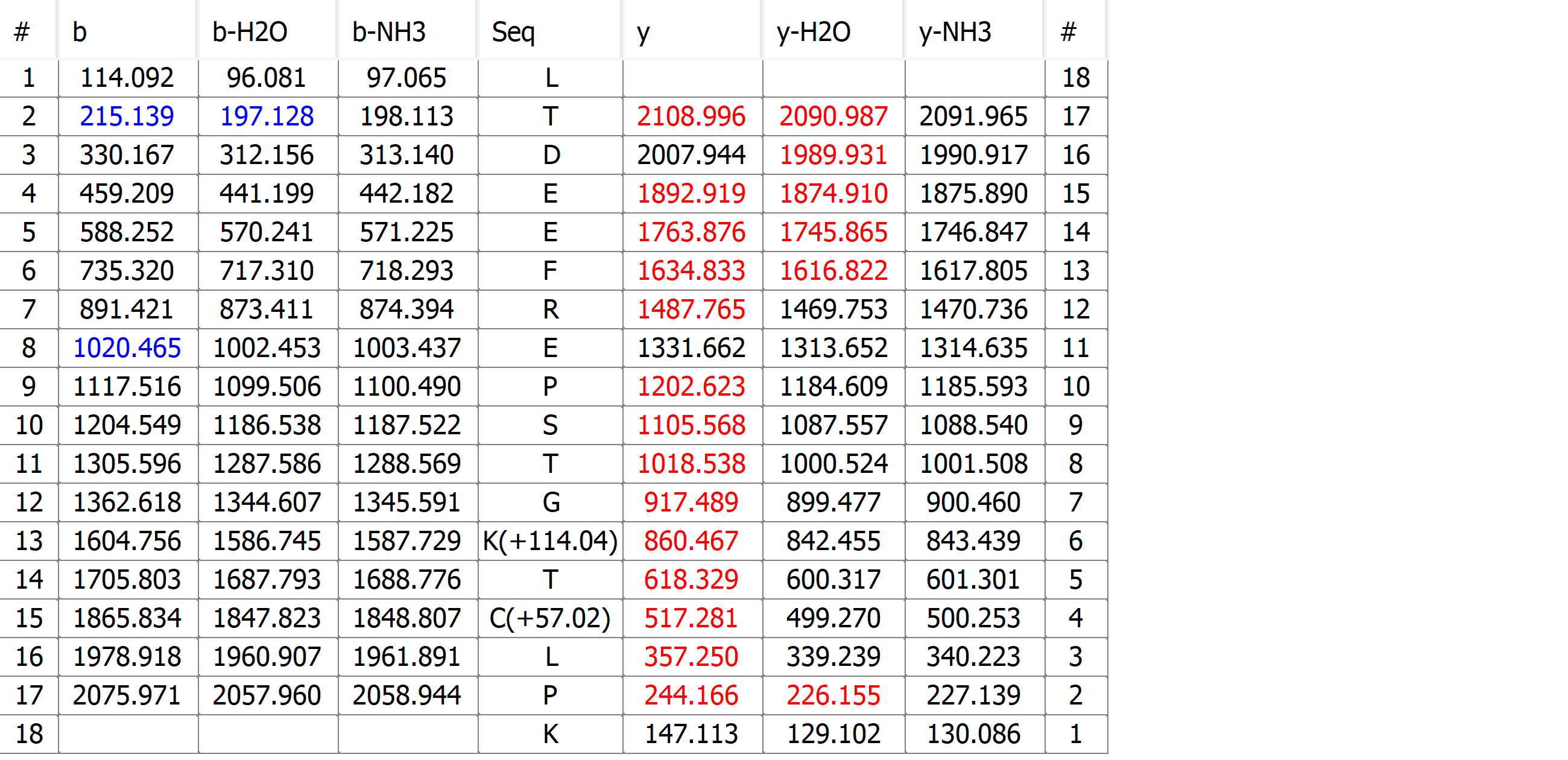
**

**Lysine 197**

**
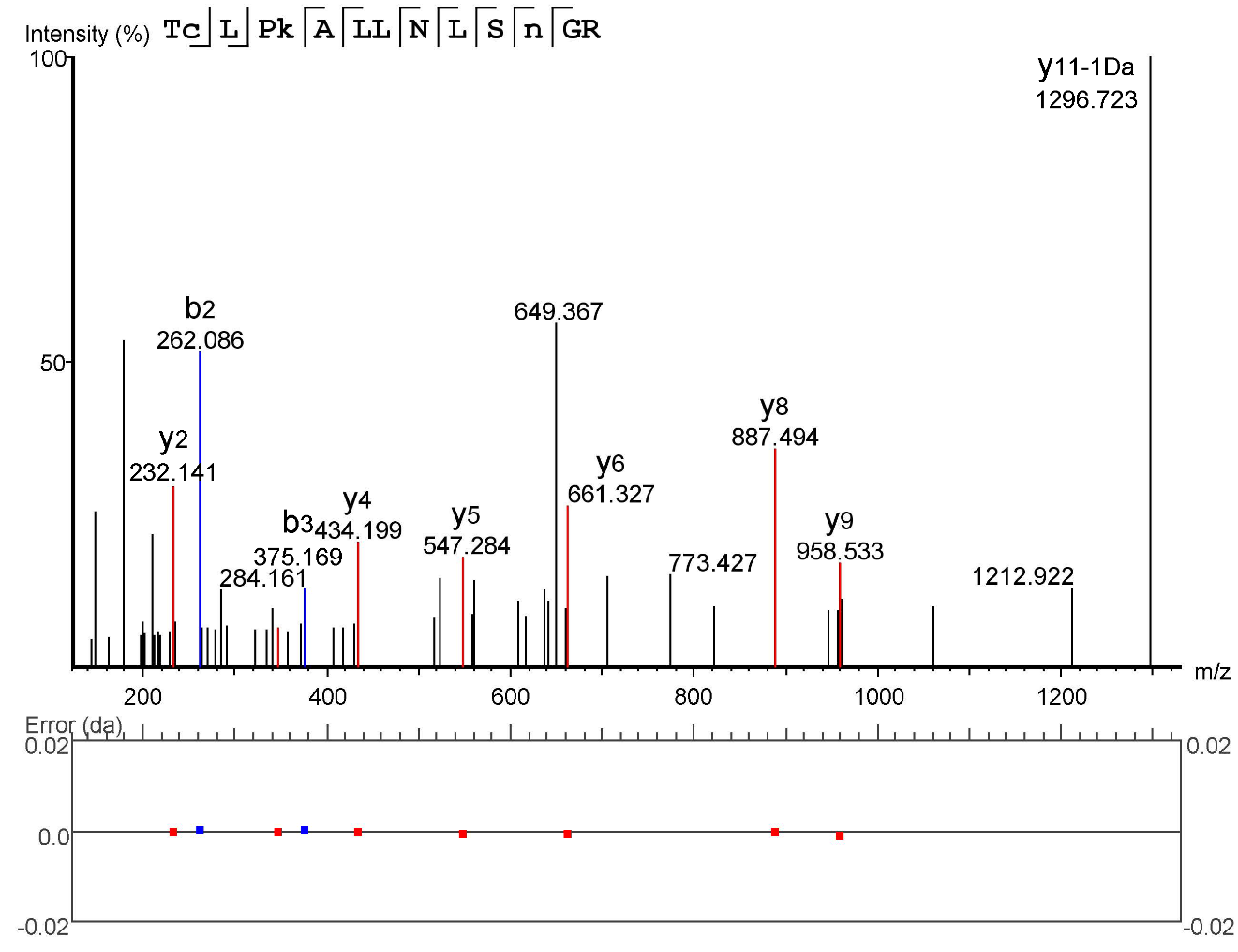
**

**
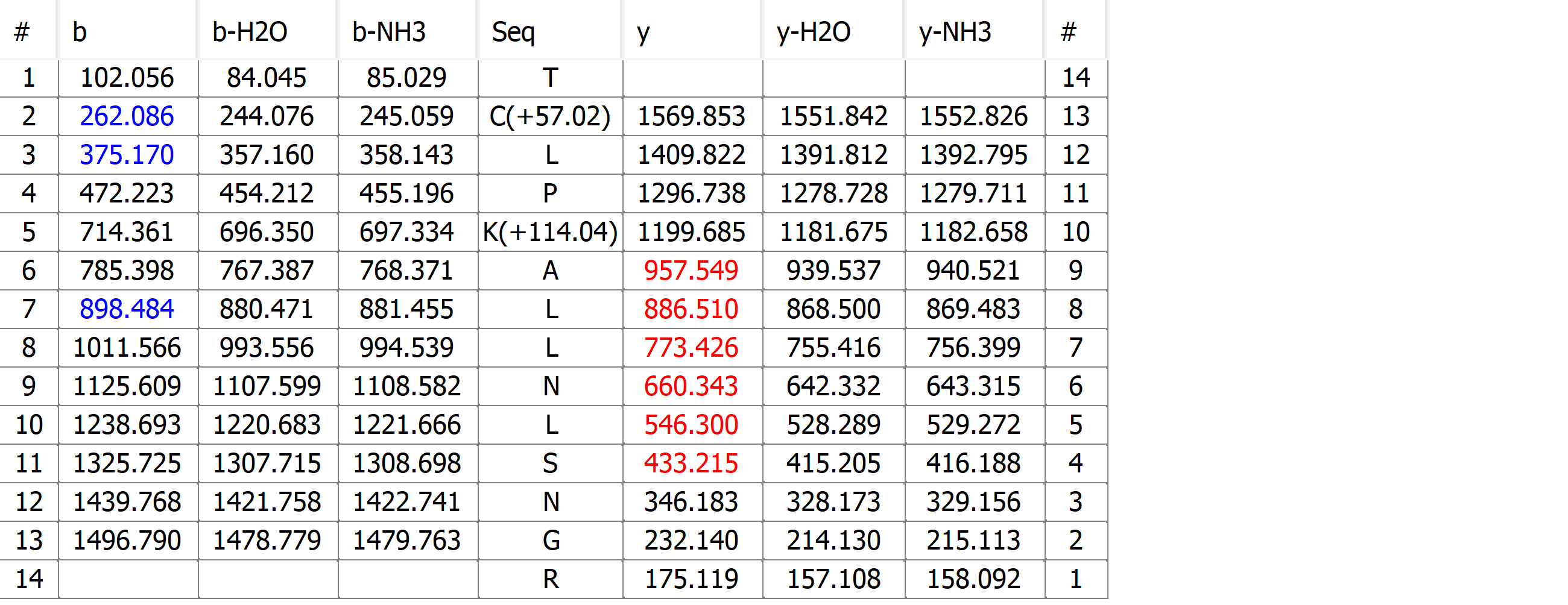
**

**Lysine 340**

**
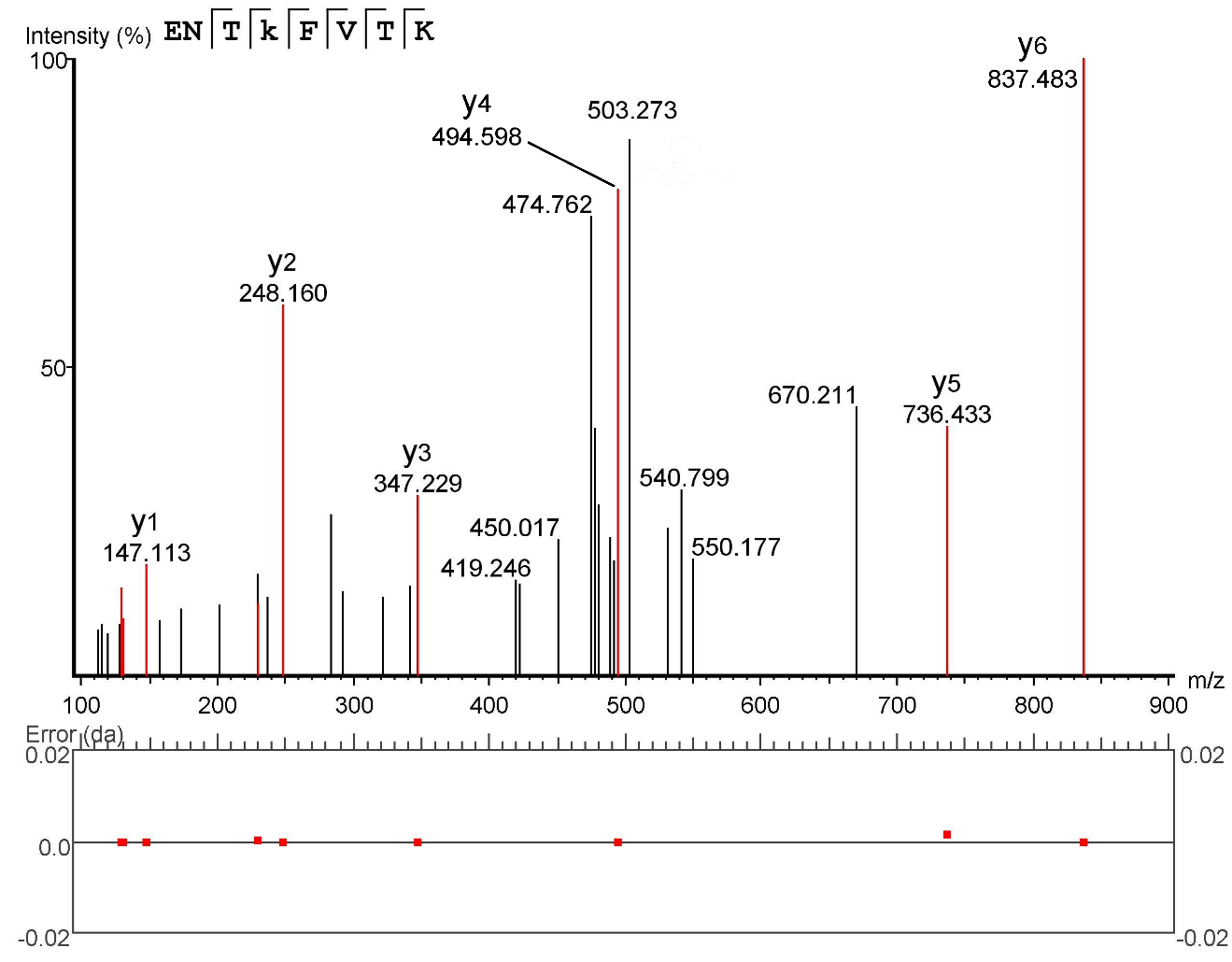
**

**
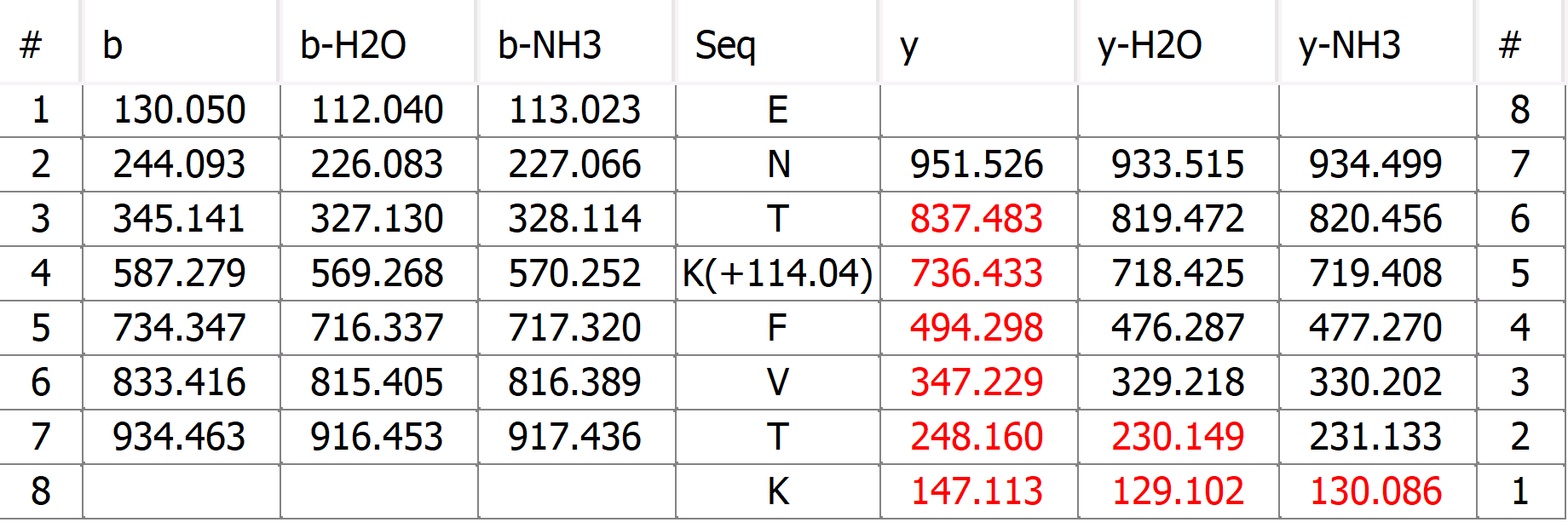
**

**Lysine 344**

**
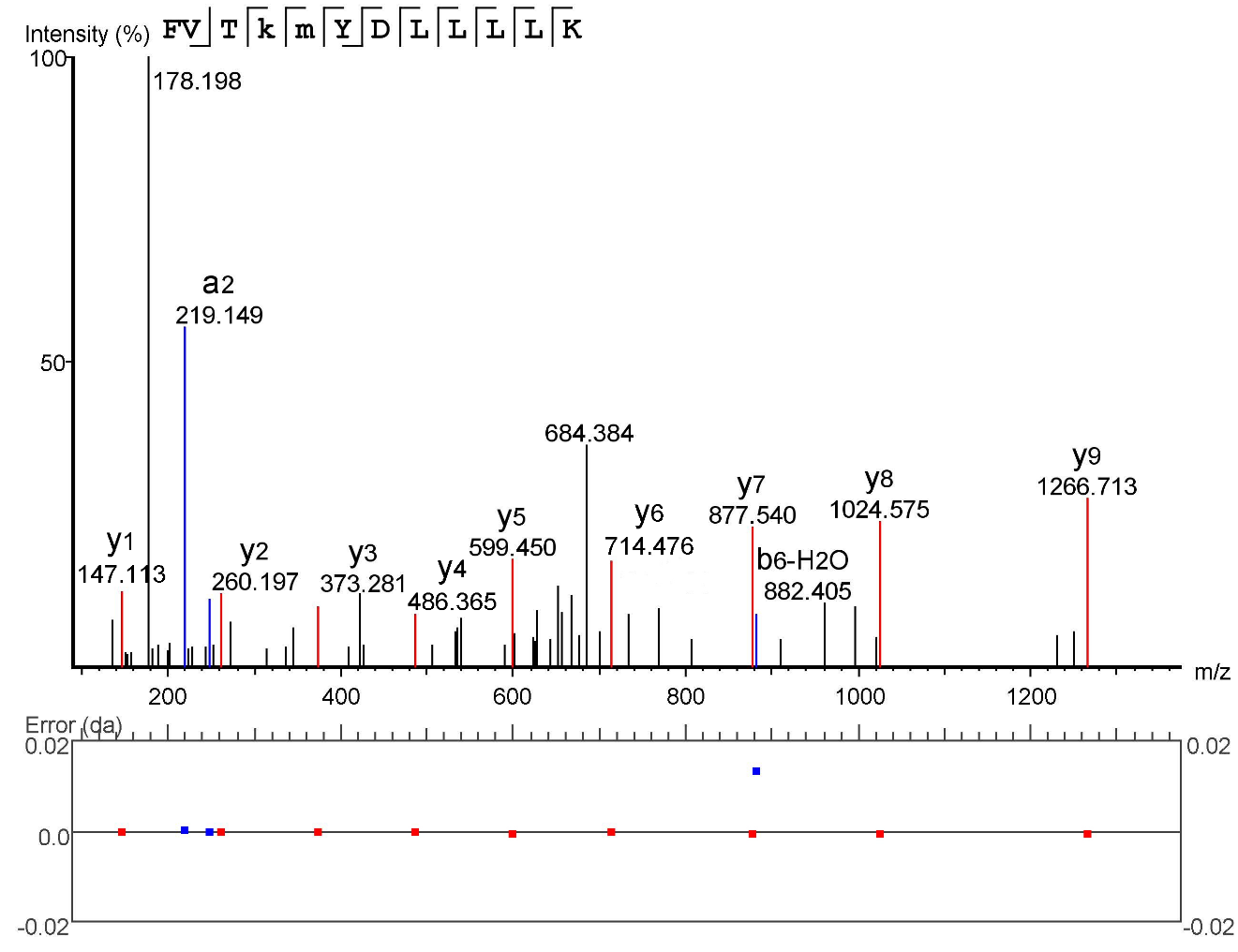
**

**
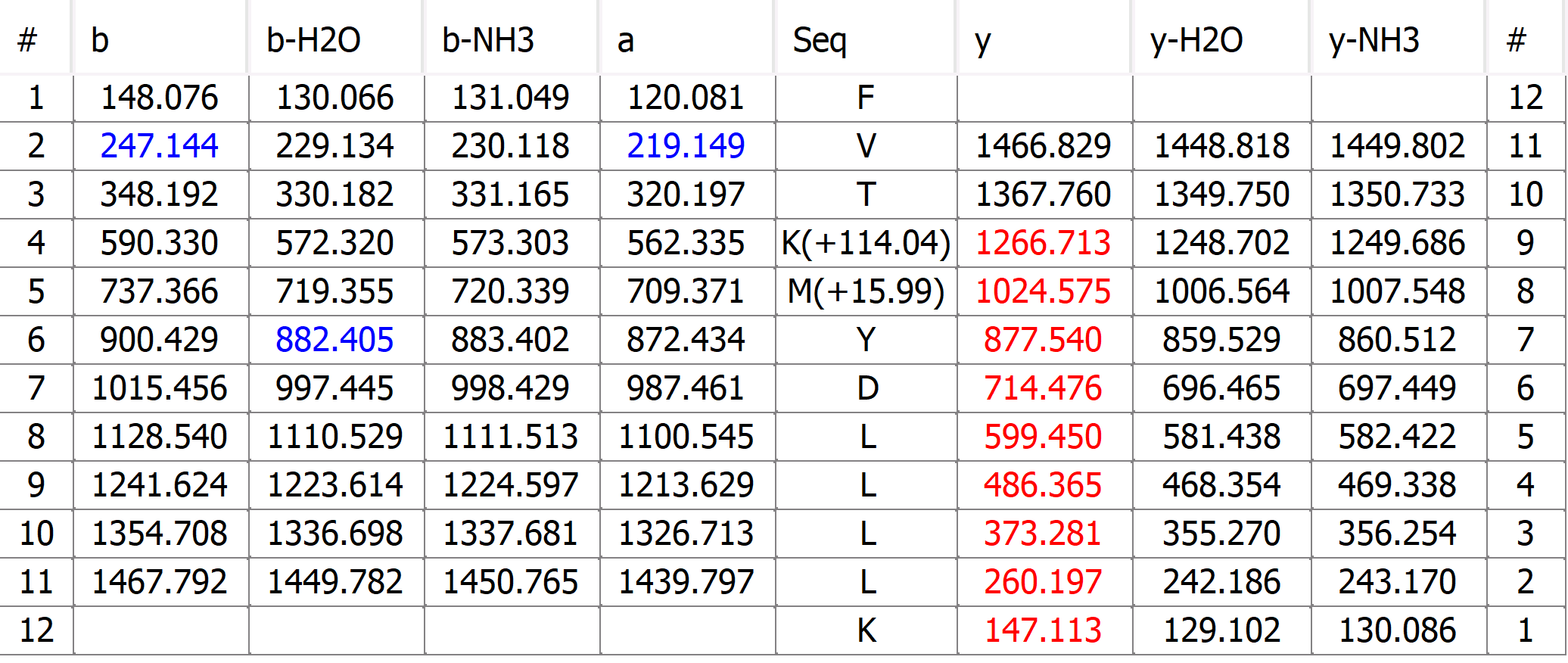
**

**Lysine 352**

**
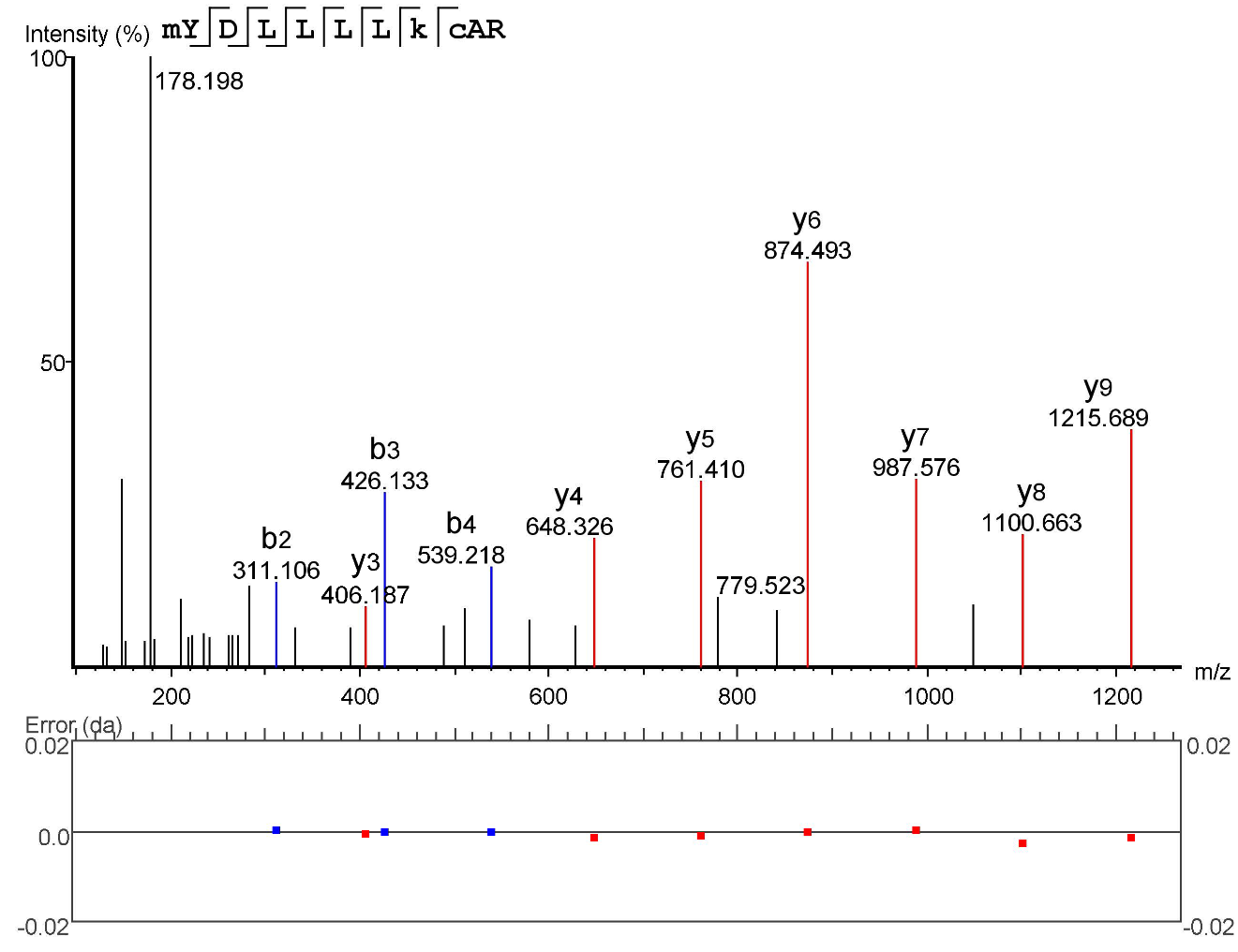
**

**
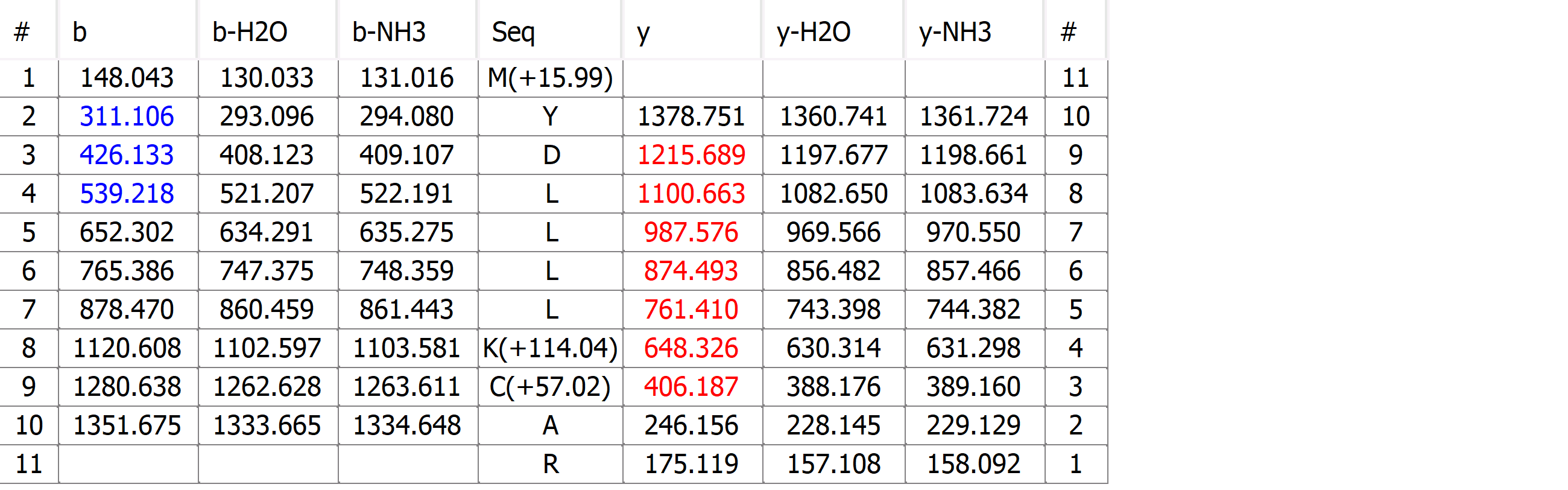
**

**Lysine 407
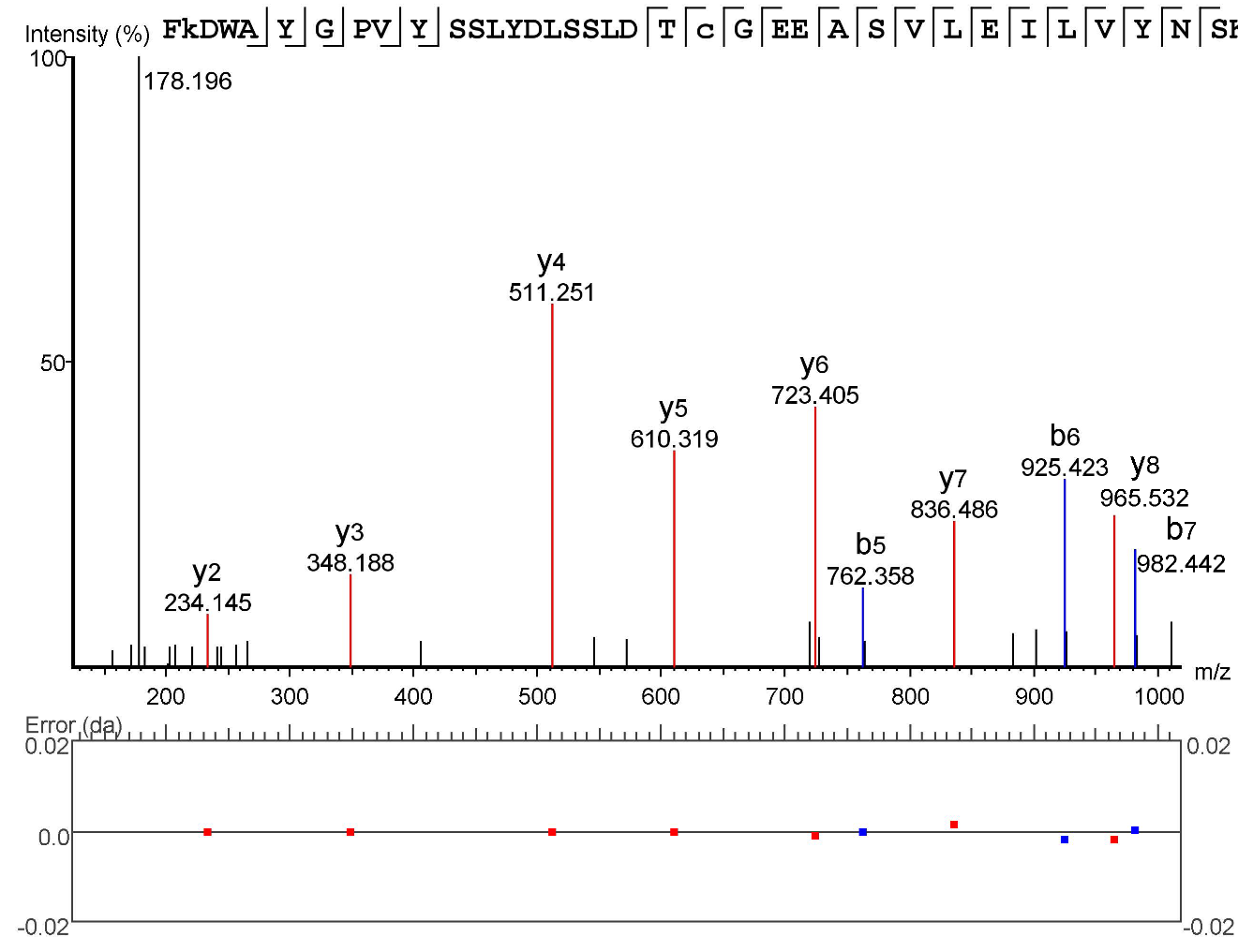
**

**
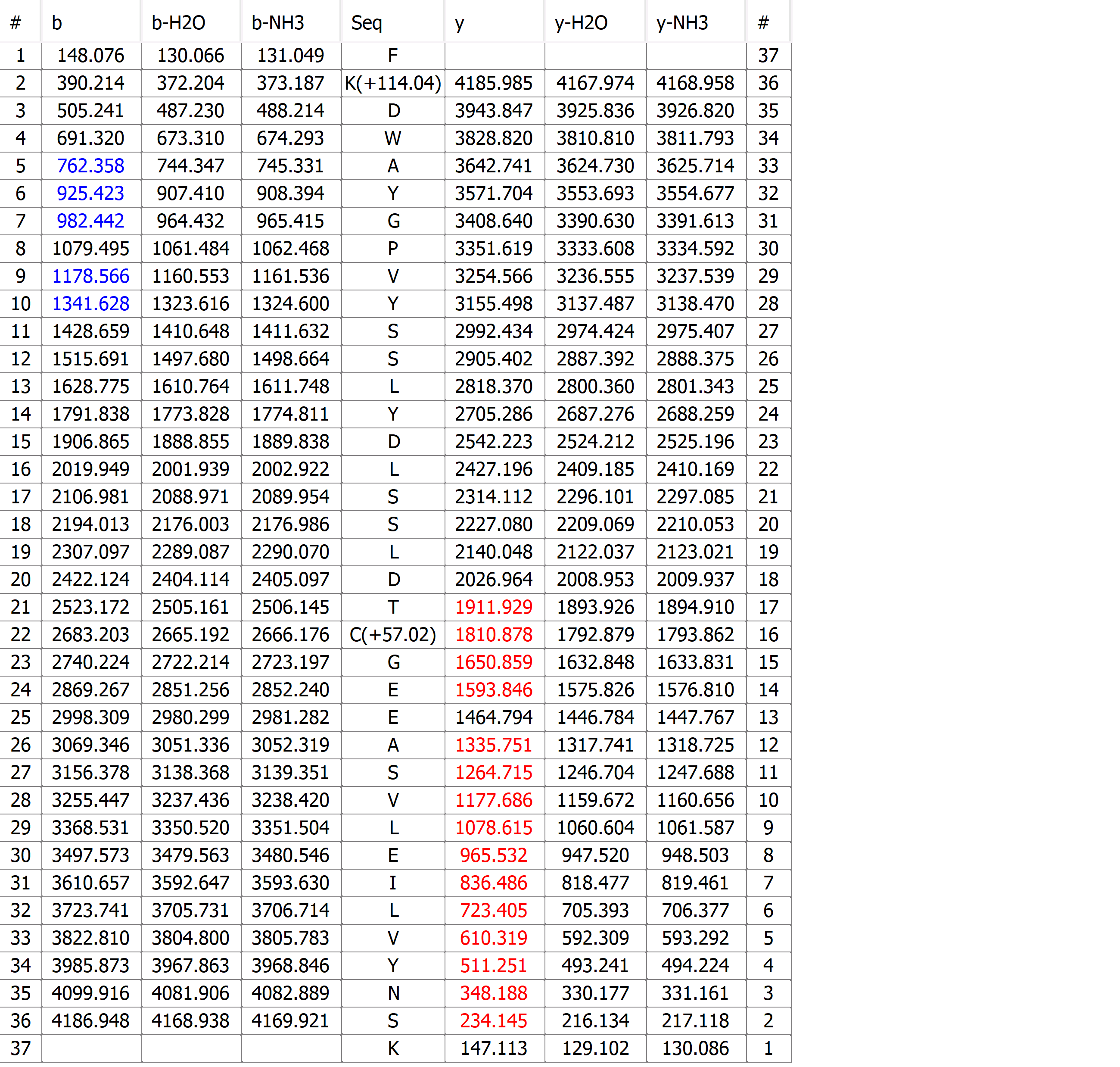
**

**Lysine 766**

**
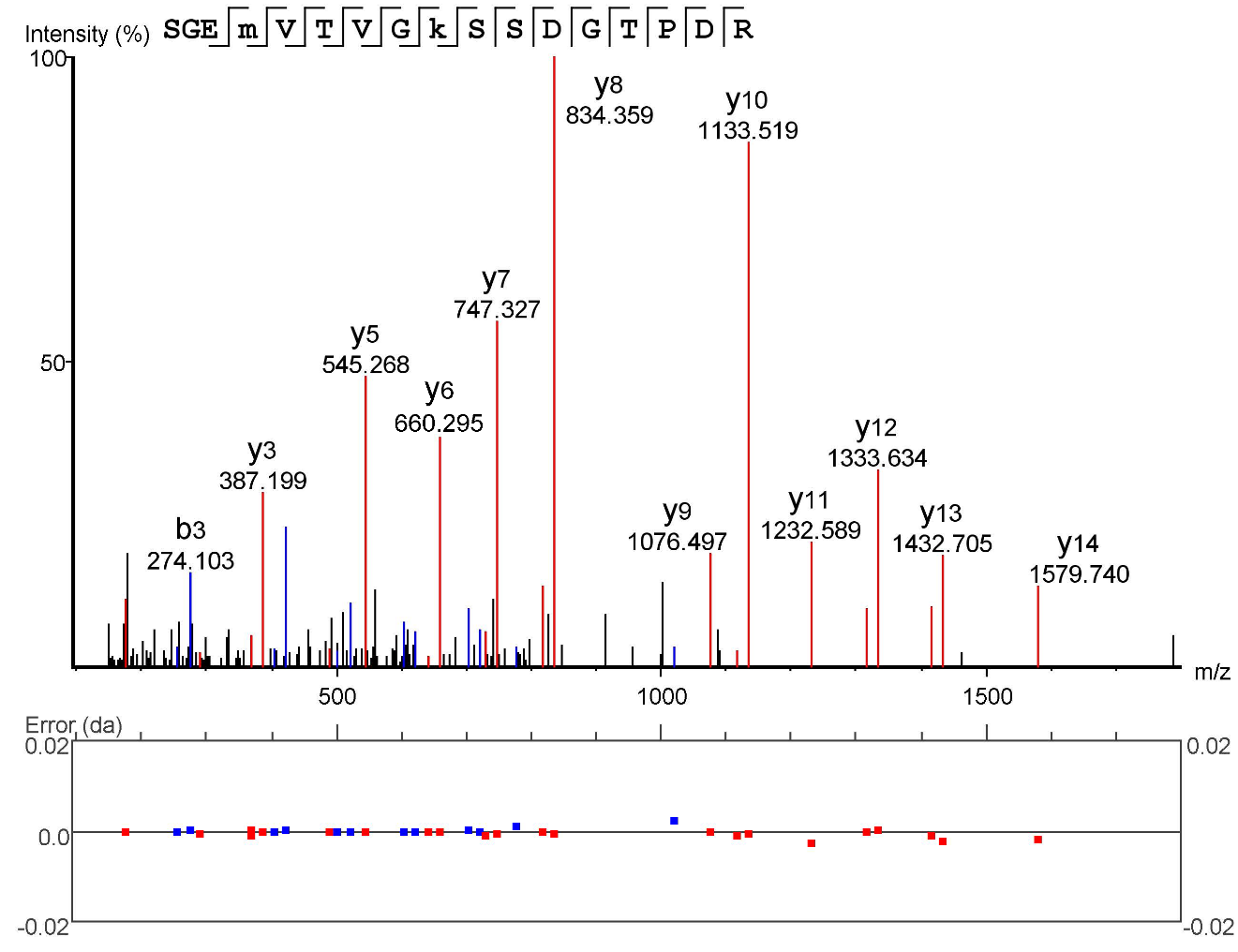
**

**
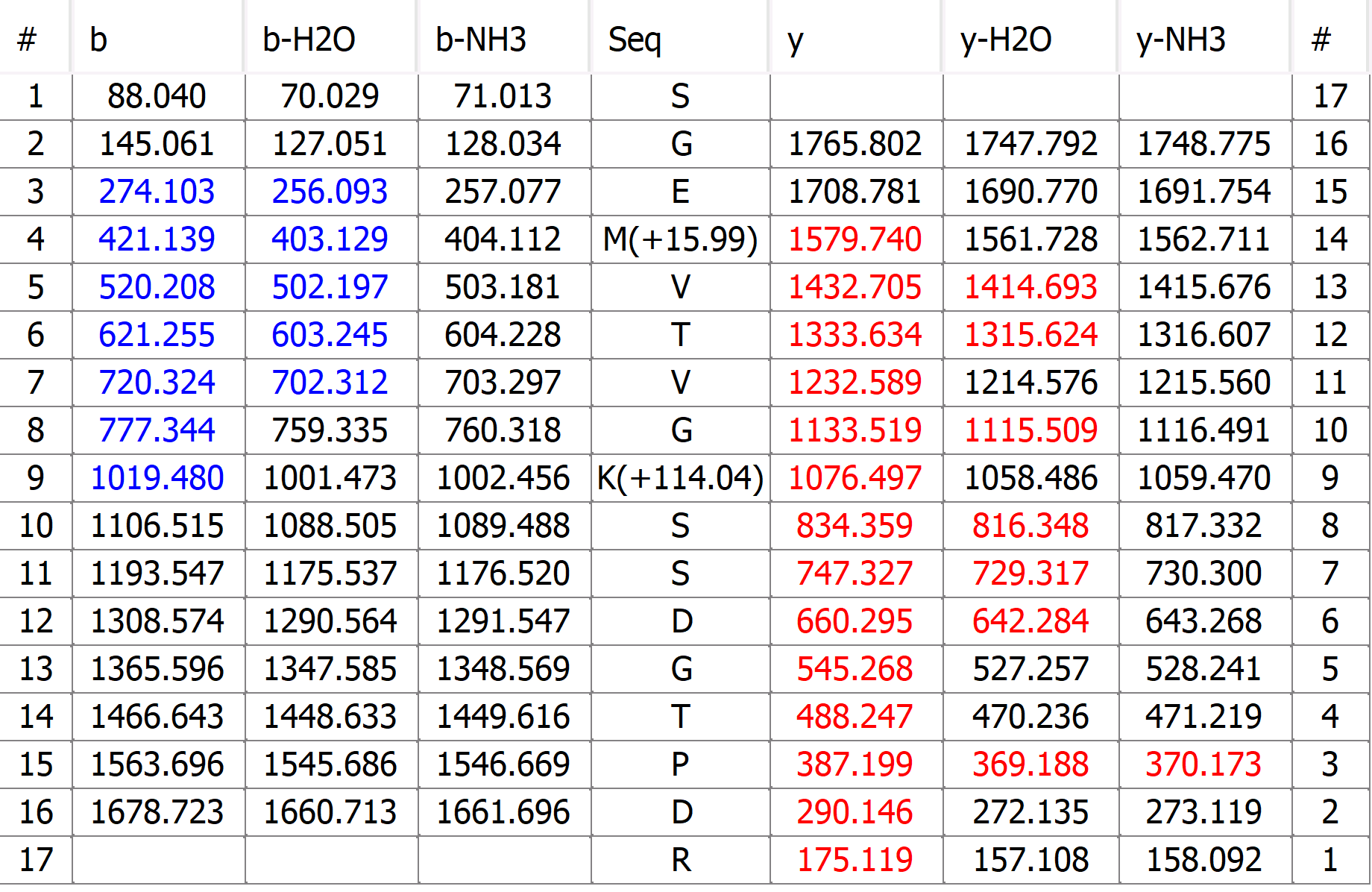
**

**Lysine 801**

**
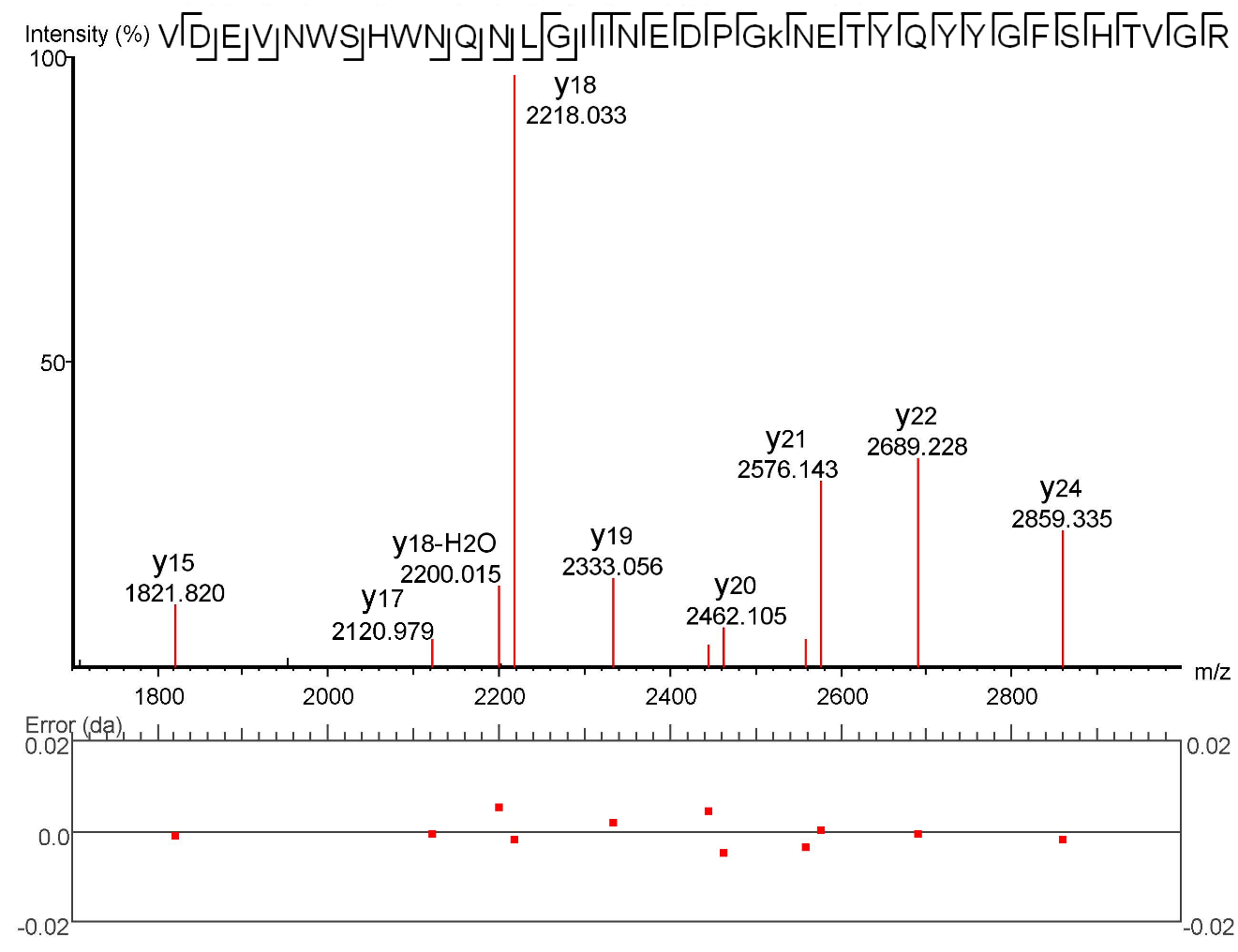
**

**
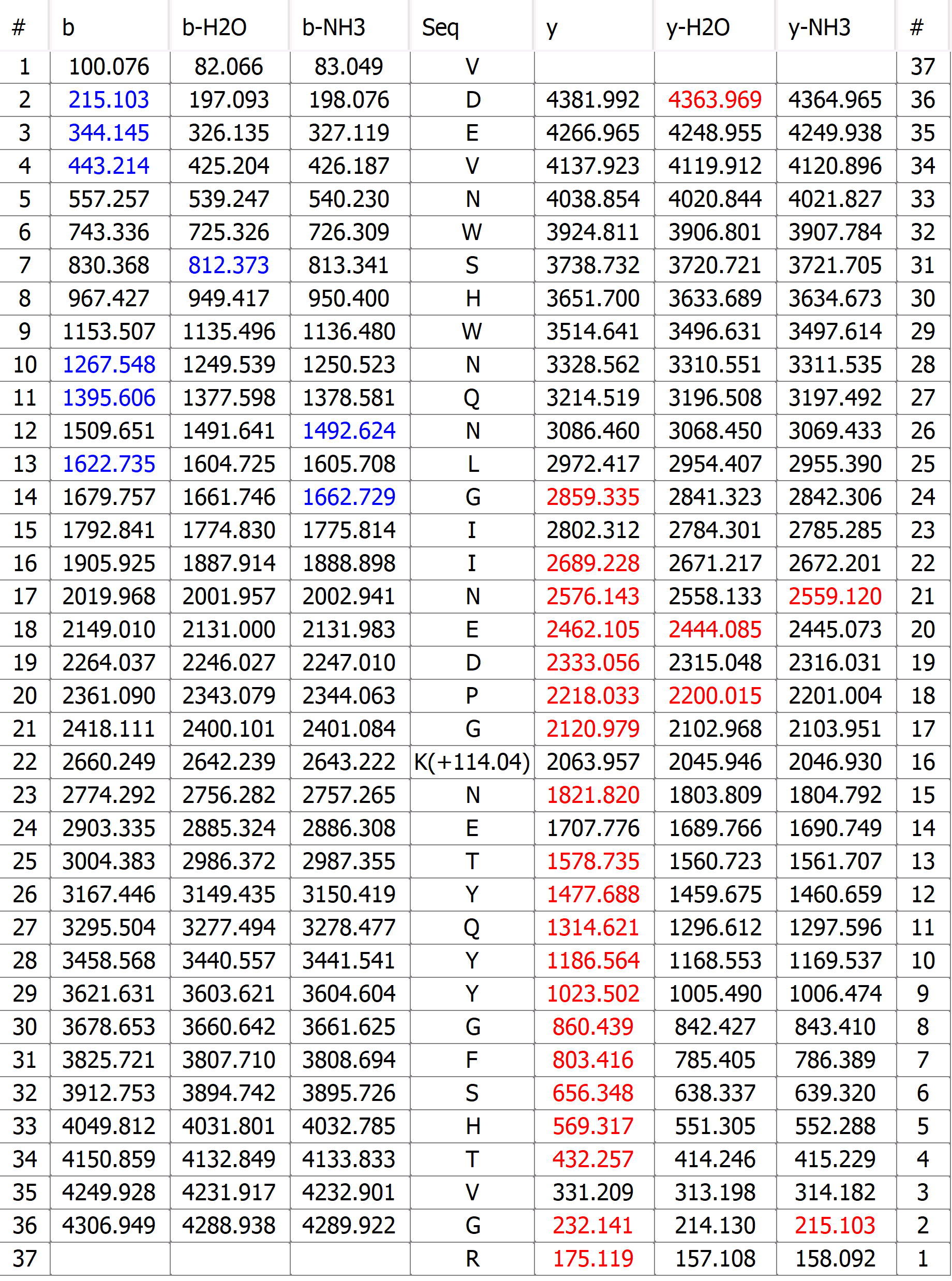
**

**Lysine 834**

**
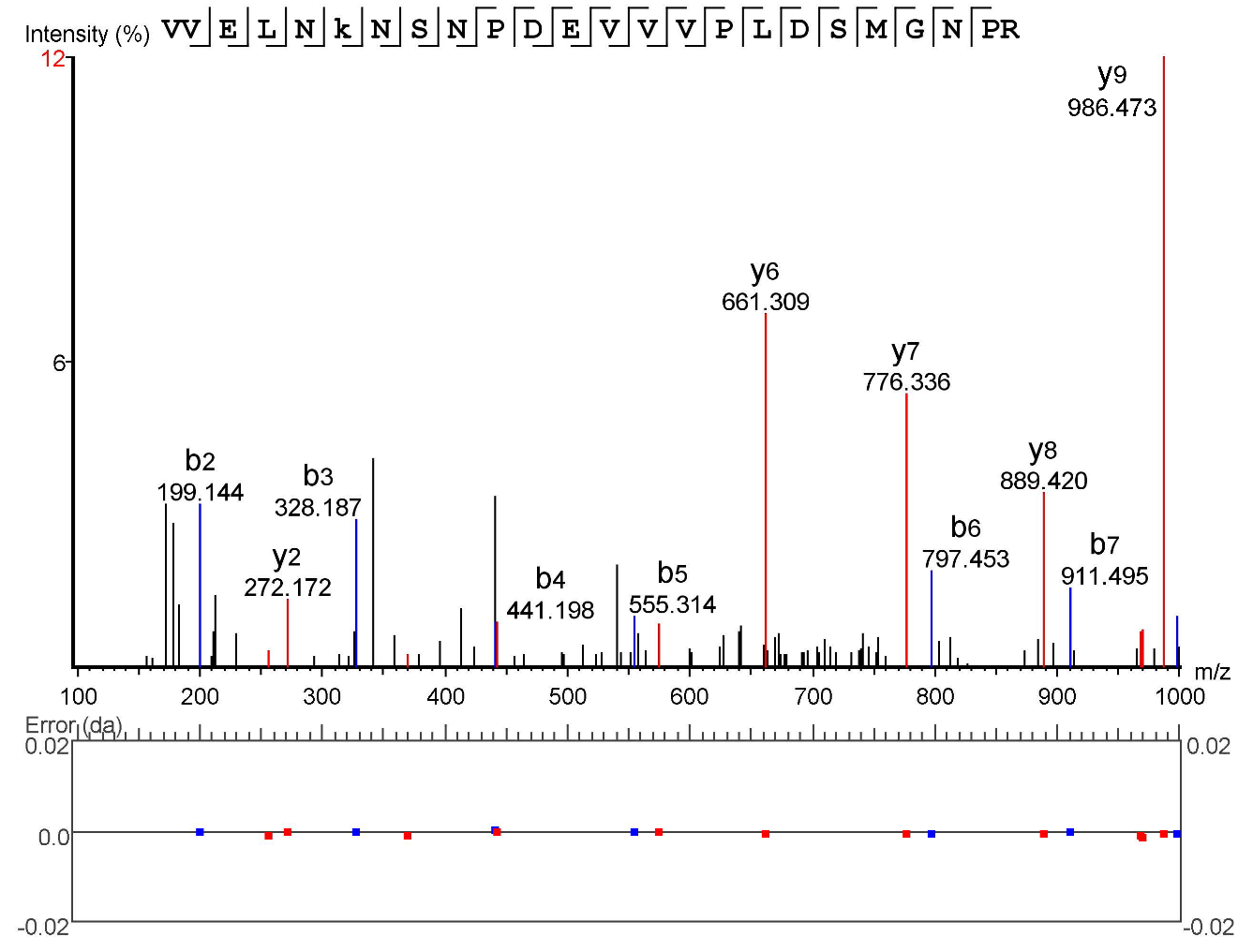
**

**
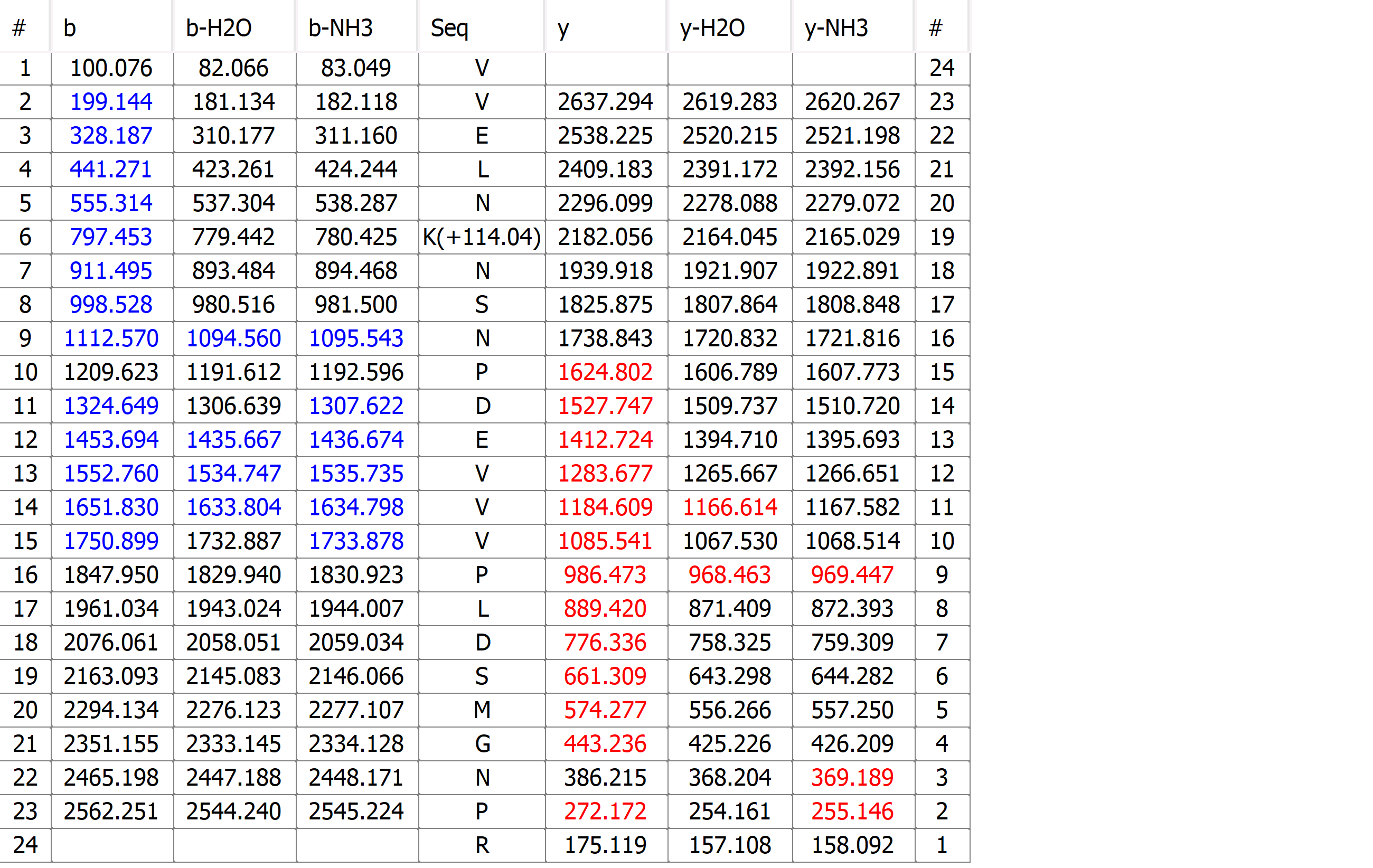
**
