# Supplementary material for: Multiubiquitination of TRPV4 reduces channel activity independent of surface localization
Source: J Biol Chem. 2022 Mar 14;298(4):101826. doi: 10.1016/j.jbc.2022.101826 (PMC9010760; doi:10.1016/j.jbc.2022.101826)
Supplement: Supplemental Figure legends [file mmc2.docx]

**Multi-ubiquitination of TRPV4 modulates channel activity independent of surface localization**

William H. Aisenberg, Brett A. McCray, Jeremy M. Sullivan, Erika Diehl, Lauren R. DeVine, Jonathan Alevy, Anna M. Bagnell, Jack K. Donohue, Patrice Carr, Benedikt Goretzki, Robert N. Cole, Ute A. Hellmich, and Charlotte J. Sumner

**Supporting material included:**

Supplemental Figure legends…………………………………………………………………...S-4-S10

Supplemental Figure 1 Legend………………………………………………………………S-2

Supplemental Figure 2 Legend………………………………………………………………S-3

Supplemental Figure 3 Legend………………………………………………………………S-4

Supplemental Figure 4 Legend………………………………………………………………S-5

Supplemental Figure 5 Legend………………………………………………………………S-6

Supplemental Figure 6 Legend………………………………………………………….…..S-8

**Supplementary Figure 1: Validation of TRPV4 antibody, and location of ubiquitin sites within the channel ARD. A)** Demonstration of the specificity of the anti-TRPV4 antibody by detection of endogenous TRPV4 in wild type (WT), but not TRPV4 knockout (KO) (42), mouse choroid plexus tissues by western blot. A ~75 kDa non-specific band in the KO tissue is also faintly visible in WT choroid plexus tissue just below the TRPV4 bands (black arrows). **B)** Lysates from whole mouse kidney demonstrating TRPV4 antibody specificity. TRPV4 bands are indicated with black arrows in WT but not KO tissues (41). **C)** Uncropped blots from Figs. 1A-C demonstrating immunoprecipitation specificity with the TRPV4 antibody. Whole cell lysates probed with TRPV4 antibody (images at left) yield true TRPV4 signal (black arrows), as well as a number of non-specific bands. Following immunoprecipitation with anti-TRPV4 antibody, the TRPV4 bands remain with minimal non-specific signal. **D)** Lysine 192 and 197 are part of an ATP-binding site (green box) within the TRPV4 ARD (PDB: 4DX2). Bound ATP is shown in yellow, with its phosphates in orange; the lysine 192 and 197 side chains (red) point where the ATP phosphate groups would go upon ATP interaction.

**Supplementary Figure 2: K>R mutagenesis of select lysines within the TRPV4 N-terminal IDR results in increased channel activity. A-B)** Cell-based ubiquitination assays examining HEK293T cells transfected with WT or K>R TRPV4 mutants and either incubated without **(A)** or with **(B)** 0.5 µM HC067. Red arrows denote ubiquitinated TRPV4. The All-K-IDR construct harbors K70, 77, 100, 101, 121, 125, 126, 130, 136, and 147R mutations; the 5K-IDR construct harbors K77, 101, 130, 136, and 147R mutations; and the 5K-CTD construct harbors K754, 768, 801, 834, and 863R mutations. **C)** Quantification of baseline intracellular calcium levels in HEK293T cells transfected with WT TRPV4 or K>R mutants. n = 29-95 cells/condition over at least three independent transfections. Data are presented as means ± SEM. *****p* < 0.0001 (one-way ANOVA with Dunnett’s post hoc test), ####*p* < 0.0001 (two-way ANOVA). **D)** Representative confocal images of unpermeabilized TRPV4-V5-EGFP-expressing HEK293T cells showing cell surface (magenta; anti-V5 antibody) and total (green; EGFP tag) TRPV4, and nuclei (blue; DAPI) in the absence or presence of HC067 (0.5 µM) for K130, 136R and All-K-IDR TRPV4. The “no V5” construct is TRPV4-EGFP without a V5 loop epitope tag – and serves as a negative control for surface staining. **E)** Quantification of the effects of HC067 on the surface density of the K130, 136R mutant. Data are presented as means ± SEM. n = 52-66 cells/condition. ns, not significant (unpaired two-tailed t test). **F)** Calcium imaging of WT and K130, 136R TRPV4-expressing HEK293T cells following antagonist wash out (vertical dotted line). GFP and WT TRPV4 traces from Fig. 3e are shown in comparison to the K130, 136R mutant. Solid lines represent means, and shading SEM. n = 25-43 cells/condition from three independent transfections.

**Supplementary Figure 3: NEDD4 family E3 ubiquitin ligases can ubiquitinate the TRPV4 N-terminal IDR. A)** Cell-based ubiquitination assays performed on HEK293T cells transfected with TRPV1-FLAG and either Myc-NEDD4 or Myc-ITCH. **B)** Linear schematic of the full N-terminal domain (NTD) of a TRPV4 monomer. Positions of individual lysines are marked with vertical lines, and sites of ITCH ubiquitination identified by mass spectrometry are marked with green circles. PBD = PIP_2_-binding domain, PRR = proline-rich region, ARD = ankyrin repeat domain. **C-E)** Coomassie Blue-stained gels showing results from cell-free ubiquitination assays run over the course of three hours **(C-D)** or one hour **(E)**, utilizing NEDD4 (**C**) or ITCH (**D-E**) as the E3 ligase (open arrowheads represent unmodified E3 ligase; red arrowheads indicate auto-ubiquitinated E3 ligase). For NEDD4 assays, the full TRPV4 NTD (~50 kD; open arrow) was examined and no mass-shifted ubiquitinated product was detected. NEDD4, however, was able to auto-ubiquitinate (red arrowhead), demonstrating the presence of functional enzyme. For the ITCH assays shown in **D**, either the TRPV4 ARD (<37 kD), full TRPV4 NTD (~50 kD; open arrows) was examined. Formation of mono-ubiquitinated TRPV4 product is indicated with the red arrow. For the ITCH assays shown in **E**, multiple limiting lengths of the TRPV4 NTD were examined, with only the full NTD exhibiting a mass-shifted ubiquitinated product (red arrow; compare white box from time 0 – right – to time 60 – left).

**Supplementary Figure 4: NEDD4 overexpression reduces calcium responses to a variety of TRPV4 activators.** **A-B)** Calcium imaging of transfected HEK293T cells expressing WT TRPV4 alone, or with either NEDD4 or NEDD4-CI, following application of 10 nM GSK101 (**A**) or hypotonic saline (HTS; **B**) at the vertical dotted lines. Solid lines represent mean and shading SEM. **C)** Quantification of the time to response following stimulation with hypotonic saline. Data are presented as means ± SD. n = 40-51 cells/condition. *****p* < 0.0001.(one-way ANOVA with Dunnett’s post hoc test) **D)** Representative cell-based ubiquitination assay performed on HEK293T cells transfected with TRPV4 K>R mutants, with and without NEDD4 co-transfection, and cultured for 24 hours in 0.5 µM HC067. **E-G)** Calcium response latency in transfected HEK293T cells cultured as in **D**, following removal of HC067 and stimulation with 10 nM GSK101. Cells were transfected with WT **(E)** 5K-IDR **(F)** or 5K-CTD **(G)** TRPV4, either with or without NEDD4 co-transfection. Data are presented as means ± SD. *****p* < 0.0001 (**E**, n = 51-69 cells from three independent coverslips, unpaired two tailed t test; **F**, n = 72-100 cells from three independent coverslips, Mann-Whitney test; **G**, n = 52-62 cells from three independent coverslips, Mann-Whitney test).

**Supplementary Figure 5: NEDD4 expression does not reduce TRPV4 surface abundance. A)** Flow cytometry analysis of TRPV4 surface expression in HEK293T cells transfected with either empty vector or TRPV4-V5-FLAG, with or without NEDD4 co-transfection. Cell surface TRPV4 was stained by incubating cells with DyLight 488-conjugated anti-V5 antibody. Top panels show representative flow plots for each condition, with the percentage of stained cells within the gated population indicated. Fluorescence intensity of the gated cells is shown in the lower panels, with mean fluorescence intensity indicated below each histogram. **B-C)** Quantification of cell surface biotinylation assays from cells shown in Fig. 6C-D in which HEK293T cells were transfected with either TRPV4 alone **(B)** or with co-expression of NEDD4 **(C).** Surface/total ratios were calculated by densitometry in FIJI, where band intensities for both surface and total fractions were normalized to their respective time 0. A surface/total ratio was then calculated. Data are presented as means ± SD. n = 5 (B) and n = 6 (C) independent experiments. Kruskal-Wallis test with Dunn’s multiple comparison test showed no significant difference between time points. **D)** Western blot analysis of cell-surface biotinylation assays performed using transfected HEK293T cells. Whole cell lysates were run on the left half of the blot, with corresponding surface (biotin) fractions run at right. Immunodetection of the transferrin receptor (TfR) was performed as a control for loading of the surface fraction, while immunodetection for actin was performed to validate that the surface fraction was not contaminated by cytosolic proteins. **E)** Densitometry-based quantification of the cell-surface biotinylation assays shown in **D**. TRPV4 surface expression was normalized to the surface expression of the TfR loading control for each sample. Similarly, whole-cell TRPV4-FLAG was normalized to whole-cell TfR expression, and the ratio of normalized surface-to-total TRPV4 then calculated. n = 4 independent experiments. RM analysis by one-way ANOVA showed no significant differences between WT TRPV4 alone and any other condition. **F)** Quantification of response latency for the calcium imaging experiments shown in Fig. 6I-L, examining the effects of NEDD4 co-expression on WT TRPV4 responses to 10 nM GSK101. Data are presented as means ± SD. n = 7-8 cells/condition from 4 independent experiments. ****p* = 0.0008. unpaired two-tailed t test. **G)** Quantification of maximum intracellular calcium levels in HEK239T cells transfected with WT TRPV4 and either PACSIN1-3, following application of arachidonic acid (10 µM). Data are presented as means ± SD. n = 37-44 cells/condition from two independent coverslips. ****p < 0.0001, ****p* = 0003 (one-way ANOVA with Dunnett’s post hoc test). **H**) Western blot analysis of co-immunoprecipitation experiments in HEK293T cells co-transfected with FLAG-tagged TRPV4 K>R mutants and Myc-tagged PACSIN1.**I)** Quantification of co-immunoprecipitation experiments from **H**, where immunoprecipitated PACSIN1 was normalized to total PACSIN1 from input blots. Data are represented as means ± SD**.** n = 4 independent experiments. ****p < 0.0001, and ***p = 0.0002. One way ANOVA with Sidak’s multiple comparisons test. **J)** Western blot analysis of co-immunoprecipitation experiments in HEK293T cells co-transfected with RhoA-Myc and either WT, 5K-IDR or 5K-CTD TRPV4, demonstrating that TRPV4 K>R mutants do not exhibit altered RhoA interactions.

**Supplementary Figure 6: The gain-of-channel function characteristic of neuropathy-causing TRPV4 mutants correlates with reduced channel ubiquitination. A)** Baseline intracellular calcium levels at 18 hours post-induction in T-Rex-TRPV4 cells expressing WT or R269C TRPV4. Data are presented as means ± SEM. n = 13 independent coverslips/condition. *****p* < 0.0001. (unpaired two tailed t test.) **B)** Representative cell-based ubiquitination assay utilizing T-Rex-TRPV4 cells 18 hours after induction of either WT or R269C TRPV4 expression. **C)** Quantification of the maximum increase in intracellular calcium levels of transfected MN-1 cells expressing WT or R237L TRPV4, in the presence or absence of NEDD4, following application of 10 nM GSK101. Data are presented as means ± SEM. n = 59-60 cells/condition from two independent experiments. *****p* < 0.0001 (unpaired two-tailed t test).
