# Supplementary material for: Multiubiquitination of TRPV4 reduces channel activity independent of surface localization
Source: J Biol Chem. 2022 Mar 14;298(4):101826. doi: 10.1016/j.jbc.2022.101826 (PMC9010760; doi:10.1016/j.jbc.2022.101826)
Supplement: Supplemental Table S1 [file mmc3.docx]

**Table S1**

**Ubiquitinated lysines (in red) identified on TRPV4**

| **TRPV4 functional domain** | **Lysine** | **Peptide** | **Number of Peptide Spectral Matches (PSMs) in each run** | | | | | | | | **Total PSMs** |
| --- | --- | --- | --- | --- | --- | --- | --- | --- | --- | --- | --- |
|  |  | Alkylating reagent used 🡪 | mmts | mmts | iodo | iodo | iodo | chlor | chlor | chlor |  |
| IDR | 70 | [R].M**K**FQGAFR.[K] |  | 4 |  |  |  |  | 4 | 2 | **10** |
|  | 77 | [R].**K**GVPNPIDLLESTLYESSVVPGPK.[K] | 2 |  | 2 | 1 |  |  |  |  | **5** |
|  | 101 | [K].**K**APMDSLFDYGTYR.[H] | 3 | 6 | 3 | 3 |  |  | 6 | 9 | **30** |
|  | 130 | [K].IIE**K**QPQSPK.[A] |  | 4 | 4 | 4 | 2 | 5 | 4 | 10 | **33** |
|  | 136 | [K].QPQSP**K**APAPQPPPILK.[V] | 1 |  | 3 | 1 | 1 |  |  | 8 | **14** |
| ARD | 192 | [R].LTDEEFREPST**G**KTCLPK.[A] |  |  |  | 2 | 2 |  |  |  | **4** |
|  | 197 | [K].TCLP**K**ALLNLSNGR.[N] |  |  |  |  | 4 |  |  | 3 | **7** |
|  | 340 | [R].ENT**K**FVTK.[M ] |  | 2 |  |  |  |  | 2 |  | **4** |
|  | 344 | [K].FVT**K**MYDLLLLK.[C] |  |  |  |  | 3 |  |  | 5 | **8** |
|  | 352 | [K].MYDLLLL**K**CAR.[L] |  |  |  | 1 | 6 |  |  | 8 | **15** |
|  | 382 | [K].TG**K**IGIFQHIIR.[R] |  |  |  |  | 1 |  |  |  | **1** |
|  | 407 | [K].F**K**DWAYGPVYSSLYDLSSLDTCGEEASVLEILVYNSK.[I] |  |  |  |  |  |  |  | 1 | **1** |
| CTD | 766 | [R].SGEMVTVG**K**SSDGTPDR.[R] |  | 2 | 1 |  | 2 | 1 | 2 | 3 | **11** |
|  |  | [R].SGEMVTVG**K**SSDGTPDRR.[W] | 1 | 3 | 1 | 4 | 4 |  | 3 | 8 | **24** |
|  | 801 | [R].VDEVNWSHWNQNLGIINEDPG**K**.[N] |  |  |  |  | 1 |  |  |  | **1** |
|  |  | [R].VDEVNWSHWNQNLGIINEDPG**K**NETYQYYGFSHTVGR.[L] |  |  |  |  |  |  |  | 1 | **1** |
|  | 834 | [R].VVELN**K**NSNPDEVVVPLDSMGNPR.[C] | 2 | 4 | 1 | 2 | 2 |  | 5 | 6 | **21** |
|  |  | [R].VVELN**K**.[N] |  | 2 |  |  |  |  | 3 |  | **5** |

Alkylating reagents: mmts = 20 mM methyl methanethiosulfonate, iodo = 200mM iodoacemtamide, chlor = 200mM chloroacetamide.
