# Supplementary material for: Multiubiquitination of TRPV4 reduces channel activity independent of surface localization
Source: J Biol Chem. 2022 Mar 14;298(4):101826. doi: 10.1016/j.jbc.2022.101826 (PMC9010760; doi:10.1016/j.jbc.2022.101826)
Supplement: Supplemental Table S2 [file mmc4.docx]

**Table S2**

**Ubiquitinated lysines (in red) identified on recombinant TRPV4 Full NTD from ITCH cell free ubiquitin assays**

| **TRPV4 functional domain** | **Lysine** | **Peptide** | **Percentage of runs peptide was identified** | | |
| --- | --- | --- | --- | --- | --- |
|  |  | Assay duration 🡪 | 1 hour | 1.5 hours | 3 hours |
| IDR | 77 | [R].**K**GVPNPIDLLESTLYESSVVPGPK.[K] | 100% (2/2) | 100% (2/2) | 100% (1/1) |
|  | 101 | [K].**K**APMDSLFDYGTYR.[H] | 100% (2/2) | 100% (2/2) | 100% (1/1) |
|  |  | [R].KGVPNPIDLLESTLYESSVVPGPK**K**.[A] |  |  |  |
|  | 130 | [K].IIE**K**QPQSPK.[A] |  | 50% (1/2) | 100% (1/1) |
|  | 136 | [K].QPQSP**K**APAPQPPPILK.[V] |  | 50% (1/2) | 100% (1/1) |
|  | 147 | [K].APAPQPPPIL**K**VFNRPILFDIVSR.[G] |  |  | 100% (1/1) |
| ARD | 178 | [R].GSTADLDGLLPFLLTHK**K**.[R] |  |  | 100% (1/1) |
|  | 379 | [R].LFPDSNLEAVLNNDGLSPLMMAA**K**TGK.[I] |  |  | 100% (1/1) |
