# Supplementary figures and images for: Multiubiquitination of TRPV4 reduces channel activity independent of surface localization
Source: J Biol Chem. 2022 Mar 14;298(4):101826. doi: 10.1016/j.jbc.2022.101826 (PMC9010760; doi:10.1016/j.jbc.2022.101826)

# Supplemental Figure 1

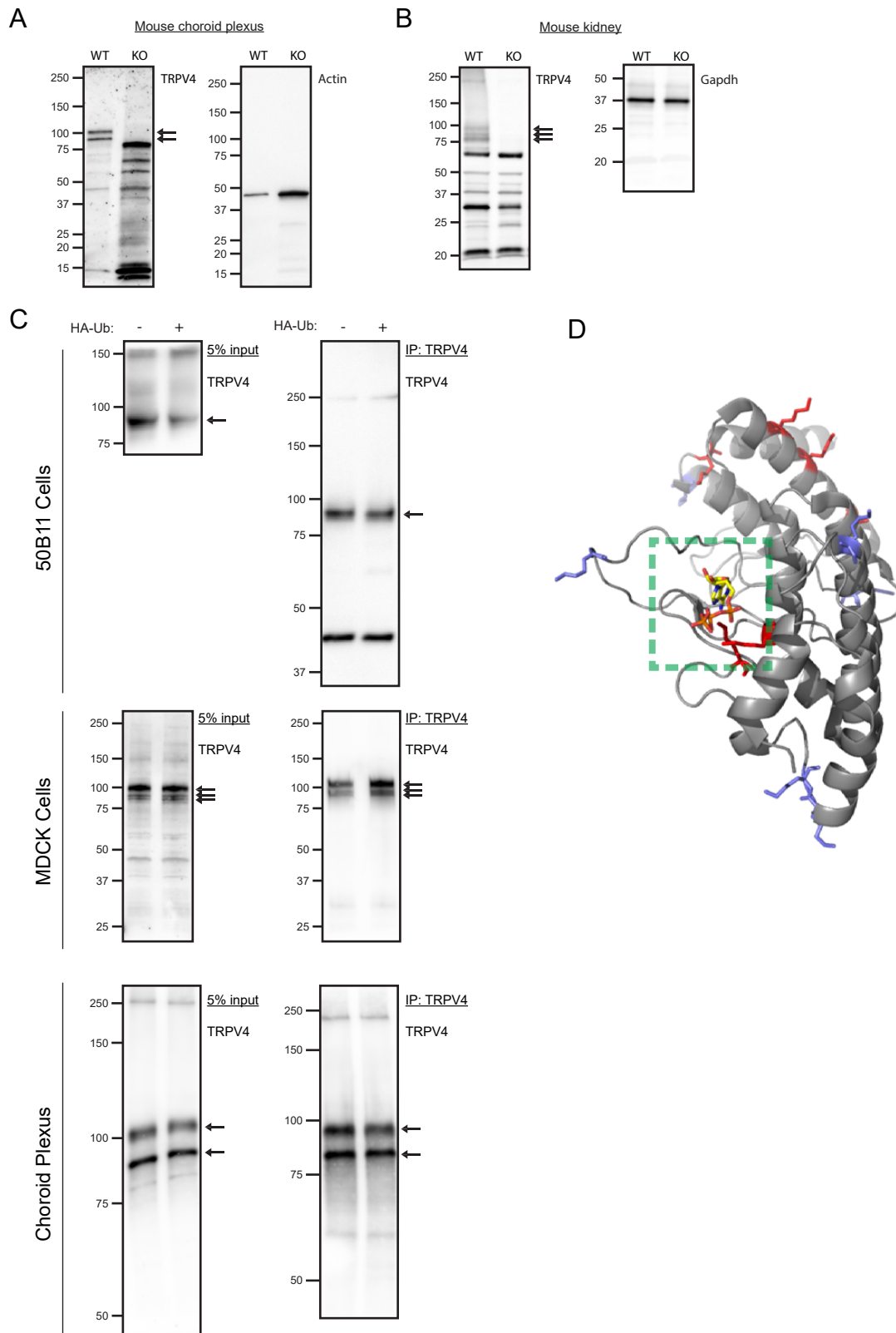

Supplement: Supplemental Figure 1 [file mmc6.pdf]

# Supplemental Figure 2

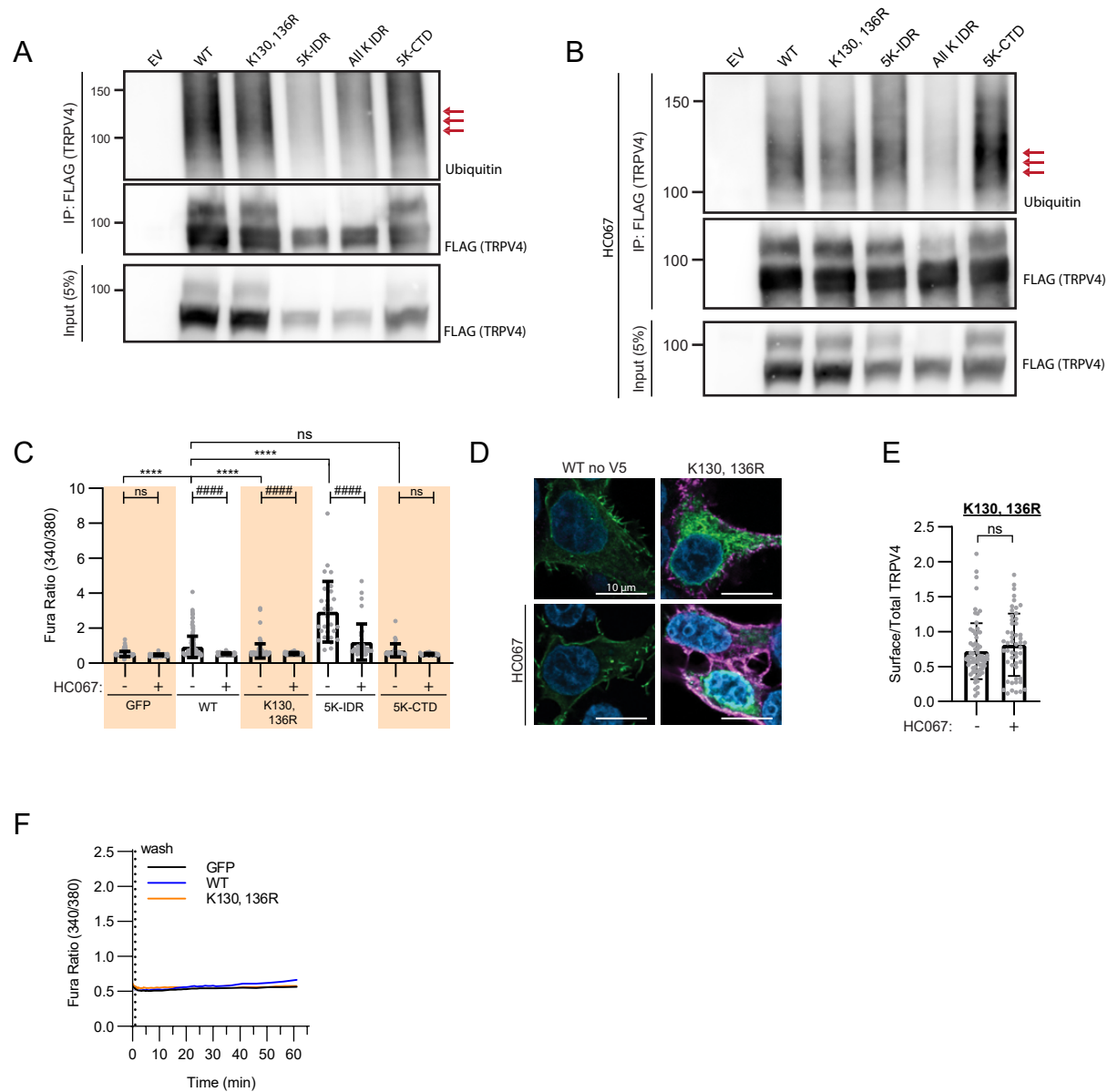

Supplement: Supplemental Figure 2 [file mmc7.pdf]

# Supplemental Figure 3

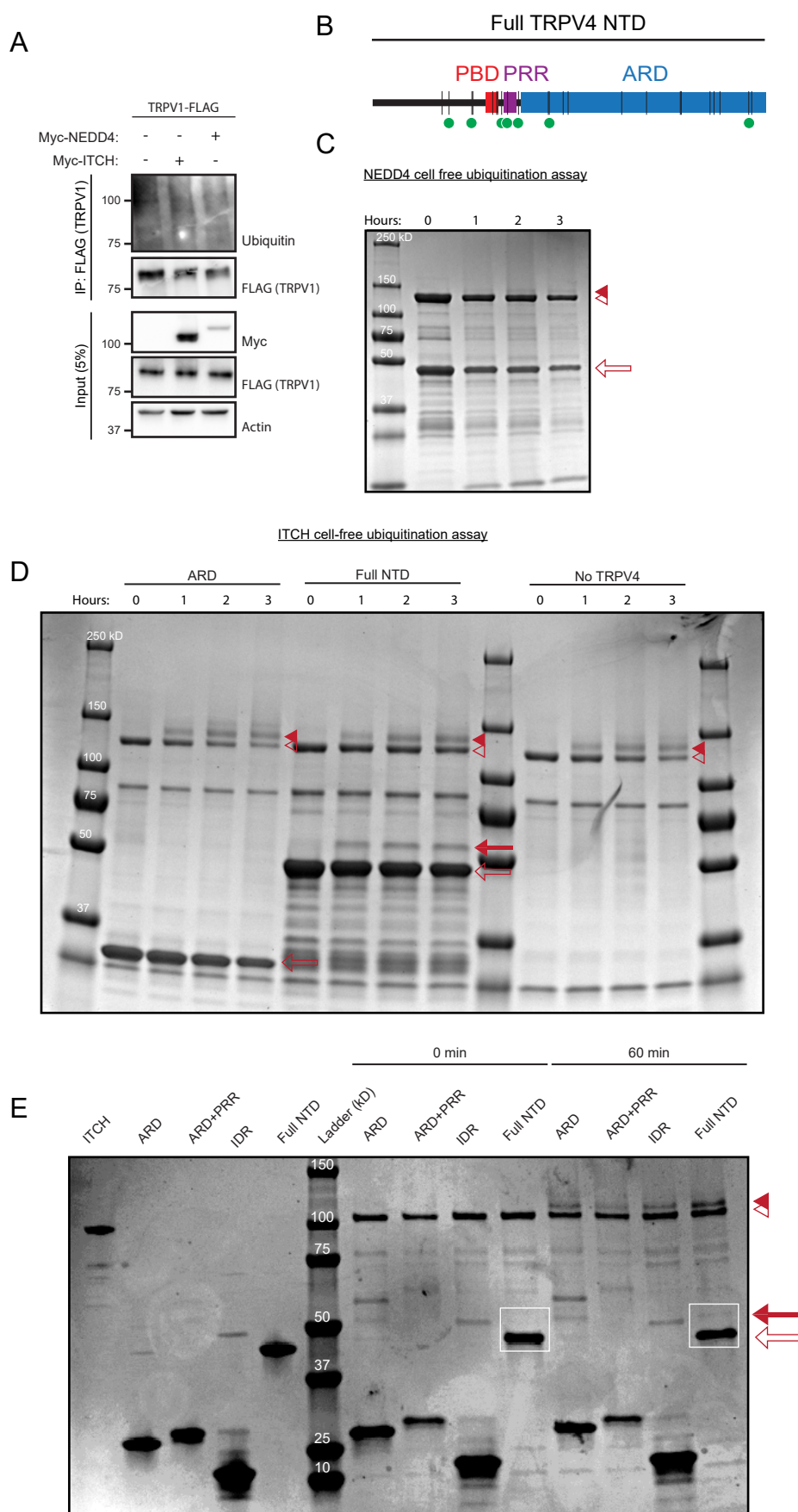

Supplement: Supplemental Figure 3 [file mmc8.pdf]

# Supplemental Figure 4

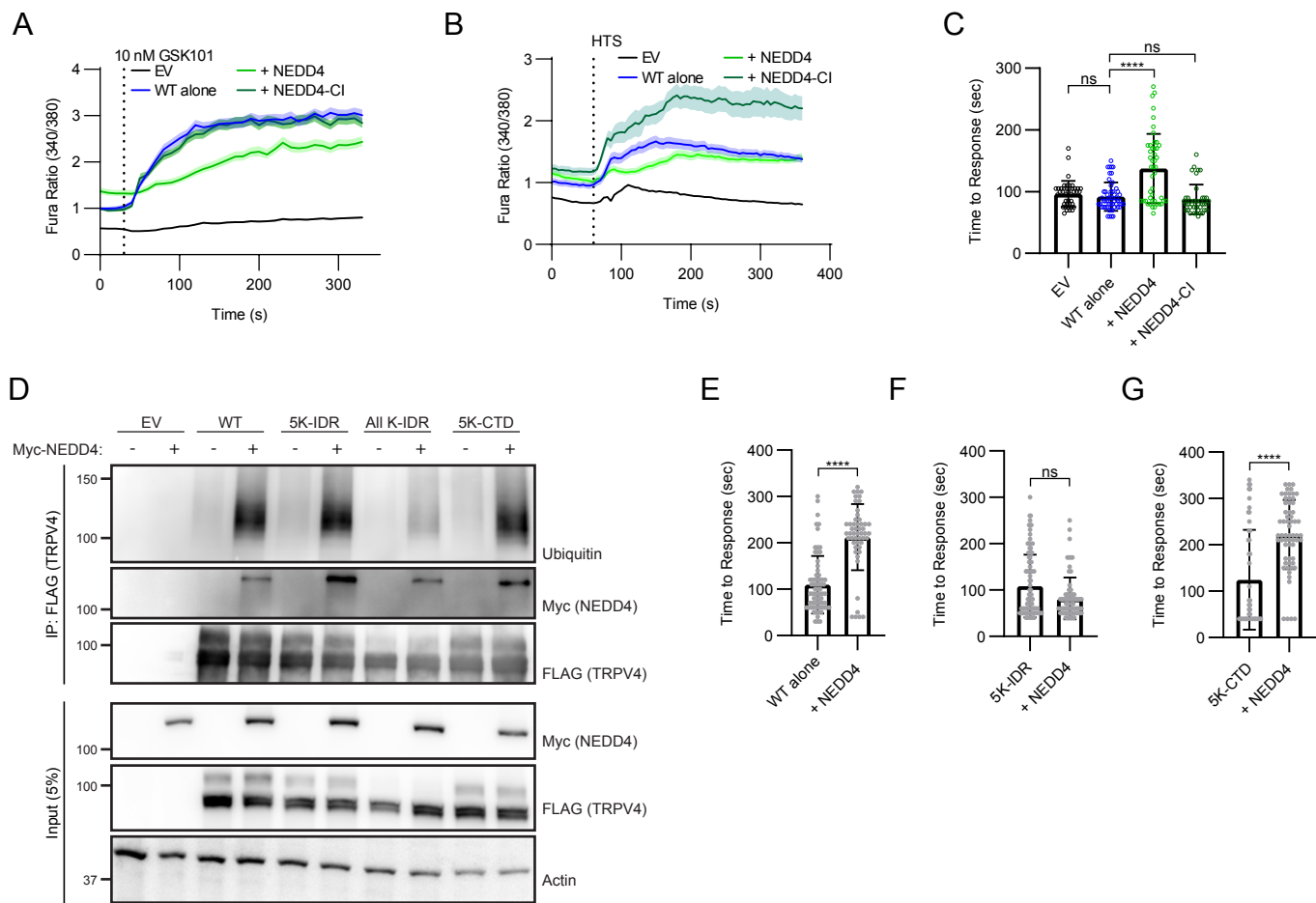

Supplement: Supplemental Figure 4 [file mmc9.pdf]

# Supplementary Figure 5

A

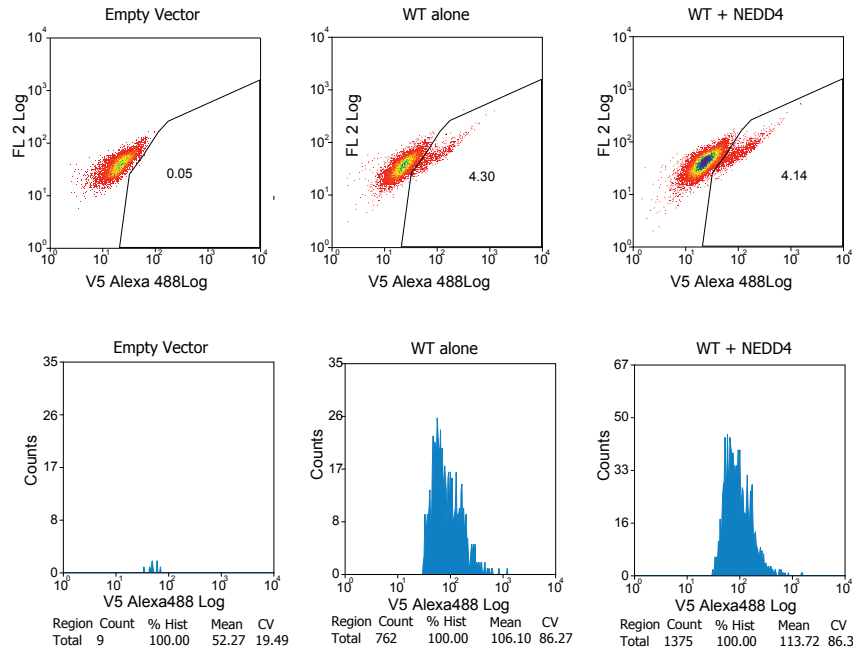

B

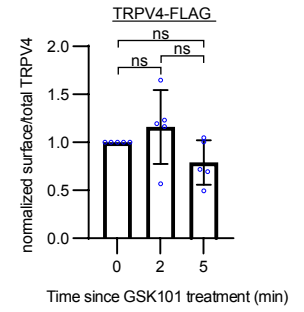

C

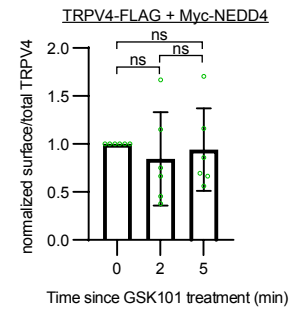

D

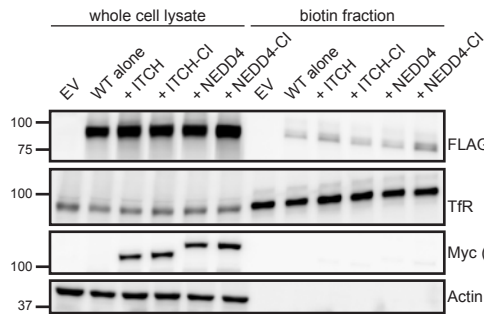

E

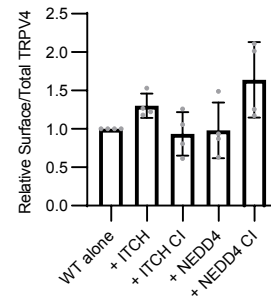

F

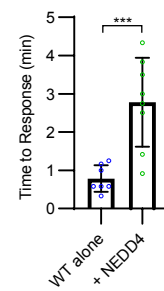

G

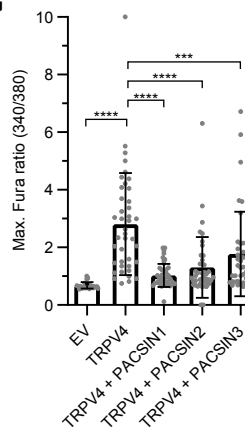

H

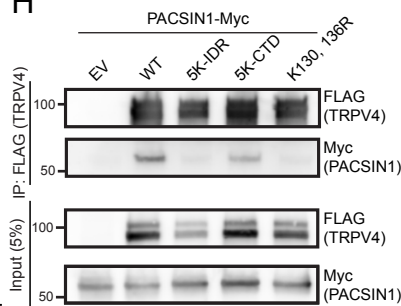

I

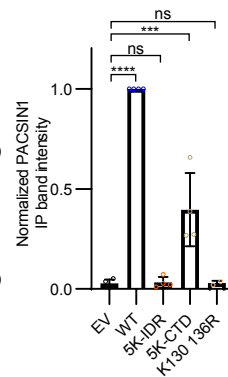

J

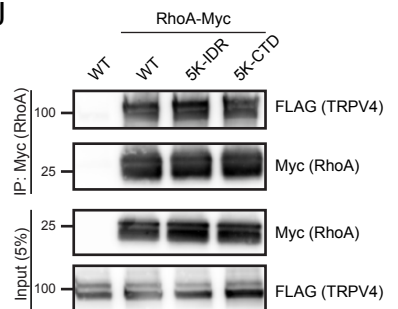

Supplement: Supplemental Figure 5 [file mmc10.pdf]

# Supplementary Figure 6

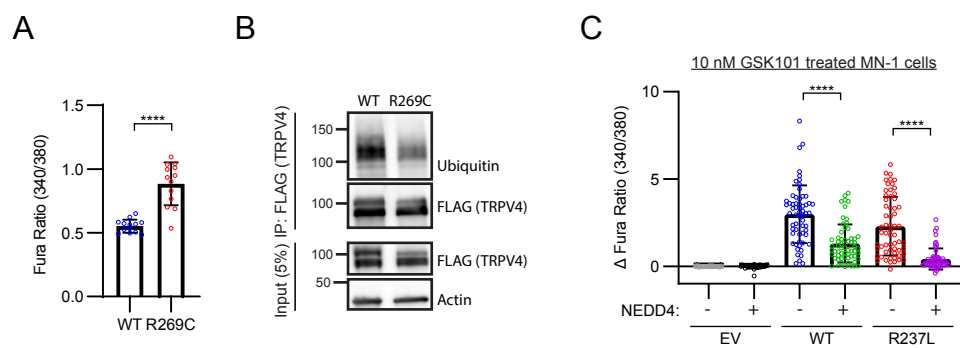

Supplement: Supplemental Figure 6 [file mmc11.pdf]
